# Field of genes: using Apache Kafka as a bioinformatic data repository

**DOI:** 10.1093/gigascience/giy036

**Published:** 2018-04-09

**Authors:** Brendan Lawlor, Richard Lynch, Micheál Mac Aogáin, Paul Walsh

**Affiliations:** 1 Department of Computing, Cork Institute of Technology, Cork, Ireland; 2 NSilico Life Sciences Ltd., Cork, Ireland; 3 Sir Patrick Dun Laboratory, Trinity Translational Medicine Institute, Department of Clinical Microbiology, School of Medicine, Trinity College Dublin, Dublin, Ireland

**Keywords:** Apache Kafka, scalability, parallelization, big data

## Abstract

**Background:**

Bioinformatic research is increasingly dependent on large-scale datasets, accessed either from private or public repositories. An example of a public repository is National Center for Biotechnology Information's (NCBI’s) Reference Sequence (RefSeq). These repositories must decide in what form to make their data available. Unstructured data can be put to almost any use but are limited in how access to them can be scaled. Highly structured data offer improved performance for specific algorithms but limit the wider usefulness of the data. We present an alternative: lightly structured data stored in Apache Kafka in a way that is amenable to parallel access and streamed processing, including subsequent transformations into more highly structured representations. We contend that this approach could provide a flexible and powerful nexus of bioinformatic data, bridging the gap between low structure on one hand, and high performance and scale on the other. To demonstrate this, we present a proof-of-concept version of NCBI’s RefSeq database using this technology. We measure the performance and scalability characteristics of this alternative with respect to flat files.

**Results:**

The proof of concept scales almost linearly as more compute nodes are added, outperforming the standard approach using files.

**Conclusions:**

Apache Kafka merits consideration as a fast and more scalable but general-purpose way to store and retrieve bioinformatic data, for public, centralized reference datasets such as RefSeq and for private clinical and experimental data.

## Key Points

- Big genomic datasets are generally available in formats that are either very general but slow to access or faster to access but too specific to a given algorithm. Taking National Center for Biotechnology Information's (NCBI’s) Reference Sequence (RefSeq), for example, it’s either available via the search interface itself (fast but specific) or as flat fasta files that can be FTPed (file transer protocol) from NCBI.
- Apache Kafka offers a unique architecture that can be harnessed by bioinformatic organizations to make data available at high speed, in a flexible manner, without committing too early to an algorithm-specific structure.
- Apache Kafka is persistent; it stores its data in a distributed and replicated fashion on contiguous disk space using an append-only log data abstraction. It is also fluid; data can be continuously updated and "compacted" in order to keep the data "live."
- We measured the speed and scalability of Apache Kafka in relation to flat fasta file access from RefSeq to give a sense of the gains that can be made thanks to this optimized structure.
- Given these characteristics and its excellent integration with complementary tools such as Spark, Flume, NiFi, Hadoop, and others, we propose Apache Kafka as a "data nexus" that bridges the gap between low-structure/low-speed and high-structure/high-speed solutions. We feel this is suitable to larger organizations to store either public or private genomic databases for general-purpose processing or for easier transformation to more specialized data formats.

## Background

Bioinformatic data are available from a number of authoritative organizations. One such example is the Reference Sequence (RefSeq) database, which maintains records of genomic DNA for model organisms [1]. RefSeq is maintained by the National Center for Biotechnology Information (NCBI),and its website includes three mechanisms for accessing the data: by searching for sequences using the Basic Local Alignment Search Tool (BLAST) program, by downloading the database files used by BLAST, and by downloading the underlying fasta files used to create the database.

BLAST is an invaluable tool for bioinformaticians who are searching for a particular genetic sequence [2]. It performs an alignment of a query sequence against a database of known sequences, returning results based on similarity. However, BLAST is a search engine, not a database. If the data are presented only through this search engine, all we can do with that data is search.

On the other hand, if we have access to the raw data, we can process it in any way we need in order to answer biological questions. NCBI provides a means for retrieving the underlying raw data by accessing the anonymous file transfer protocol (FTP) server (ftp://ftp.ncbi.nlm.nih.gov/), navigating to a database folder, and downloading the contents. In the case of RefSeq, this means downloading (at time of writing) 217 files of approximately 1 GBeach. Each file must be unzipped and untarred in order to reveal a number of BLAST-specific binary files. Those binaries can then be converted into text fasta files by means of a Linux-based BLAST command-line tool supplied by NCBI [3]. Each 1-GB download expands to around 4 GBwhen converted to fasta format, so the result of downloading RefSeq is almost 1 TBof data spread across a few hundred fasta files, stored on a local drive.

This structure limits the usefulness of the data, in particular, by hindering parallelization. If we wish to process every sequence in a group of fasta files, our parallelization factor is limited to the number of files, as each file must be read from start to finish by one process or thread. Here, we measure these structure-based limits and present an alternative, using Apache Kafka to overcome them.

### The structure of data

Data may be presented in many different ways for different users, depending on who they are and what they want to do with the data. This structure constrains the way in which such data may reasonably be used.

One characterizing quality of bioinformatic data is that the data are vast and growing. From an engineering perspective, the only rational way of processing this data is in a parallel fashion. Consequently, our data should be structured in a way that facilitates this.

What are the properties of such a structure? We propose the following nonexhaustive list of such properties.

#### Distribution

In order to free the data of hardware bottlenecks such as network adaptors, central processing units (CPUs), and memory, parallel data should be distributed across multiple machines. This is analogous to exposing the largest possible working surface area of data, in contrast to the limiting effect of storing all data on one physical server. To use a biological metaphor, when data are not spread out over a network, it is like a gene whose DNA is supercoiled around its histones and so cannot be expressed.

#### Reliability

It is a property of distributed systems to be both more and less reliable, in the sense that they no longer have a single point of failure (more reliable), but on the other hand, there are more elements that can break (less reliable). Distributed data’s structure should protect it from this latter effect while promoting the former.

#### Streaming

The advantage that streaming brings is that consumers of a stream do not have to wait until all data are downloaded before beginning to process that data. An everyday example of this is Netflix, the Internet streaming service for movies. In contrast with previous models of download-and-view, where the movie must be downloaded entirely before viewing can begin, with Netflix it takes 90 minutes to watch a 90-minute movie. The streaming advantage is particularly relevant for biological data that are often processed in pipelines, i.e., serialized stages of processing where the output of one stage acts as the input to the next. Although not all bioinformatic processing can be performed on partial data, much of it can, and by including this element of streaming, we allow for an extra dimension of parallelization when processing genomic data.

### Apache Kafka

Apache Kafka [4] is not commonly considered to be a database. It is generally viewed as a message broker, i.e., an intermediary program that transfers messages (general-purpose units of information) either asynchronously or synchronously from one program to another via a topic. In this capacity, it has been identified in previous research as a suitable technology for bioinformatic applications [5]. However, Kafka’s developers at LinkedIn implemented it with a wider scope of usage in mind, including “source-of-truth data storage” [6]. Kafka’s topics are implemented as (distributed) transaction logs, an abstract data type to which new data are only ever appended, never overwritten. Moreover, Kafka topics can be configured to never expire; this means that the same data can be read over and over again. Topics are stored in contiguous disk space, much like files, conferring important speed advantages. However, a single topic may be traversed by a number of co-operating readers in parallel. These features combine to allow Kafka to operate as a data repository with extremely high read and write speeds.

In the following paragraphs, we describe some of the features of Kafka and explain how they confer the parallel properties we seek.

#### Consumers and producers

A Kafka installation is comprised of a cluster of Kafka brokers on which independent producers (writers) and consumers (readers) operate. The same piece of software can be both a consumer and a producer. We comment further on the importance of this fact later in the article.

#### Partitions

**Figure 1:**
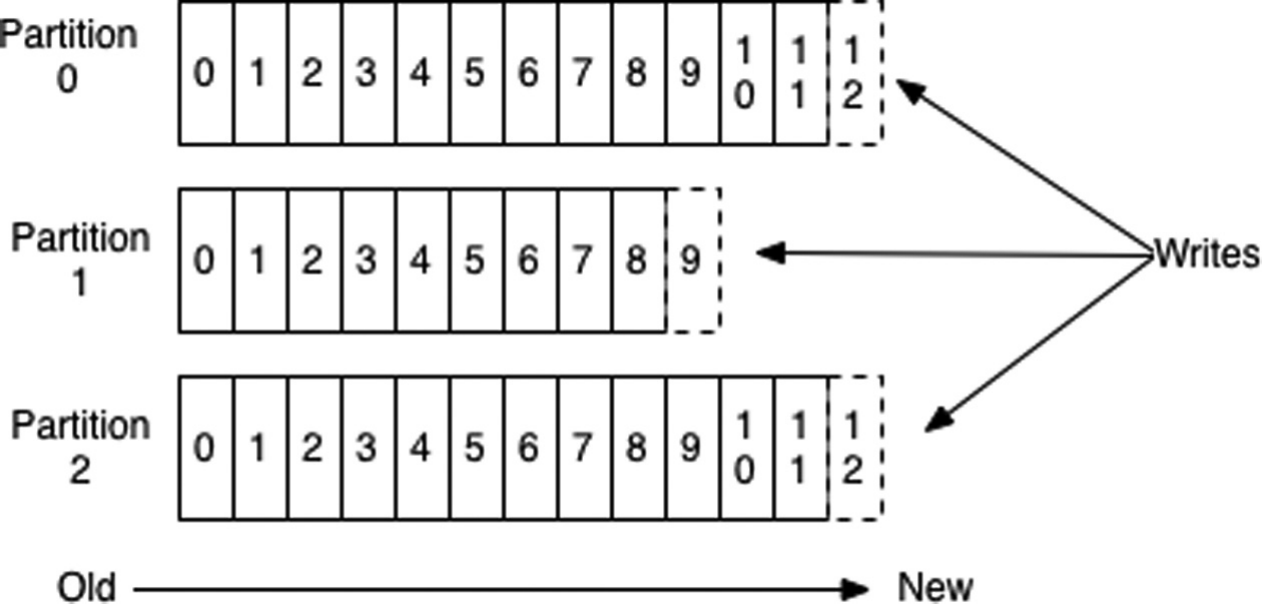
Anatomy of an Apache Kafka topic and partitions (from the Apache Kafka website).

Topics are normally single entities in message brokers, but in Kafka, they are divided into partitions, as shown in Fig.1. The partition is the unit of parallelization. If a topic is configured to have *N* partitions, then *N* consumers can read independently and in parallel, but in concert, from the same topic. Topics can also be configured to have a replication factor, *R*. This is the number of copies of a partition maintained across the cluster, so that if up to *R*− 1 machines in the cluster fail, no data are lost. For example, if a cluster has seven nodes and a replication factor of 3 for all topics (i.e., each partition has 3 copies, each on a different machine) then a loss of any 2 machines (*R*− 1) cannot delete all copies of any partition. Importantly, partitions also confer scalability. Topics can become arbitrarily large, holding more data than any given machine in the cluster can permit.

#### Consumer groups

**Figure 2:**
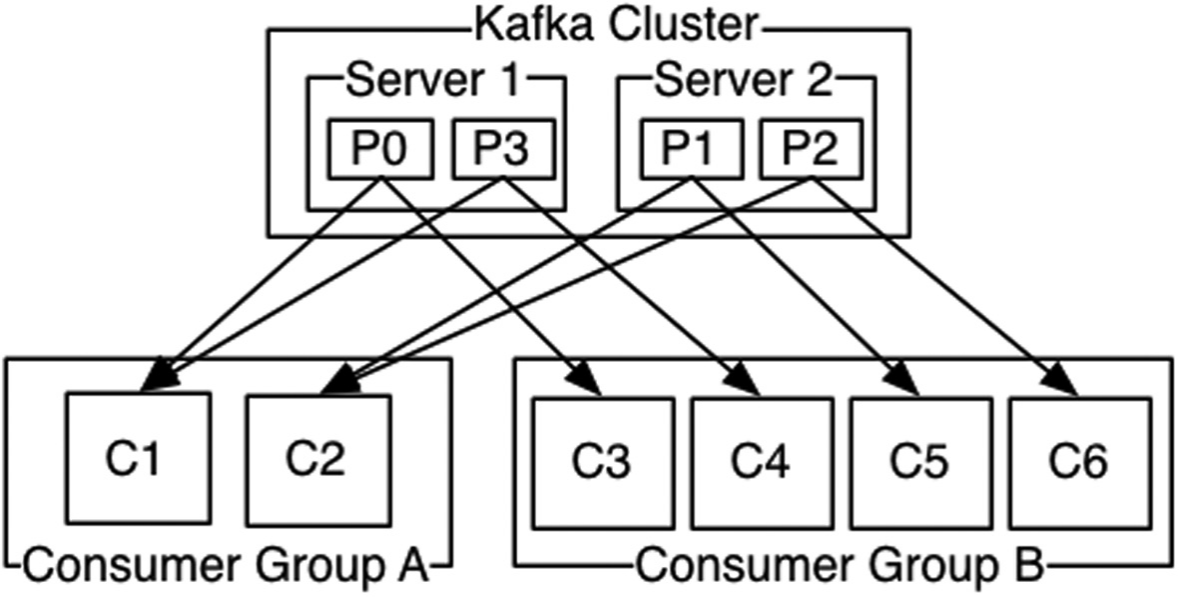
Consumer groups (from Apache Kafka website).

Another important feature of Kafka is the concept of a consumer group. Figure 2 shows a small cluster with two servers and a single topic broken into four partitions. Kafka allocates one partition each to the four consumers of consumer group B. However, in consumer group A, where there are only two consumers, each consumer is given two partitions to read. This organization allows a group of consumers to collaborate in order to "drain" a topic in parallel.

Another important thing to note is that any given partition is only read by one consumer within a consumer group at a time. This means that the order of reading is preserved for any given partition. Because order is preserved, we can know when a partition has been completed by adding a "back marker" at the end of each partition. This adds another useful element to Kafka-as-a-data-store. We can do an exhaustive sweep of a topic and know when we have touched everything.

#### Producers and message keys

In contrast to consumers that are dedicated to one or more partitions, producers can insert data into any partition of a topic. Every message is composed of an optional key and a value. Producers use a partitioning strategy to select a destination partition based on the key of the message or they can choose a random partition where no key is present. This strategy can be customized by any given producer.

#### Log compaction

Another important and useful aspect of message keys is their role in log compaction. As mentioned earlier, messages are continuously appended to partitions and do not overwrite old values. However, Kafka has a mechanism for dealing with cases where new values for old messages are sent or where messages are deleted. This is called log compaction and works as follows: on a scheduled basis, Kafka will recopy a partition, moving from oldest to youngest message and removing any messages that have a younger version (based on key identity). Deletion of messages is brought about when the youngest message with a given key has a null value.

Thanks to this mechanism, Kafka can continue to append new messages to topics without the topic growing indefinitely. In other words, in contrast to using files, new and changed data can be made available to users without obliging them to download very large files that contain very few changes. Of course, where new keys are added, the amount of space needed will increase over time, and Kafka caters to this by allowing new brokers to be added to the cluster and then rebalancing the load. This aspect of Kafka entails a certain level of complexity in the management of the cluster and this is discussed in the Discussion section.

#### Streaming

Finally, the Kafka application programming interface (API), which is available in the Java language, includes support for streams. Moreover, many other frameworks and platforms, such as Flume and Spark, have developed their own stream-based Kafka integration libraries.

More recently, Kafka libraries have emerged to support the Reactive Streams API [7], which includes the automatic management of back pressure when chaining many streams together into a pipeline. As part of our research, we used a Scala-based library from the Akka framework 8].

## Methods

In order to measure the performance characteristics of a Kafka-based genomic data repository with respect to flat files and also to understand what other properties might emerge from such an implementation, we produced a proof of concept that loads up to 11% of the RefSeq database into a single Kafka topic, spread over 4, 8, and 12 cloud servers. Each message in the topic is either a full sequence or a part thereof in the case of sequences larger than 100,000 nucleotides. In order to convey the image of the extended "‘working surface area" that we seek to create, we named this proof of concept "Field of Genes." What follows is a description of how to build the Field of Genes and how to measure its performance and scalability. Measurements of its performance with respect to the use of flat files (the *de facto* alternative) are presented in the Results section.

### Field of Genes

To facilitate reproduction of our findings for independent verification, we made extensive use of Docker to deploy Field of Genes. While it is outside the scope of this article to describe Docker in detail, we refer readers to a number of articles that propose the wider use of this technology to enhance reproducibility in bioinformatics [9-12], an issue that we previously highlighted [13].

What follows are the steps taken to create and populate the Field of Genes and to measure its performance and scalability. Where appropriate, we indicate the name of corresponding software modules under source control [14].

#### Computational fabric on the cloud

The data storage and computational fabric for Field of Genes is built on a Kubernetes cluster [15] using Google Container Engine (https://cloud.google.com/kubernetes-engine/). We created clusters of hosts (4, 8, and 12, depending on requirements), each with 8 vCPUs, 30 GB of memory, and 750 GBof solid state disk hard drive space, using gcloud (https://cloud.google.com/sdk/gcloud) from a Linux command line.

#### Kafka cluster deployment

On this Kubernetes cluster we deployed an equal number of Kafka broker instances. We were able to use off-the-shelf Docker images for Kafka, so no extra coding was required. We used open source examples of Kubernetes instructions to manage the deployment.

#### NCBI file upload java library

We then developed Java library code (module loader) to download, untar, unzip, and convert a single RefSeq file from NCBI.

#### Scala/Akka loader agent

Finally, we used the Scala language and the Akka streams API library to create a loader agent. This is a small autonomous program that processes instructions from one Kafka topic, uses the Java library to download the contents of a single NCBI file, and then sends the downloaded sequences to another Kafka topic (loader-agent module). We chose Scala and Akka because they are suited to parallel and streamed programming. However, given the wide range of programming platforms that have good Kafka integration, the agents could have been implemented in one of many other ways (e.g., as Flume agents or using Spark).

**Figure 3:**
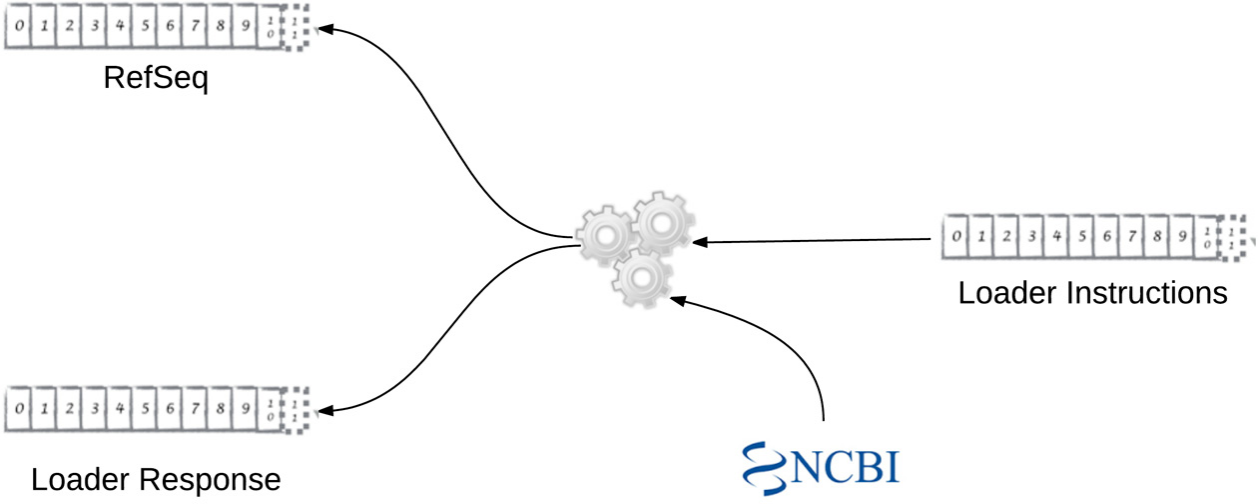
NCBI download agent.

Figure3 is a representation of this agent. It shows a process that downloads from the NCBI FTP site and pushes sequences to a RefSeq topic. The figure also shows that Kafka topics have been used to send instructions to the agent. When choosing how to send instructions to the agents and how to receive responses, it made sense to use the Kafka infrastructure already in place. This design decision had interesting beneficial effects that are examined in the Emerging Characteristics subsection.

#### Scala/Akka loader agent deployment

Using Kubernetes, we deployed the loader agent using different levels of parallelization, 4, 8, 12, 16, 20, and 24, to obtain an indicative download speed for each level, as well as to test for linearity of scalability. Note that as we raised the level of parallelization, we similarly increased the amount of data to be processed by increasing the number of downloaded files. We are measuring how well the benchmark and the Field of Genes can adapt to high load. A scalable system should show a flat horizontal line for time taken as both load and parallelization are increased in tandem.

Each agent was part of the same consumer group with regard to the loader instructions topic that, as explained in the Apache Kafka subsection, means that the partitions of that topic were evenly allocated across the agents. We set the number of partitions of the loader instructions topic to be the same as the level of parallelization.

The producer responsible for writing to the loader instructions topic used incremental numeric keys for the messages and relied on the default partitioning strategy (also explained in the Apache Kafka subsection). In this way, we could be confident that the instruction messages were spread evenly across the partitions.

These configurations combined to ensure the most efficient spread of data and computation across the cluster.

#### Loader measurement and benchmark

For each level of parallelization, we measured the time elapsed from when the first loader instruction message was sent to when the last RefSeq sequence was written to its destination topic.

Our benchmark for comparison was a single server of exactly the same specifications as those used by Field of Genes, using multithreading to achieve whatever levels of parallelization the system could support. Our hypothesis was that by spreading the genomic data over a wider "working surface area," we can attain levels of parallel access and scalability that more than compensate for any performance loss due to transmitting data between machines. Therefore, this benchmark architecture, i.e., a single server where no network traffic is required and no streaming is performed, is appropriate.

The Docker technology allowed us to reproduce the same server environment and change only the software components under test. This was accomplished by creating a single Docker host and deploying a single container that, with varying numbers of threads, downloads from NCBI using the same Java library the Field of Genes agents used. Note, however, that due to the limitation of using a single node, compared to a cluster, we were not able to run the benchmark to the same levels of parallelization as with the experiment. As will be seen from the results reported in the Results section, we still arrive at a useful comparison of absolute performance and relative scalability between the benchmark and Field of Genes. The very fact that we were limited in how parallel we could make the benchmark is an indication of the problem we are addressing and the suitability of our proposed solution. This is discussed in the Discussion section.

### GC content calculation example

The previous subsection described how the Field of Genes is constructed and populated with RefSeq data. In this section we describe one implementation of a bioinformatically useful agent that operates on that data. The ratio of cytosine and guanine bases (the Cs and Gs of the genetic code) to adenine and thymine (the As and Ts) is a biologically meaningful property of any given DNA sequence [16]. Measuring this value for a large number of sequences is an example of a parallel processing problem and therefore is suitable as an initial test for the Field of Genes, as well as a good placeholder for more sophisticated processing (gc-content module).

#### Gc-content agent

**Figure 4:**
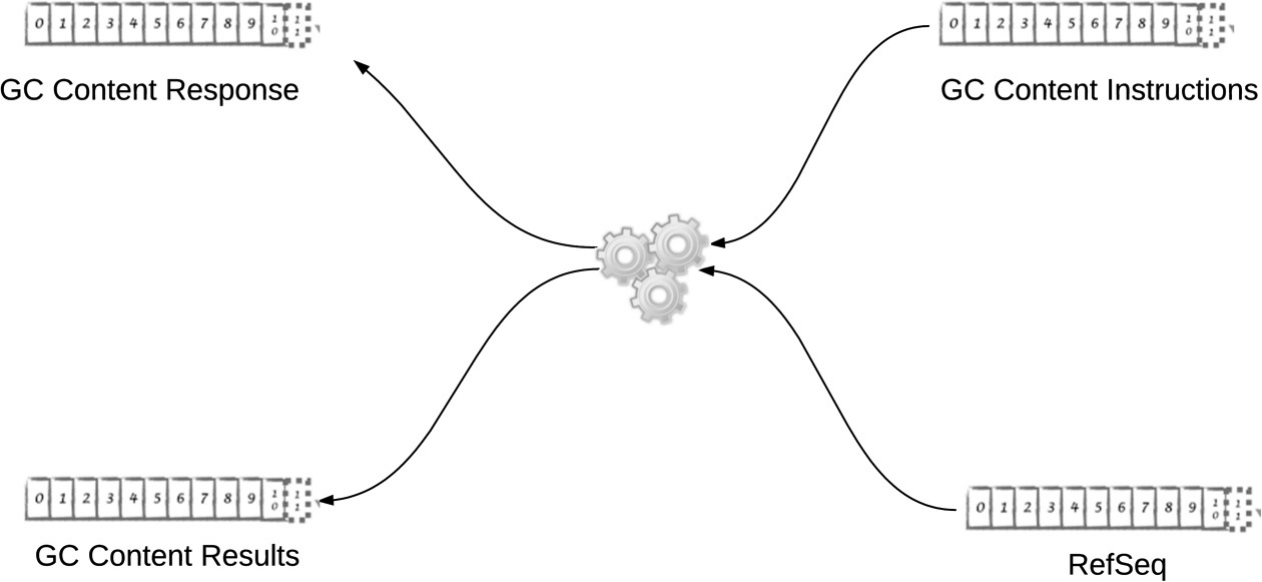
GC content agent.

The elements of this implementation are very similar to the loader agent elements of the previous subsection. We construct and deploy (using varying levels of parallelization) independent agents that consume from some topics and produce into others (gc-content-agent module). Figure 4 gives an overview of this agent.

From this we can see that the output of the loader agent has become the input of the GC Content agent, hinting at the opportunity to parallelize these tasks into a streaming pipeline, as discussed earlier. Moreover, there is another discernible opportunity here, which is the pipelining of responses and instructions. We explore some aspects of this in the Emerging Characteristics subsection.

#### GC content agent deployment

As with the loader agent experiment, we used Kubernetes to deploy the GC Content agent with increasing levels of parallelization. In each case, we set the number of GC Content Instruction partitions to be the same as the level of parallelization.

#### GC Content measurement and benchmark

The measurements taken for GC Content using Field of Genes are used to gauge if this architecture leads to improved performance and scalability. As with the Loader experiment, we compare Field of Genes to a multithreaded, single-server solution using a single instance of precisely the same server configuration. For the same reasons given in the section on the loader agent benchmark, we consider this a valuable comparison. Again, our measurements are made by increasing the parallelization factor and the amount of data to be processed, in tandem, looking for a flat system response.

The method of measurement for Field of Genes in this case is slightly different than the loader measurement. streams are effectively infinite sources of data. In order to know when a stream is "complete," we either set some "back-markers" in each partition to indicate that the end has been reached or we wait for the system to reach a kind of equilibrium where the output is no longer changing. The notion of equilibrium is discussed in the Discussion section.

We chose this latter approach for our experimental measurements. Using Kafka’s administration API, we regularly measured the size of the output topic. The stream was considered complete when its size did not change in a defined period of time; in our case, two consecutive periods of 10 seconds.

## Results

Table 1 shows the download time in seconds for the Benchmark (*DL*_*b*_) and Field of Genes with 8 and 12 servers in the cluster, respectively (*DL*_*FoG* − 8_ and *DL*_*FoG* − 12_). Each row shows the results for the same fixed number of RefSeq files and level of parallelization, threads in the case of the Benchmark and agents in the case of Field of Genes (*T*/*A*). Note that the last two rows of the Benchmark are empty as the single server did not have enough space to store 20 files or more.

**Table 1:** Download times (seconds)

T/A	*DL*_*b*_	*DL*_*FoG* − 4_	*DL*_*FoG* − 8_	*DL*_*FoG* − 12_
4	94	96	116	99
8	216	179	165	152
12	333	381	174	146
16	445	508	259	202
20			395	214
24			484	275

T/A: number of threads (Benchmark) or agents (Field of Genes). *DL*_*b*_: download on Benchmark. *DL*_*FoG* − 4_: download on Field of Genes cluster, size 4, etc.

**Figure 5:**
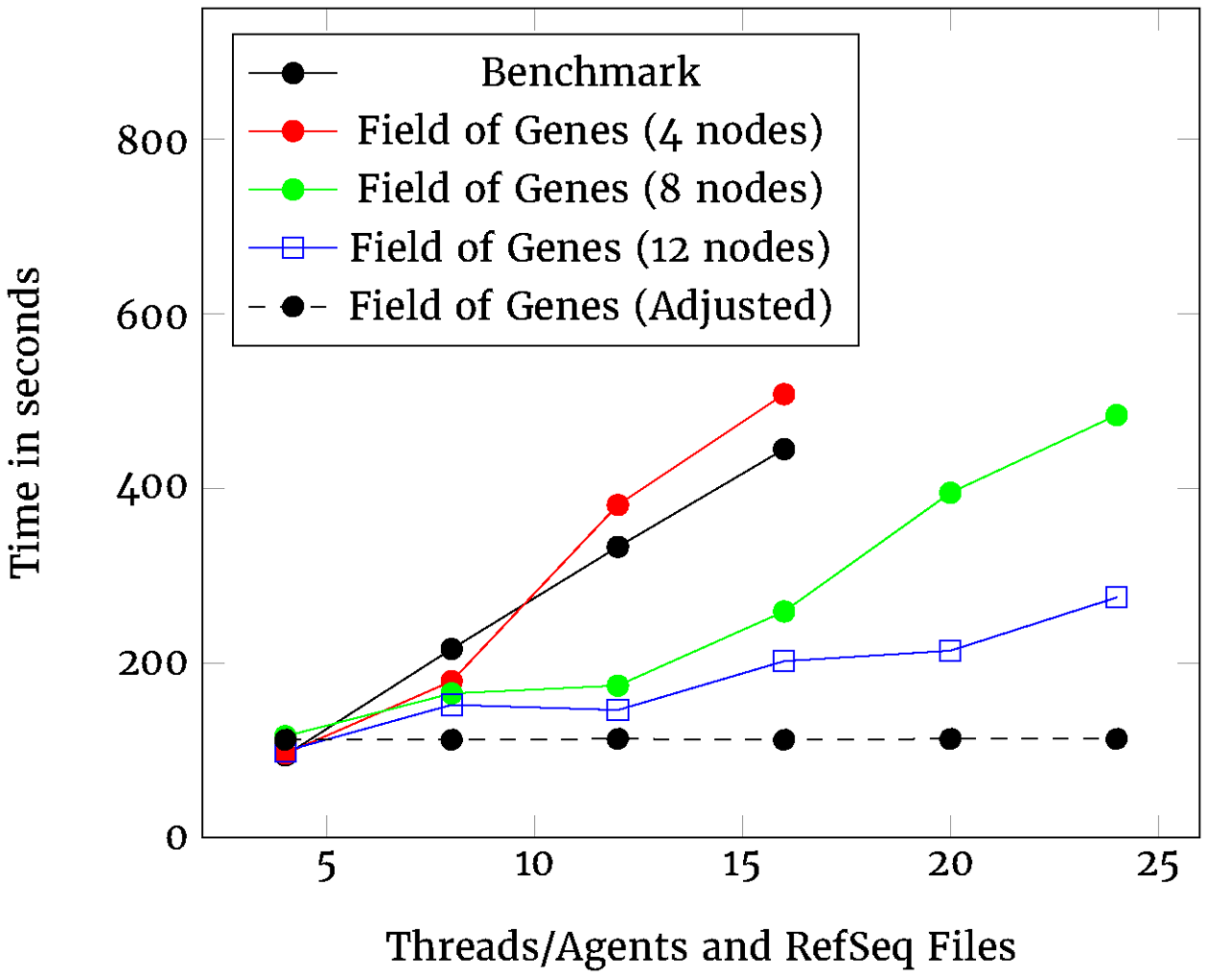
Scalability of download.

When we plot these download values with parallelization factors along the *X*-axis and download time on the *Y*-axis, as shown in Fig.5, we can compare how well the two options scale up. A flat horizontal line represents perfect scalability where the overall time to download does not change when extra data are added, as long as the parallelization factor increases to the same degree. In this kind of plot, the scalability can be seen to be inversely proportional to the slope of the line. The greater the slope, the less scalable the system.

**Figure 6:**
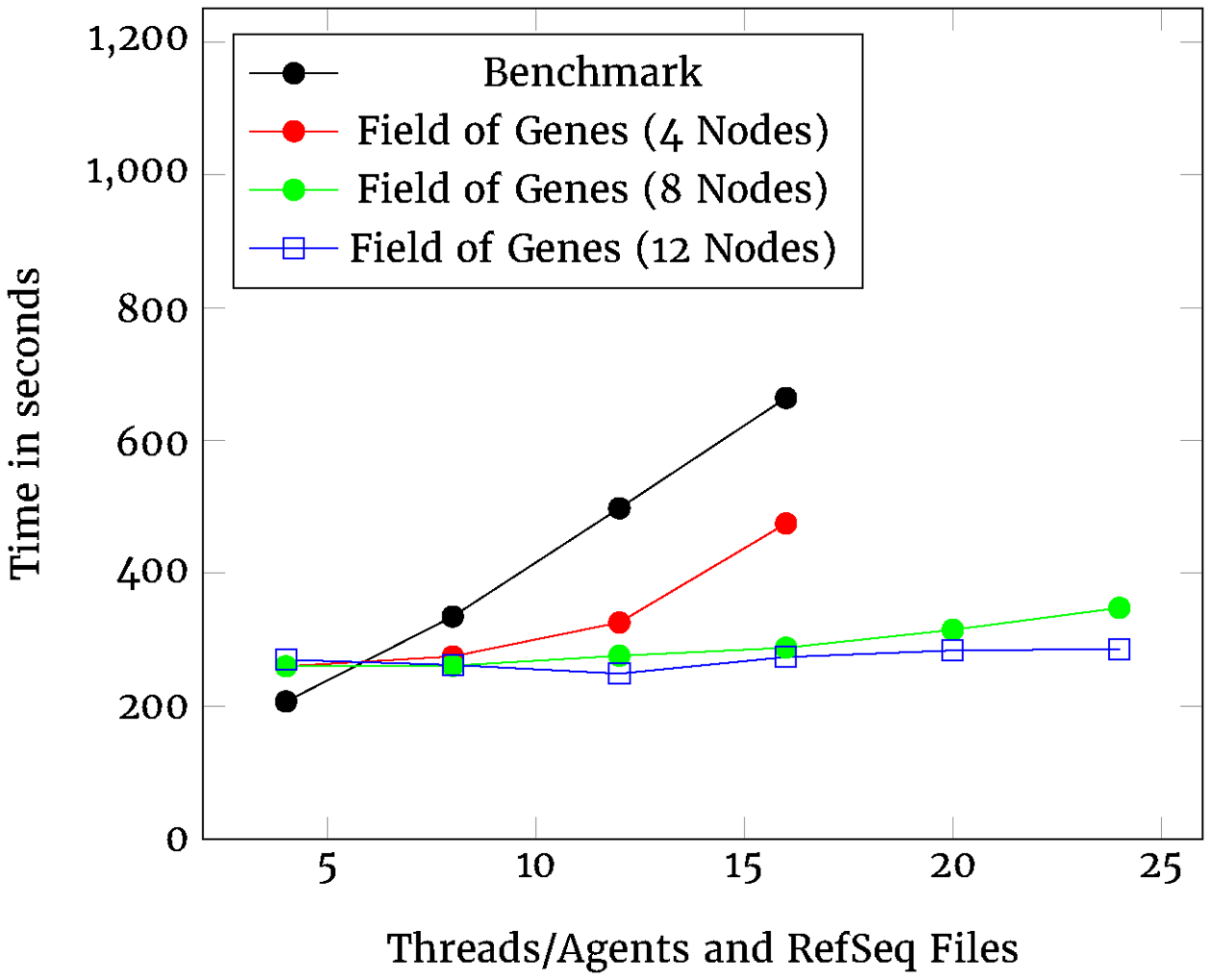
Scalability of GC content.

The same formats of data (Table 2) and plot (Fig. 6) are presented for the case of the GC content processing. In this case, we see a departure between the 8-node and 12-node cluster behavior. The limits of scalability for the 8-node cluster start to arise between 20 and 24 RefSeq files, for reasons discussed in the Discussion section. The 12-node cluster, however, is able to extend scalability further.

**Table 2:** GC content times (seconds)

T/A	*GC*_*b*_	*GC*_*FoG* − 4_	*GC*_*FoG* − 8_	*GC*_*FoG* − 12_	Sequences
4	207	260	260	270	1.56· 10^5^
8	335	275	261	262	3.1· 10^5^
12	498	326	276	249	4.58· 10^5^
16	664	475	288	274	6.16· 10^5^
20			315	284	7.7· 10^5^
24			348	286	9.27· 10^5^

T/A: number of threads (Benchmark) or agents (Field of Genes). *GC*_*b*_: GC content on Benchmark. *GC*_*FoG* − 4_: GC content on Field of Genes cluster, size 4, etc.

**Figure 7:**
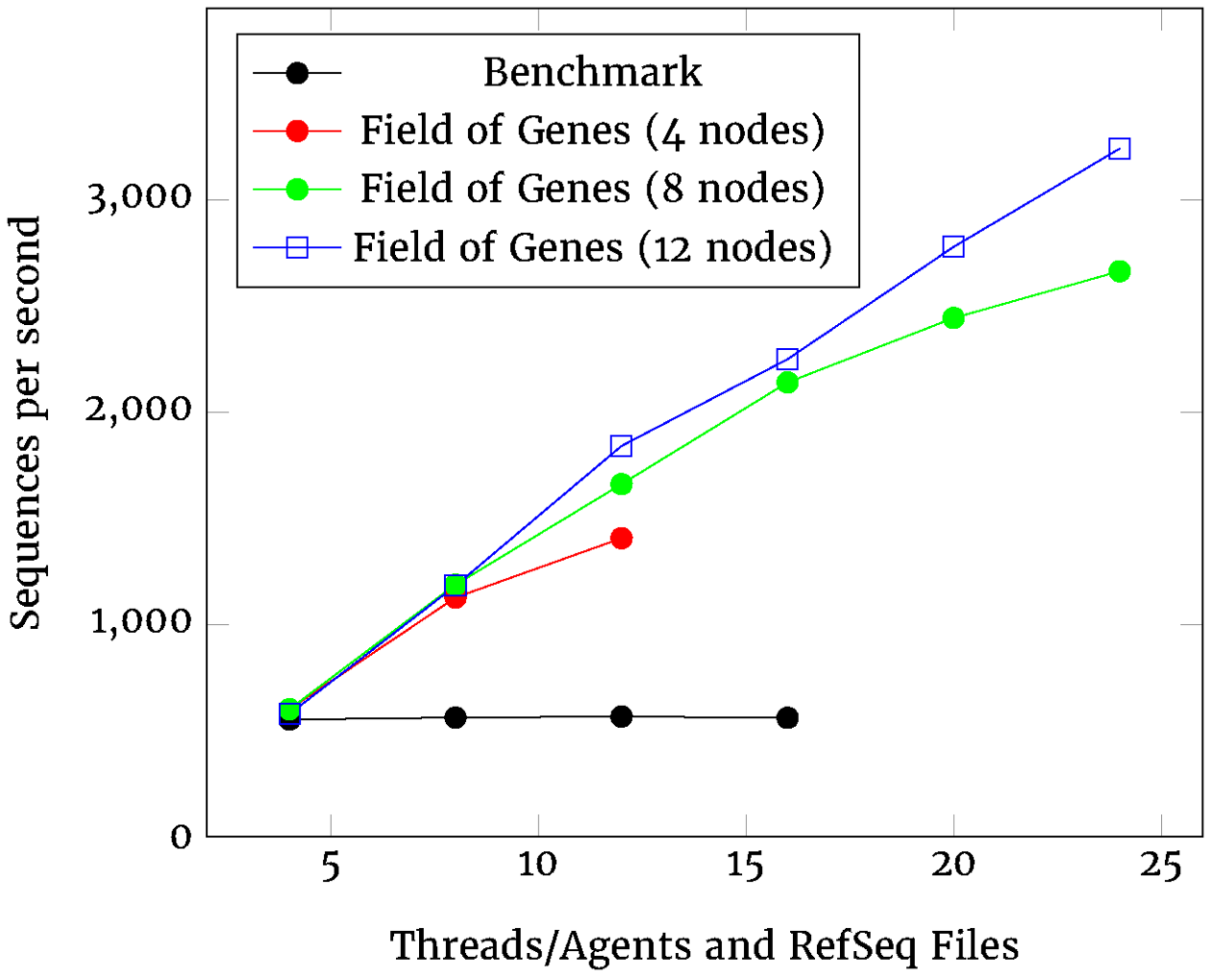
Processing rates of GC content (seq/sec).

Whereas the previous plots are designed to show scalability, Fig. 7 compares the raw performance of the Benchmark and Field of Genes systems using sequences per second as a metric (where sequences are strings of genetic code up to 100,000 characters long). In this format, the greater the value on the *Y*-axis, the better the system performs.

## Discussion

### Interpretation of results

The results presented in the previous section lead us to draw a number of clear conclusions.

When downloading data from NCBI, the two alternatives show very different characteristics. The Benchmark solution is similar to the Field of Genes with four nodes. The Field of Genes performance improves as the number of nodes increases. Note that while the Benchmark process downloads, unzips, and converts the RefSeq files from NCBI, the Field of Genes process does all this and then writes the sequences to Kafka.

The GC content processing presents a similar picture, but, in this case, even the four-node Field of Genes cluster performs better than the Benchmark. This advantage becomes more pronounced as the cluster size increases. The 12-node cluster shows an effectively flat response, indicating almost perfect scalability. This ability to arbitrarily extend performance by scaling out (distributing across more nodes) is one of the features of Kafka that makes it such a suitable repository for bioinformatic data.

### Emerging characteristics

Implementing any software system involves a certain amount of "on-the-fly" design. One can never know what the complete solution will be until the finer complexities have been encountered and dealt with in the code itself. This is what is meant by the term "emergent design", and it is a useful exercise to look back on any implementation, including (in fact, especially) proofs of concept, in order to see what else can be learned from the experiment beyond the original hypothesis.

In the case of Field of Genes, we would like to point out two unanticipated features that we believe may be worth building on.

First, we note that a system that uses Kafka to store data will also tend, for expediency, to use Kafka to store instructions. This is especially the case when large numbers of autonomous agents operating on the data need to be coordinated. An emerging feature of this tendency is the ability to pipeline not only the data but also these instructions, so that the results from one agent might trigger the behavior of another. As we do not wish to spend too much time predicting where this may lead, it is enough for now to point out something that every biologist knows: from many small and simple co-operating elements, very complex and intelligent pathways may be constructed.

Second, another feature of stream-based programming, which we touched on when describing measurement in the GC Content Calculation Example subsection, is the idea that a process in some sense may never be finished and, instead, arrives at equilibrium, at which point the most recent results may be read off a final topic. As new source data are fed upstream into such a system, it creates a ripple of computation, resulting in a refreshing of the final results. While this is not the behavior that is typically expected of software systems, in the era of big genomic data that is constantly changing, it may become accepted as a suitable paradigm.

### Drawbacks and alternatives

Given its characteristics, Kafka is not suitable for some applications and contexts. It is complex software that is designed to run in a cluster, so it can be difficult to deploy and maintain correctly. For this reason, at least for the time being, it is suitable only for teams with specialized software engineering skills. We believe that this situation will improve over time as Kafka becomes easier to deploy in a containerized setting. We also note recent commercial offerings of “Kafka as a Service,” (see https://www.cloudkarafka.com or https://www.confluent.io for examples) which would outsource this complexity.

There are alternative systems available whose characteristics overlap with Kafka. One such system is Apache Flume [17]. The overlap in their functionality relates to the fact that both Kafka and Flume are message brokers; they provide a channel for message producers and consumers to exchange data.

However, there are significant design differences that separate them. Most importantly, Kafka is designed to be persistent and replicated, making it suitable as a primary data repository. Flume typically uses in-memory queues, which lose data in the event of a failure. While Flume more recently includes a file-based channel, this is not replicated and so is only as reliable as the disks that it uses.

The point is that there are “horses for courses.” Flume should be considered primarily a platform for transporting and transforming data, especially data intended for Hadoop storage. Kafka, while also transporting general-purpose data, was designed from the outset to persist that data in a replicated fashion. We describe how these differences can be harnessed in a collaboration in the next section.

### Potential implications

Field of Genes presents a fast and scalable access point to raw data and has a structure that is independent of any particular algorithm. It is an open-ended system that can accept updates in real time and propagate those updates onward. Given this, it would make a suitable central repository or nexus into which bioinformatic data could be sent from a variety of sources and from which bioinformatic data could be accessed for a variety of uses. We envisage two kinds of usage scenarios.

The first scenario would be to use Kafka as an initial staging point (and potentially source-of-truth repository) of low-structure data, to be converted to high-structure data of different formats including, but not limited to, Cassandra and BLAST.

Kafka has good integration points with many other technologies, and so is well placed to act as a parallelized and streamed data nexus.

Upstream, a system such as Apache NiFi [18] or Apache Flume could be configured to load data into Kafka topics. Downstream, Kafka topics can feed into databases and computation platforms as diverse as Cassandra, Hadoop, Flume, Spark, and BLAST. For example, Kafka’s disk-stored topics could be the data source for Spark’s resilient distributed datasets (RDDs), an especially interesting use case when the RDD storage level is configured to be “memory only.” These high-structure platforms would serve as the basis for SQL-like queries (on Cassandra or on Hadoop with the help of Phoenix) or more specialized queries (such as BLAST searches). Such downstream high-structure data could be continuously updated as new data are appended to Kafka.

The contrasting features of Flume and Kafka can make them ideal partners in a single data ecosystem. Flume-Kafka integrations (informally “Flafka”) have been developed to make it easier to write Flume agents to act as producers and consumers of Kafka topics.

The second scenario would be the use of Kafka as a platform for performing high-speed, parallel processing directly on the low-structure data. The gc-content agents described here were developed in order to demonstrate this second usage scenario and also in order to demonstrate Kafka’s scalability. However, this scalability and parallelization would apply to any stream-based consumer of Kafka topics, even off-the-shelf systems such as Flume, and not just custom-built agents.

The technology we used for the agents, Reactive Streams using Scala and Akka, were designed specifically with Kafka in mind and have been previously described by the authors as suitable for genomic applications [19]. However, as stated above, many other suitably streamed and parallel platforms have good Kafka integration, freeing users to process the Kafka topics using their preferred technologies.

**Figure 8:**
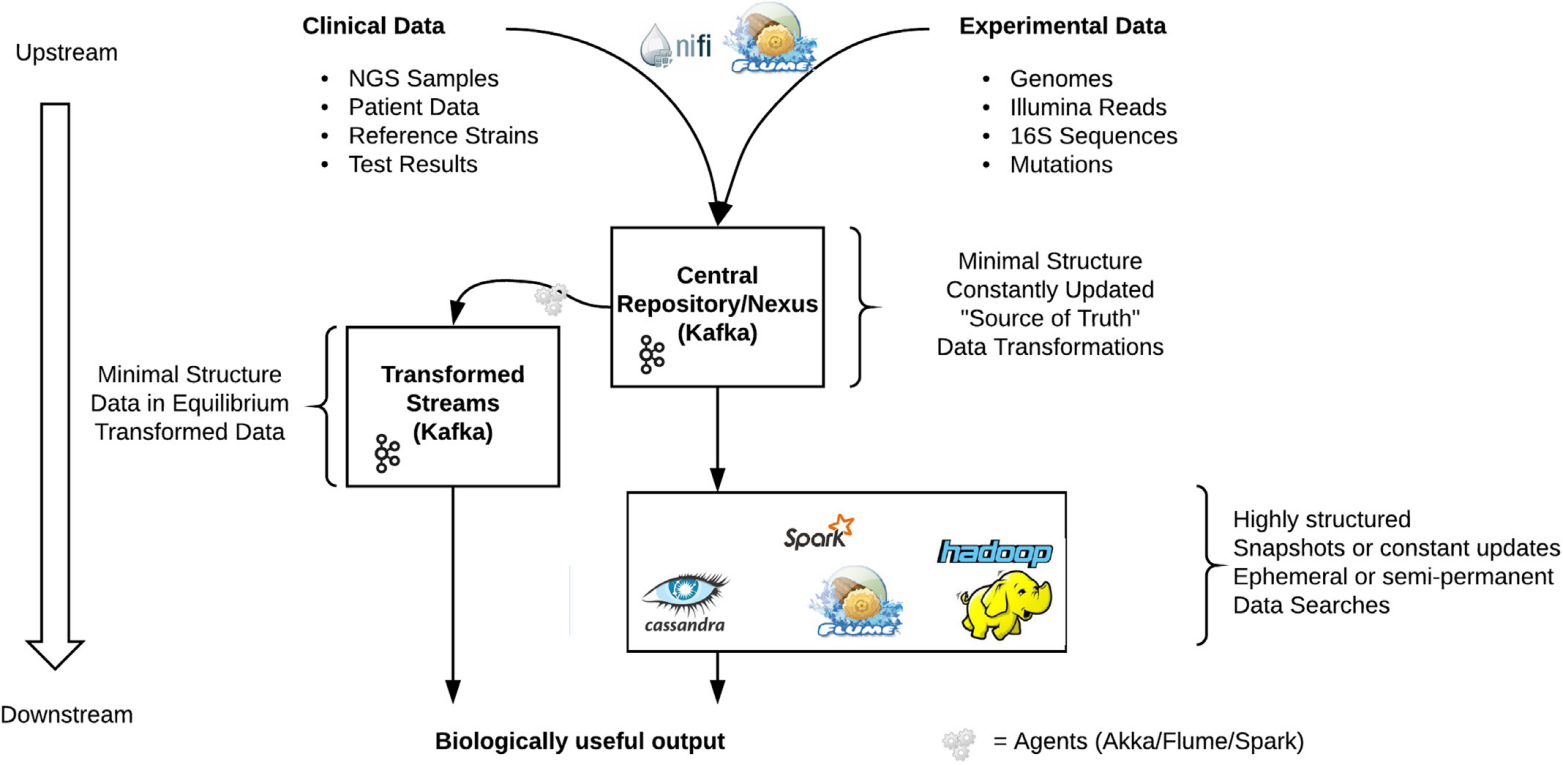
Example use context of Apache Kafka.

Figure 8 represents this dual approach. It shows Kafka as a central nexus of relatively unstructured data: the “source of truth.” These data can be processed in place, from one Kafka topic to another, or transformed into structures more suitable for specific algorithms.

### Suggested applications

The gc-content calculation on RefSeq in our experiment was chosen to serve as a placeholder for more meaningful work of the same nature: exhaustive, parallel transformations or searches on large datasets. Here, we consider some specific potential applications. The following list is intended to be indicative rather than comprehensive:

- *Variant annotation*: Kafka as a storage for genetic variants with a view to predicting phenotypes (e.g., disease risks in humans, antibiotic resistance in bacteria).
- *Differential expression analysis:* Perform clustering on sequencing reads (preservation of ordering is important).
- *Genomics*: Quality control and error correction of large genomic datasets.
- *Taxonomy*: Calculation of average nucleotide identity of query genomes against subject genomes.

To take the last of this list, the OrthoANI [20] algorithm finds average nucleotide identities between query and subject genomes by breaking both query and subject into fragments, finding query and subject fragments with reciprocally best matching nucleotide identities, and using only these reciprocal matches to calculate the average nucleotide identity.

In a Kafka implementation, one topic each could represent the query fragments and another the subject fragments. A consumer group could read the subject fragments topic to completion, and build a BLAST database using each fragment as a database entry. It would then stream the query fragment topic and for each query fragment, find the best match and its nucleotide identity, sending the resulting match (and nucleotide identity) to an output topic. A second consumer group would do the same thing, but opposite: reading the query topic to completion and streaming the subject topic.

The two resulting output topics, from the opposite processes above, would be treated differently. One would be read to completion to create an indexed lookup table in a distributed database such as Cassandra. The other would be iterated over by another consumer group, finding cases where the match is reciprocal. Reciprocal matches would be written to a final output topic from which a running average would be calculated. This running average would move to the OrthoANI for the query and subject genomes.

The above, necessarily brief, example demonstrates a number of advantages of using Kafka:

- A high degree of parallelization (set by the size of the consumer groups);
- A pipeline parallelization (each phase can begin as soon as output from the previous phase starts to arrive);
- Examples of data format transformation to more structured data when required (BLAST and Cassandra in this case)
- Examples of data being processed directly from the Kafka topics (the invoking of BLAST to find matches, and the calculation of running average)
- An equilibrium-based system (the calculation will tend toward the result, even before the processing is complete, which may be enough to decide whether, e.g., the query and subject belong to the same species).

### Further work

We chose to put RefSeq into a single topic rather than, e.g., dividing it across different topics based on taxonomic classification of the sequences.

Similarly, while we emphasized the processing of entire sets of genomic data, we made no mention of the fact that individual messages in Kafka can be directly accessed based on a three-values index: topic, partition, and offset. While these were reasonable choices, further research is needed in order to arrive at optimal design decisions related to this technology.

Creating working systems from proofs of concept is rarely trivial. In the case of Field of Genes, a distributed, cloud-based approach that tends toward points of equilibrium, this is especially true. Such complex clusters require a secondary management infrastructure for updating, monitoring, scaling, and similar processes. They suffer from particular issues that must be understood and remediated. For these reasons, further work will be required in order to implement a stable, public-facing platform based on the Field of Genes approach. Moreover, such an approach would be more suited, at least initially, to larger institutions with solid software engineering capabilities and resources.

## Availability of supporting data

A snapshot of the code is available in the GigaScience GigaDB repository [14].

## Availability of source code

- Project Name: Field of Genes
- Project home page: https://github.com/blawlor/field-of-genes
- SciCrunch RRID: SCR_016155
- Operating system(s): Linux/Docker
- Programming language: Java and Scala
- Other requirements: Docker
- License: Apache 2.0

## Abbreviations

API, application programming Iiterface; BLAST: Basic Local Alignment Search Tool; CPU, central processing unit; FTP, file transfer protocol; GC, guanine/cytosine ratio; NCBI, National Center for Biotechnology Information (https://www.ncbi.nlm.nih.gov/); RDD: resilient distributed dataset; RefSeq: Reference Sequence.

## Competing Interests

The authors declare that they have no competing interests.

## Funding

This work was supported by EU FP7 Marie Curie Actions IAPP program through the Cloudx-i project (grant 324365) and the SageCare project (grant 644186). M.M.A. is supported by the Irish Research Council (grant EPSPD/2015/32). P.W. is funded under the Science Foundation Ireland (grant agreement 16/IFA/4342). Thanks to UNINA for hosting research.

## Author’s Contributions

B.L. conceived the project. R.L. designed and wrote initial performance tests, with contributions from B.L. B.L. wrote the manuscript with input from M.M.A. and P.W. P.W. supervised the research.

## Supplementary Material

GIGA-D-17-00268_Original_Submission.pdf

GIGA-D-17-00268_Revision_1.pdf

GIGA-D-17-00268_Revision_2.pdf

Response_to_Reviewer_Comments_Original_Submission.pdf

Response_to_Reviewer_Comments_Revision_1.pdf

Reviewer_1_Report_(Original_Submission) -- Zhaohui Qin 11/5/2017 Reviewed

Reviewer_1_Report_(Revision_1) -- Zhaohui Qin 3/3/2018 Reviewed

Reviewer_2_Report_(Original_Submission) -- Szymon Chojnacki, Ph.D. 11/17/2017 Reviewed

Reviewer_2_Report_(Original_Submission)_Attachment_giga-science.pdf

Supplemental material
