# Supplementary material for: Field of genes: using Apache Kafka as a bioinformatic data repository
Source: Gigascience. 2018 Apr 9;7(4):giy036. doi: 10.1093/gigascience/giy036 (PMC5906921; doi:10.1093/gigascience/giy036)

GigaScience

Field of Genes: Using Apache Kafka as a Bioinformatic Data Repository

--Manuscript Draft--

**Manuscript Number:**	GIGA-D-17-00268
**Full Title:**	Field of Genes: Using Apache Kafka as a Bioinformatic Data Repository
**Article Type:**	Research
**Funding Information:**	FP7 People: Marie-Curie Actions (324365) — Prof Paul Walsh<br>Irish Research Council (IE) (EPSPD/2015/32) — Dr Micheál Mac Aogáin
**Abstract:**	Background: Bioinformatic research is increasingly dependent on large-scale data sets, accessed either from private or public repositories. An example of a public repository is NCBI's RefSeq. These repositories must decide in what form to make their data available. Unstructured data can be put to almost any use, but are limited in how access to them can be scaled. Highly structured data offer improved performance for specific algorithms but limit the wider usefulness of the data. We present an alternative: lightly-structured data stored in Apache Kafka in a way that is amenable to parallel access and streamed processing, including subsequent transformations into more highly-structured representations. We contend that this approach could provide a flexible and powerful nexus of bioinformatic data, bridging the gap between low-structure on one hand, aad high-performance and scale on the other. To demonstrate this, we present a proof of concept version of NCBI's RefSeq database using this technology. We measure the performance and scalability characteristics of this alternative with respect to flat files.<br>Results: The proof of concept scales almost linearly as more compute nodes are added, outperforming the standard approach using files.<br>Conclusions: Apache Kafka merits consideration as a fast and more scalable but general-purpose way to store and retrieve bioinformatic data, for public, centralized reference datasets like RefSeq, and private clinical and experimental data.
**Corresponding Author:**	Brendan Lawlor<br>Cork Institute of Technology<br>Cork, Cork IRELAND
**Corresponding Author Secondary Information:**	
**Corresponding Author's Institution:**	Cork Institute of Technology
**Corresponding Author's Secondary Institution:**	
**First Author:**	Brendan Lawlor
**First Author Secondary Information:**	
**Order of Authors:**	Brendan Lawlor<br>Richard Lynch<br>Micheál Mac Aogáin<br>Paul Walsh
**Order of Authors Secondary Information:**	
**Opposed Reviewers:**	
**Additional Information:**	
**Question**	**Response**
Are you submitting this manuscript to a special series or article collection?	No

Powered by Editorial Manager® and ProduXion Manager® from Aries Systems Corporation

**Experimental design and statistics**<br><br>Full details of the experimental design and statistical methods used should be given in the Methods section, as detailed in our Minimum Standards Reporting Checklist. Information essential to interpreting the data presented should be made available in the figure legends.<br><br>Have you included all the information requested in your manuscript?	No
If not, please give reasons for any omissions below.<br><br>as follow-up to "**Experimental design and statistics**<br><br>Full details of the experimental design and statistical methods used should be given in the Methods section, as detailed in our Minimum Standards Reporting Checklist. Information essential to interpreting the data presented should be made available in the figure legends.<br><br>Have you included all the information requested in your manuscript?<br><br>"	Due to the nature of the paper, the specific data used is immaterial to the outcome. Additionally the data used was taken from RefSeq flat files by FTP as part of the experiment itself. This is likely to change over time, but again, will not affect the experiment design or conclusions.
**Resources**<br><br>A description of all resources used, including antibodies, cell lines, animals and software tools, with enough information to allow them to be uniquely identified, should be included in the Methods section. Authors are strongly encouraged to cite Research Resource Identifiers (RRIDs) for antibodies, model organisms and tools, where possible.<br><br>Have you included the information requested as detailed in our Minimum Standards Reporting Checklist?	No
If not, please give reasons for any omissions below.<br><br>as follow-up to "**Resources**	Not applicable.

A description of all resources used, including antibodies, cell lines, animals and software tools, with enough information to allow them to be uniquely identified, should be included in the Methods section. Authors are strongly encouraged to cite Research Resource Identifiers (RRIDs) for antibodies, model organisms and tools, where possible.<br><br>Have you included the information requested as detailed in our Minimum Standards Reporting Checklist?<br><br>"	
**Availability of data and materials**<br><br>All datasets and code on which the conclusions of the paper rely must be either included in your submission or deposited in publicly available repositories (where available and ethically appropriate), referencing such data using a unique identifier in the references and in the "Availability of Data and Materials" section of your manuscript.<br><br>Have you have met the above requirement as detailed in our Minimum Standards Reporting Checklist?	Yes

Compiled PDF file (from Latex using overleaf) Click here to download Manuscript field-genes-apache.pdf

OXFORD (GIGA)n SCIENCE

GigaScience, 20xx, 1–9

doi: xx.xxxx/xxxx
Manuscript in Preparation
Research

RESEARCH

Field of Genes: Using Apache Kafka as a Bioinformatic Data Repository

Brendan Lawlor[1,2,*], Richard Lynch[1], Micheál Mac Aogáin[3] and Paul Walsh[2]

[1]Dept. of Computing, Cork Institute of Technology, Cork, Ireland and [2]NSilico Life Sciences Ltd., Cork, Ireland and [3]Sir Patrick Dun Laboratory, Trinity Translational Medicine Institute, Department of Clinical Microbiology, School of Medicine, Trinity College Dublin, Dublin, Ireland

[*]brendan.lawlor@mycit.ie

Abstract

Background: Bioinformatic research is increasingly dependent on large-scale data sets, accessed either from private or public repositories. An example of a public repository is NCBI's RefSeq. These repositories must decide in what form to make their data available. Unstructured data can be put to almost any use, but are limited in how access to them can be scaled. Highly structured data offer improved performance for specific algorithms but limit the wider usefulness of the data. We present an alternative: lightly-structured data stored in Apache Kafka in a way that is amenable to parallel access and streamed processing, including subsequent transformations into more highly-structured representations. We contend that this approach could provide a flexible and powerful nexus of bioinformatic data, bridging the gap between low-structure on one hand, and high-performance and scale on the other. To demonstrate this, we present a proof of concept version of NCBI's RefSeq database using this technology. We measure the performance and scalability characteristics of this alternative with respect to flat files.
Results: The proof of concept scales almost linearly as more compute nodes are added, outperforming the standard approach using files. **Conclusions:** Apache Kafka merits consideration as a fast and more scalable but general-purpose way to store and retrieve bioinformatic data, for public, centralized reference datasets like RefSeq, and private clinical and experimental data.

Key words: Apache Kafka; scalability; parallelization; big data

Background

Bioinformatic data is available from a number of authoritative organizations. One such example is the *RefSeq* database which maintains records of genomic DNA for model organisms[1]. RefSeq is maintained by the National Center for Biotechnology Information (NCBI)[1], and its website includes three mechanisms for accessing the data. Firstly, by searching for sequences using the BLAST program. Secondly, by downloading the database files used by BLAST. And thirdly, by downloading the underlying fasta files used to create the database.

BLAST (Basic Local Alignment Search Tool) is an invaluable tool for bioinformaticians who are searching for a particular genetic sequence[2]. It performs an alignment of a query sequence against a database of known sequences, returning results based on similarity. But BLAST is a search engine, not a database. If the data is presented only through this search engine, then all we can do with that data is search.

On the other hand, if we have access to the raw data (for example, using the second or third mechanism above) we can process it in any way we need in order to answer biological

1 https://www.ncbi.nlm.nih.gov/

Compiled on: October 18, 2017.
Draft manuscript prepared by the author.

Key Points

- Big Genomic datasets are generally available in formats that are either very general, but slow to access, or faster to access but too specific to a given algorithm. Taking NSBI's RefSeq, for example, it's either available via the search interface itself (fast but specific) or as flat fasta files which can be FTPed from NCBI.
- Apache Kafka offers a unique architecture which can be harnessed by bioinformatic organizations to make data available at high speed, in a flexible manner, without commiting too early to an algoritm-specific structure.
- Kafka is persistent - it stores its data in a distributed and replicated fashion on continguous disk space using an append-only log data abstraction. But it is also fluid - data can be continuously updated and 'compacted', keep the data 'live'.
- We measure the speed and scalabilty of Apache Kafka in relation to flat fasta file access from RefSeq, to give a sense of the gains that can be made thanks to this optimized structure.
- Given these characteristics, and its excellent integration with complementary tools like Spark, NiFi, Hadoop and others, we propose Apache Kafka as a 'data nexus' that bridges the gap between low-structure/low-speed and high-structure/high-speed solutions. We feel this is suitable to larger organizations to store either public or private genomic databases for general-purpose processing, or easier transformation to more specialized data formats.

questions. NCBI provides a means to retrieve the underlying raw data by accessing the anonymous FTP server[2], navigating to the database folder of their choice, and downloading the contents. In the case of RefSeq, this means downloading 217 files[3] of approximately 1GB each. Each file must be unzipped and untarred in order to reveal a number of BLAST-specific binary files. Those binaries can then be converted into text *fasta* files by means of a linux-based BLAST commandline tool supplied by NCBI[4]. Each 1GB download expands to around 4GB when converted to fasta format, so the result of downloading RefSeq is almost 1TB of data spread across a few hundred fasta files, stored on a local drive.

This structure limits the usefulness of the data, in particular by hindering parallelization: If we wish to process every sequence in a group of fasta files *exactly once*, our parallelization factor is limited to the number of files, as each file must be read from start to finish by one process or thread. In this paper, we measure these structure-based limits and present an alternative, using Apache Kafka, to overcome them.

The Structure of Data

Data may be presented in many different ways for different users, depending on who they are and what they want to do with the data. This structure constrains the way in which such data may reasonably be used.

One of the characterizing qualities of bioinformatic data is that it is vast and growing. From an engineering perspective, the only rational way of processing this data is in a parallel fashion and so our data should be structured in a way that facilitates this.

What are the properties of such a structure? We propose the following non-exhaustive list of such properties:

Distribution
In order to free the data of hardware bottlenecks like network adaptors, CPUs and memory, parallel data should be distributed across multiple machines. This is analogous to exposing the largest possible working surface area of data, in contrast to the limiting effect of storing all data on one physical server. Or to use a biological metaphor, when data is not spread out over a network, it is like a gene whose DNA is supercoiled around its histones and so cannot be expressed.

Reliability
It is a property of distributed systems to be both more and less reliable, in the sense that they no longer have a single point of failure (more reliable), but on the other hand there are more elements that can break (less reliable). Distributed data's structure should protect it from this latter effect, while promoting the former.

Streaming
The advantage that streaming brings is that consumers of a stream do not have to wait until all data is downloaded before beginning to process that data. An everyday example of this is Netflix[5] - the Internet movies streaming service. In contrast with previous models of download-and-view, where the movie must be downloaded entirely before viewing can begin, with Netflix it takes 90 minutes to watch a 90-minute movie. The streaming advantage is particularly relevant for biological data which is very often processed in *pipelines*, that is, serialized stages of processing where the output of one stage acts as the input to the next. Although not all bioinformatic processing can be performed on partial data, much of it can, and by including this element of streaming, we allow for an extra dimension of parallelization when processing genomic data.

Apache Kafka

Apache Kafka[6] is not commonly considered to be a database. It is generally viewed as a message broker - an intermediary program that transfers messages (general-purpose units of information) asynchronously from one program to another via a queue or *topic*, and in this capacity, it has been identified in previous research as a suitable technology for bioinformatic applications[3]. However Kafka's developers at *LinkedIn*[7] implementated it with a wider scope of usage in mind, including "source-of-truth data storage"[4]. Kafka's *topics* are implemented as (Distributed) *Transaction Logs* - an abstract data type to which new data is only ever appended, never overwritten. Moreover, Kafka topics can be configured never to expire, and this means that the same data can be read over and over again. Topics are stored in contiguous disk space, much like files, conferring important speed advantages. But a single topic may be traversed by any number of co-operating readers in paral-

2 ftp://ftp.ncbi.nlm.nih.gov/
3 At the date of writing. This number rises over time.
4 https://www.ncbi.nlm.nih.gov/books/NBK279690/
5 Netflix is an online TV and movie streaming service. See http://www.netflix.com
6 http://kafka.apache.org/
7 https://en.wikipedia.org/wiki/LinkedIn

lel. These features combine to allow Kafka to operate as a data repository with extremely high read and write speeds.

In the following paragraphs, we describe some of the features of Kafka and explain how they confer the parallel properties we seek.

Consumers and Producers

A Kafka installation is comprised of a cluster of Kafka *brokers* on which independent *producers* (writers) and *consumers* (readers) operate. The same piece of software can be both a consumer and a producer, and we will comment further on the importance of this fact later in this paper.

Partitions

Topics are normally single entities in message brokers, but in Kafka they are divided in *partitions*, as shown in figure 1. It is these partitions that make Kafka parallel, reliable and distributed. The partitions for a given topic are spread across the cluster, providing distribution. The partition is also the unit of parallelization. If a topic is configured to have N partitions, then N consumers can read independently and in parallel - but in concert - from the same topic. Finally, topics can also be configured to have a *replication factor*, R. This is the number of copies of a partition maintained across the cluster, so that if up to $R - 1$ machines in the cluster fail, no data is lost. Incidentally, partitions also confer scalability: Topics can become arbitrarily large - holding more data than any given machine in the cluster can permit.

Consumer Groups

Another important feature of Kafka is the concept of a*consumer group*. Figure 2 shows a small cluster with two servers and a single topic broken into four partitions. Kafka allocates one partition each to the four consumers of Consumer Group B, but in Consumer Group A, where there are only two consumers, each consumer is given two partitions to read. This organisation allows a group of consumers to collaborate in order to 'drain' a topic in parallel.

Another important thing to note here is that any given partition is only read by one consumer at a time, and this means that order of reading is preserved for any given partition. Because order is preserved, we can know when a partition has been completed by adding a 'back marker' at the end of each partition. This adds another useful element to Kafka-as-a-data-store: We can do an exhaustive sweep of a topic and know when we have touched everything.

Producers and Message Keys

In contrast to consumers which are dedicated to one or more partitions, producers can insert data into any partition of a topic. Every message is composed of an optional key, and a value. Producers use a partitioning strategy to select a destination partition based on the key of the message, or choose a random partition where no key is present. This strategy can be customized by any given producer.

Log Compaction

Another important and useful aspect of message keys is their role in *log compaction*. As already mentioned earlier, messages are continuously appended to partitions and do not overwrite old values. But Kafka has a mechanism for dealing with cases where new values for old messages are sent, or where messages are deleted. This is called log compaction and works as follows: on a scheduled basis, Kafka will recopy a partition, moving from oldest to youngest message and removing any messages who have a younger version (based on key identity). Deletion of messages is brought about when the youngest message with a given key has a null value.

Thanks to this mechanism, Kafka can continue to append new messages to topics without the topic growing indefinitely. In other words, in contrast to using files, new and changed data can be made available to users without obliging them to download very large files containing very few changes. Of course where new keys are added, the amount of space needed will increase over time, and Kafka caters for this by allowing new brokers to be added to the cluster, and then rebalancing the load. This aspect of Kafka entails a certain level of complexity in the management of the cluster, and this is discussed in section .

Streaming

Finally, the Kafka API which is available in the Java language, includes support for streams. More recently, Kafka has begun to support the Reactive Streams API[8], which includes the automatic management of back-pressure when chaining many streams together into a pipeline. There are many implementations of the Reactive Streams API, including a Scala-based one in the Akka framework[9].

Methods

In order to measure the performance characteristics of a Kafka-based genomic data repository with respect to flat files, and also to understand what other properties might emerge from such an implementation, we produced a proof of concept that loads up to 11% of the RefSeq database into a single Kafka topic, spread over either 8 or 12 cloud servers, depending on needs. Each message in the topic is either a full sequence, or a part thereof in the case of sequences larger than 100,000 nucleotides. In order to convey the image of the extended 'working surface area' that we seek to create, we named this proof of concept *Field of Genes*. What follows is a description of how to build the Field of Genes, and how to measure its performance and scalability. Measurements of its performance with respect to the use of flat files (the de-facto alternative) are presented in section .

Field of Genes

To facilite reproduction of our findings for independent verification, we have made extensive use of Docker[10] to deploy Field of Genes. While it is outside the scope of this paper to describe Docker in detail, we refer readers to a number of papers which propose the wider use of this technology to enhance reproducibility in Bioinformatics, [5, 6, 7, 8], an issue which we have previously highlighted[9].

What follows are the steps taken to create and populate the Field of Genes, and to measure its performance and scalability.

Computational Fabric on the Cloud

The data storage and computational fabric for Field of Genes is build on cloud VM servers using *Digital Ocean*[11]. We created clusters of hosts (8 and 12, depending on requirements), each with 4 CPUs, 8 GB of memory and 80GB of SSD hard drive space, using *docker machine* from a Linux command line. These 8 machines were enlisted into a *docker swarm*, so that subsequent docker commands could be run on the fabric as if it were just one host.

8 http://www.reactive-streams.org/
9 http://akka.io/
10 https://www.docker.com/
11 Digital Ocean is a cloud infrastructure provider. See https://digitalocean.com

Kafka Cluster Deployment

On this swarm of machines we rolled out an equal number of Kafka broker instances, using *docker compose*. We were able to use off-the-shelf Docker images for Kafka, so no extra coding was required. As a result, the total lines of code (LOC) for creating the basic Field of Genes infrastructure was 64. This includes the script to create the swarm and the docker-compose files to deploy Kafka.

NCBI File Upload Java Library

We then developed Java library code to download, untar, unzip and convert a single RefSeq file from NCBI. In total, there are approximately 370 lines of Java code involved, including tests.

Scala/Akka Loader Agent

Finally we used the Scala language and the Akka streams API library to create a *loader agent*. This is a small autonomous program which processes instructions from one Kafka topic, uses the Java library to download the contents of a single NCBI file and then sends the downloaded sequences to another Kafka topic. We chose Scala and Akka because they are suited to parallel and streamed programming, and we were already familiar with them. But given the wide range of programming platforms that have good Kafka integration, the agents could have been implemented in one of many other ways.

Figure 3 gives a representation of this agent. It shows a process which downloads from the NCBI FTP site, and pushes sequences to a RefSeq topic. But it also shows that Kafka topics have been used to send instructions to the agent. When choosing how to send instructions to the agents, and how to receive responses, it made sense to use the Kafka infrastructure already in place. But this design decision had interesting effects which are examined in subsection .

Scala/Akka Loader Agent Deployment

Using *docker-compose*, we deployed the loader agent using different levels of parallelization - 4, 8, 12, 16, 20 and 24 - to obtain an indicative download speed for each level, as well as to test for linearity of scalability. Note that as we raised the level of parallelization, *we similarly increased the amount of data to be processed*, by increasing the number of downloaded files. We are measuring how well the benchmark and the Field of Genes can adapt to high load. A scalable system should show a flat horizontal line for time taken as both load and parallelization are increased in tandem.

Each agent was part of the same consumer group with regards to the Loader Instructions topic which, as explained in subsection , means that the partitions of that topic were evenly allocated across the agents. We set the number of partitions of the Loader Instructions topic to the same as the level of parallelization.

The producer responsible for writing to the Loader Instructions topic used incremental numeric keys for the messages and relied on the default partitioning strategy (also explained in subsection). In this way, we could be confident that the instruction messages were spread evenly across the partitions.

These configurations combined to ensure the most efficient spread of data and computation across the cluster.

Loader Measurement and Benchmark

For each level of parallelization, we measured the time elapsed from when the first Loader Instruction message was sent, to when the last RefSeq sequence was written to its destination topic.

Our benchmark for comparison was a single server of exactly the same specifications as those used by Field of Genes, using multithreading to achieve whatever levels of parallelization the system could support. Our hypothesis is that by spreading the genomic data over a wider 'working surface area' we can attain levels of parallel access and scalability that more than compensate for any performance loss due to transmitting data between machines. Therefore this benchmark architecture - a single server where no network traffic is required and no streaming is performed - is appropriate.

The combined technologies of Docker allowed us to reproduce the same server environment and change only the software components under test. This was accomplished by creating a single instance of a Digital Ocean server, and deploying a single application that - with varying numbers of threads - downloads from NCBI using the same Java library the Field of Genes agents used. Note however that due to the limited disk space on a single server, we were not able to run the benchmark to the same levels of parallelization as with the experiment. As will be seen from the results reported in section , we still arrive at a useful comparison of absolute performance and relative scalability between the benchmark and Field of Genes. The very fact that we were limited in how parallel we could make the benchmark is an indication of the problem we are addressing and the suitability of our proposed solution. This is discussed in section .

GC Content Calculation Example

The previous subsection described how the Field of Genes is constructed and populated with RefSeq data. In this section we describe one implementation of a bioinformatically useful agent that *operates on* that data. The ratio of *cytosine* and *guanine* bases (the Cs and Gs of the genetic code) to *adenine* and *thymine* (the As and Ts), is a biologically meaningful property of any given DNA sequence[10]. Measuring this value for a large number of sequences is an example of a parallel processing problem, and therefore suitable as an initial test for the Field of Genes, and a good placeholder for more sophisticated processing.

GC Content Agent

The elements of this implementation are very similar to the Loader Agent elements of the previous subsection: We construct and deploy (using varying levels of parallelization) independent agents which consume from some topics and produce into others. Figure 4 gives an overview of this agent.

From this we can see that the output of the Loader agent has become the input of the GC Content agent, hinting at the opportunity to parallelize these tasks into a streaming pipeline, as discussed earlier. Moreover, there is another discernible opportunity here, which is the pipelining of responses and instructions. We explore some aspects of this in subsection .

GC Content Agent Deployment

As with the Loader Agent experiment, we used *docker compose* to deploy the GC Content agent with increasing levels of parallelization. In each case we set the number of GC Content Instruction partitions to be the same as the level of parallelization.

GC Content Measurement and Benchmark

The measurements taken for GC Content using Field of Genes are used to gauge if this architecture leads to improved performance and scalability. As with the Loader experiment, we compare Field of Genes to a multi-threaded, single-server solution, using a single instance of precisely the same server configuration. For the same reasons given in the section on the Loader Agent benchmark, we consider this a valuable comparison. Again, our measurements are made by increasing the parallelization factor and the amount of data to be processed,

Table 1. Download times (s)

T/A	DL_b	DL_{FoG-8}	DL_{FoG-12}
4	112	301	
8	176	301	
12	247	302	
16	323	301	302
20		302	302
24		302	302

T/A: Number of Threads (Benchmark) or Agents (Field of Genes)

Table 2. GC Content times (s)

T/A	GC_b	GC_{FoG-8}	GC_{FoG-12}	Sequences
4	283	368		$1.56 \cdot 10^5$
8	552	383		$3.1 \cdot 10^5$
12	810	380		$4.58 \cdot 10^5$
16	1,101	381	368	$6.16 \cdot 10^5$
20		506	379	$7.7 \cdot 10^5$
24		562	432	$9.27 \cdot 10^5$

T/A: Number of Threads (Benchmark) or Agents (Field of Genes)

in tandem, looking for a flat system response.

The method of measurement for Field of Genes in this case is slightly different to the Loader measurement. Streams are effectively infinite sources of data. In order to know when a stream is 'complete', we either set some 'back-markers' in each partition to indicate that the end has been reached, or we wait for the system to reach a kind of equilibrium where the output is no longer changing. The notion of equilibrium is discussed in section .

We chose this latter approach for our experimental measurements. Using Kafka's administration API, we regularly measured the size of the output topic. The stream was considered complete when its size didn't change in a defined period of time - in our case 10 seconds.

Results

Table 1 shows the download time in seconds for the Benchmark (DL_b) and Field of Genes with 8 and 12 servers in the cluster respectively (DL_{FoG-8} and DL_{FoG-12}). Each row shows the results for the same fixed number of RefSeq files and level of parallelization - threads in the case of the Benchmark and agents in the case of Field of Genes (T/A). Note that the last two rows of the Benchmark are empty as the single server did not have enough space to store 20 files or more.

When we plot these download values with parallelization factors along the X-axis and download time on the Y-axis, as shown in figure 5, we can compare how well the two options scale up. A flat horizontal line represents perfect scalability where the overall time to download does not change when extra data is added, as long as the parallelization factor increases to the same degree. In this kind of plot, the scalability can be seen to be inversely proportional to the slope of the line. The greater the slope, the less scalable the system.

The same formats of data (table 2) and plot (figure 6) are presented for the case of the GC Content processing. In this case, we see a departure between the 8-node and 12-node cluster behaviour. The limits of scalability for the smaller cluster are reached between 16 and 20 RefSeq files, for reasons discussed in section . The larger cluster however is able to extend scalability further.

Whereas the previous plots are designed to show scalability, figure 7 compares the raw performance of the Benchmark and Field of Genes systems using *sequences per second* as a metric (where sequences are strings of genetic code up to 100,000 characters long). In this format, the greater the value on the Y-axis, the more performant the system.

Discussion

Interpretation of Results

The results presented in the previous section lead us to a number of clear conclusions.

When downloading data from NCBI, the two alternatives show very different characteristics. The Benchmark solution is initially quicker to download than the Field of Genes, but that performance deteriorates rapidly as more data is added (despite additional threads being made available). The Field of Genes performance remains almost perfectly constant, and outdoes the Benchmark by the time 16 RefSeq files are downloaded. Moreover, the gap in performance at lower levels of data is due entirely to an extra step required of the Field of Genes: While the Benchmark process downloads, unzips and converts the RefSeq files from NCBI, the Field of Genes process does all this *and then writes the sequences to Kafka*.

If we subtract the time needed for this extra step, Field of Genes behaves more efficiently even for lower numbers of files. This is shown by the dotted line shown in figure 5. When we remember that the data in Kafka is available as a stream, and therefore can be accessed and processed as soon as it arrives in Kafka, we can say that this dotted line is a more valid comparison with the Benchmark when considering the performance of multi-stage processing pipelines.

The GC Content processing presents a similar picture, but two differences stand out.

Firstly, a much steeper slope (151.5 compared to 17.6) is in evidence in the case of the GC Content Benchmark when compared to the download Benchmark, indicating greater difficulties in scaling the GC Content process than the download. We can reasonably guess that this poorer scalability is due to the bottleneck of processor availability, which is tighter than the network bottleneck that prevailed during download.

Secondly, the Field of Genes scalability also reached a limit, albeit a softer one and a later one. The crucial thing to point out here is that the scalability bottleneck of Field of Genes was addressed by extending the Kafka cluster from 8 nodes to 12. *This ability to arbitrarily extend size and scalability is one of the features that makes Kafka such a suitable repository for bioinformatic data.*

Emerging Characteristics

Implementing any software system involves a certain amount of *on-the-fly* design. One can never know what the complete solution will be until the finer complexities have been encountered and dealt with in the code itself. This is what is meant by the term *Emergent Design*[11], and it is a useful exercise to look back on any implementation, including (in fact especially) proofs of concept, in order to see what else can be learned from the experiment, beyond the original hypothesis.

In the case of Field of Genes, we would like to point out two unanticipated features which we believe may be worth building on.

Firstly, we note that a system that uses Kafka to store data will also tend, for expediency, to use Kafka to store instructions. This is especially the case when large numbers of autonomous agents operating on the data need to be coordinated. An emerging feature of this tendency is the ability to pipeline not only the data, but also these instructions, so that the results from one agent might trigger the behaviour of another. As we don't wish to spend too much time predicting where this may lead, it is enough for now to point out something which every biologist knows: that from many small and simple co-operating elements, very complex and intelligent pathways may be constructed.

Secondly, another feature of stream-based programming, which we have touched on when describing measurement in section , is the idea that a process in some sense may never be finished, but instead arrives at equilibrium, at which point the most recent results may be read off a final topic. As new source data is fed upstream into such a system, it creates a ripple of computation resulting in a refreshing of the final results. While this is not the behaviour that is typically expected of software systems, in the era of big genomic data which is constantly changing, it may become accepted as a suitable paradigm. The approach has a precedent in software engineering where it is known as *eventual consistency*[12].

Potential Implications

Field of Genes presents a fast and scalable access point to raw data whose structure is independent of any particular algorithm. It is an open-ended system which can accept updates in real time and propagate those changes onwards. Given this, it would make a suitable central repository or nexus into which bioinformatic data could be sent from a variety of sources, and from which bioinformatic data could be accessed for a variety of uses.

Kafka has good integration points with many other technologies, and so is well placed to act as a parallelized and streamed data nexus. Upstream, a system like Apache NiFi[12] could be configured to load data from flat files using user-friendly graphical user interfaces. Downstream, Kafka topics can feed into databases and computation platforms as diverse as Cassandra, Hadoop, Spark and BLAST. For example, Kafka's disk-stored topics could be the data source for Spark's in-memory Resilient Distributed Datasets (RDDs).

Optionally, Field of Genes is also a suitable platform to process those data directly. The clustered servers that host Kafka can serve as a computational fabric on which to process that data. Alternatively, a separate cluster comprised of computation-only nodes could be created. The technology we used for the *agents* - Reactive Streams using Scala and Akka - were designed specifically with Kafka in mind and have been previously described by the authors as suitable for genomic applications[13]. But as stated above, many other suitably streamed and parallel platforms have good Kafka integration.

Figure 8 represents this dual approach. It shows Kafka as a central nexus of relatively unstructured data - the "source of truth". These data can be processed in place, from one Kafka topic to another, or transformed into structures more suitable for specific algorithms.

12 https://nifi.apache.org/

Further Work

We chose to put RefSeq into a single topic rather than, for example, dividing it across different topics, based on taxonomic classification of the sequences.

Similarly, while we emphasized the processing of entire sets of genomic data, we made no mention of the fact that individual messages in Kafka can be directly accessed based on a three values index: Topic, Partition and Offset.

While these were reasonable choices, further research is needed in order to arrive at optimal design decisions related to this technology.

Creating working systems from proofs of concept is rarely trivial. In the case of Field of Genes - a distributed, cloud-based approach that tends towards points of equilibrium - this is especially true. Such complex clusters require a secondary management infrastructure for updating, monitoring and scaling etc. They suffer from particular issues, which must be understood and remediated. For these reasons, further work will be required in order to implement a stable, public-facing platform based on the Field of Genes approach. Moreover such an approach would be more suited - at least initially - to larger institutions with solid software engineering capabilities and resources.

Availability of source code

Lists the following:

- Project Name: Field of Genes
- Project home page: https://github.com/blawlor/field-of-genes
- Operating system(s): Linux/Docker
- Programming language: Java and Scala
- Other requirements: Docker
- License: Apache 2.0

Declarations

Consent for publication

Not applicable.

Competing Interests

The author(s) declare that they have no competing interests'.

Funding

This work was supported by EU FP7 Marie Curie Actions IAPP program through the Cloudx-i project (grant number 324365), and the SageCare project (grant number 644186). MMA is supported the Irish Research Council (grant number EPSPD/2015/32).

Author's Contributions

BL conceived the project. RL designed and wrote initial performance tests, with contributions from BL. BL wrote the manuscript with input from MMA and PW. PW supervised the project and secured funding from grant funds.

Figure 1. Anatomy of a Kafka Topic and Partitions (from Kafka website)

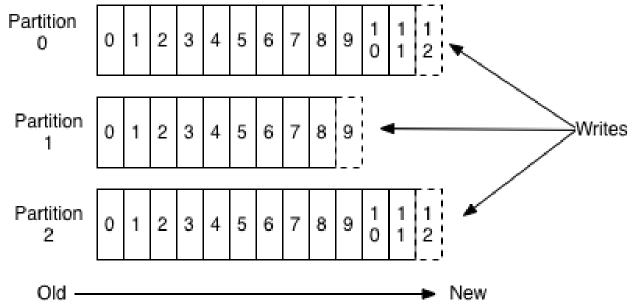

References

1. Pruitt KD, Tatusova T, Maglott DR. NCBI Reference Sequence (RefSeq): a curated non-redundant sequence database of genomes, transcripts and proteins. Nucleic acids research 2005;33(suppl 1):D501–D504.
2. Altschul SF, Gish W, Miller Wea. Basic local alignment search tool. Journal of molecular biology 1990;215(3):403–410.
3. Ta VD, Liu CM, Nkabinde GW. Big data stream computing in healthcare real-time analytics. In: Cloud Computing and Big Data Analysis (ICCCBDA), 2016 IEEE International Conference on IEEE; 2016. p. 37–42.
4. Wang G, Koshy J, Subramanian Sea. Building a replicated logging system with Apache Kafka. Proceedings of the VLDB Endowment 2015;8(12):1654–1655.
5. Moreews F, Sallou O, Bras Yea. A curated Domain centric shared Docker registry linked to the Galaxy toolshed. In: Galaxy Community Conference 2015; 2015. .
6. Belmann P, Dröge J, Bremges Aea. Bioboxes: standardised containers for interchangeable bioinformatics software. GigaScience 2015;4(1):1.
7. Moreews F, Sallou O, Ménager Hea. BioShaDock: a community driven bioinformatics shared Docker-based tools registry. F1000Research 2015;4.
8. Ali AA, El-Kalioby M, Abouelhoda M. The Case for Docker in Multicloud Enabled Bioinformatics Applications. In: International Conference on Bioinformatics and Biomedical Engineering Springer; 2016. p. 587–601.
9. Lawlor B, Walsh P. Engineering bioinformatics: building reliability, performance and productivity into bioinformatics software. Bioengineered 2015;6(4):193–203.
10. Sueoka N. On the genetic basis of variation and heterogeneity of DNA base composition. Proceedings of the National Academy of Sciences 1962;48(4):582–592.
11. Bain S. Emergent design: the evolutionary nature of professional software development. Pearson Education; 2008.
12. Bailis P, Ghodsi A. Eventual consistency today: Limitations, extensions, and beyond. Communications of the ACM 2013;56(5):55–63.
13. Lawlor B, Walsh P. The Weekend Warrior: How to Build a Genomic Supercomputer in Your Spare Time Using Streams and Actors in Scala. In: Ubiquitous Intelligence & Computing, Advanced and Trusted Computing, Scalable Computing and Communications, Cloud and Big Data Computing, Internet of People, and Smart World Congress (UIC/ATC/ScalCom/CBDCom/IoP/SmartWorld), 2016 Intl IEEE Conferences IEEE; 2016. p. 575–580.

Figure 2. Consumer Groups (from Kafka website)

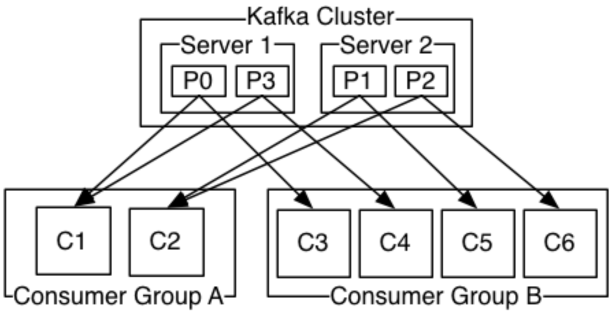

Figure 3. NCBI Download agent

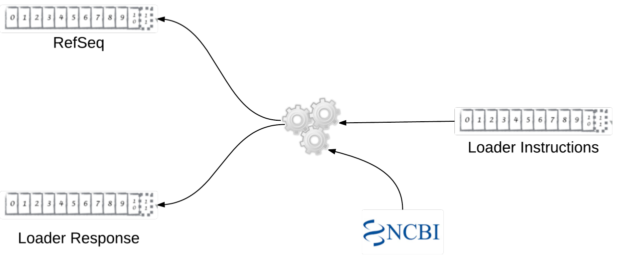

Figure 4. GC Content agent

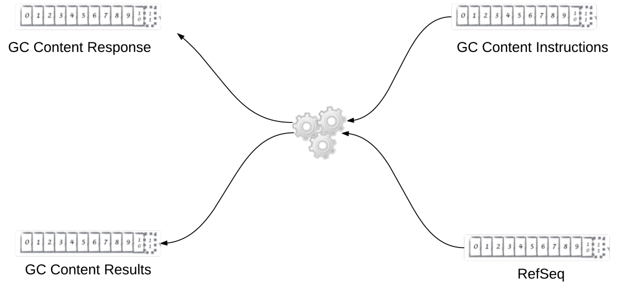

Figure 5. Scalability of Download

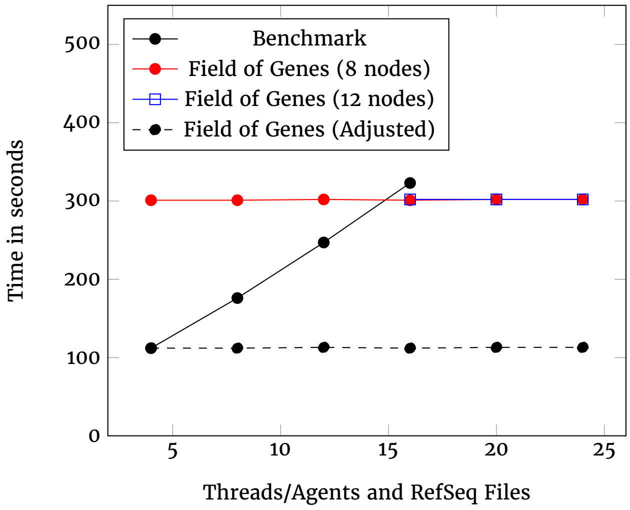

Figure 6. Scalability of GC Content

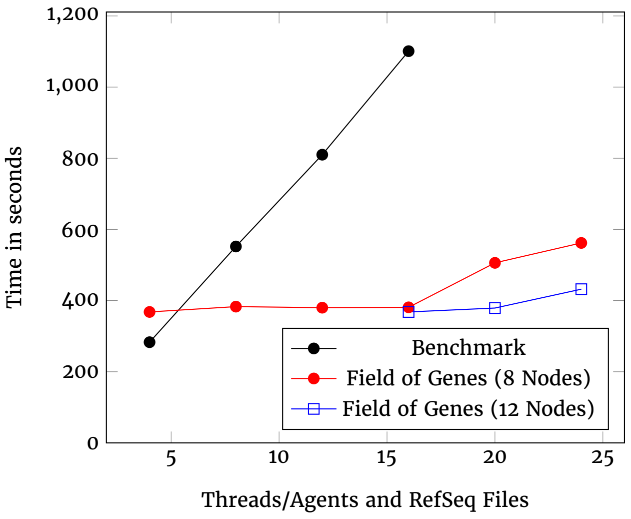

Figure 7. Processing Rates of GC Content (seq/sec)

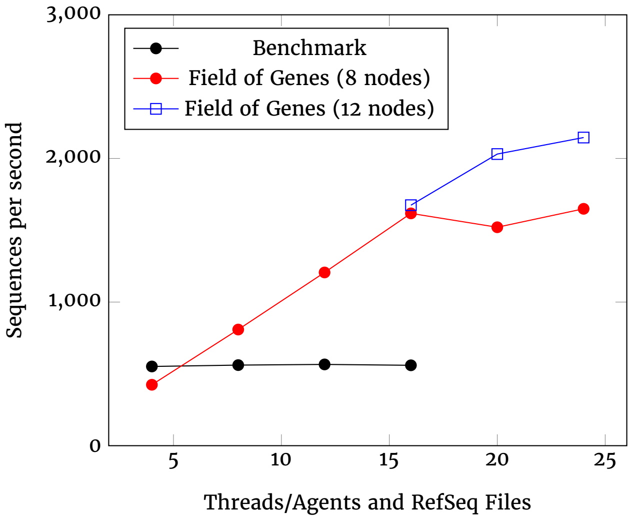

Figure 8. Example use context of Apache Kafka.

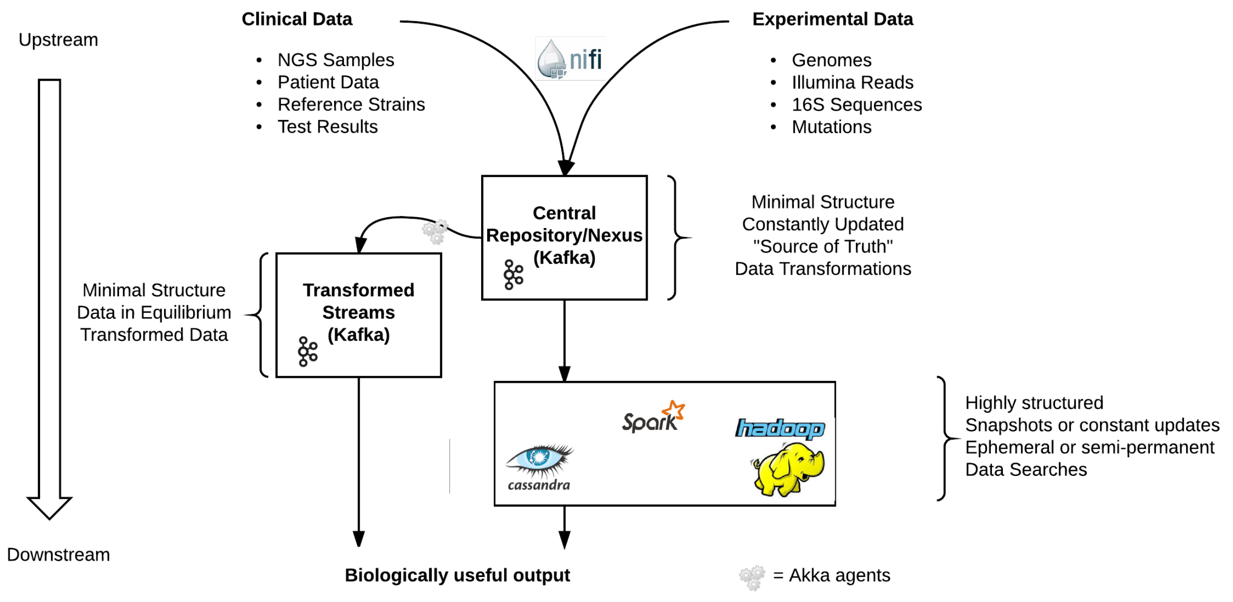

Click here to download Figure log_anatomy.png

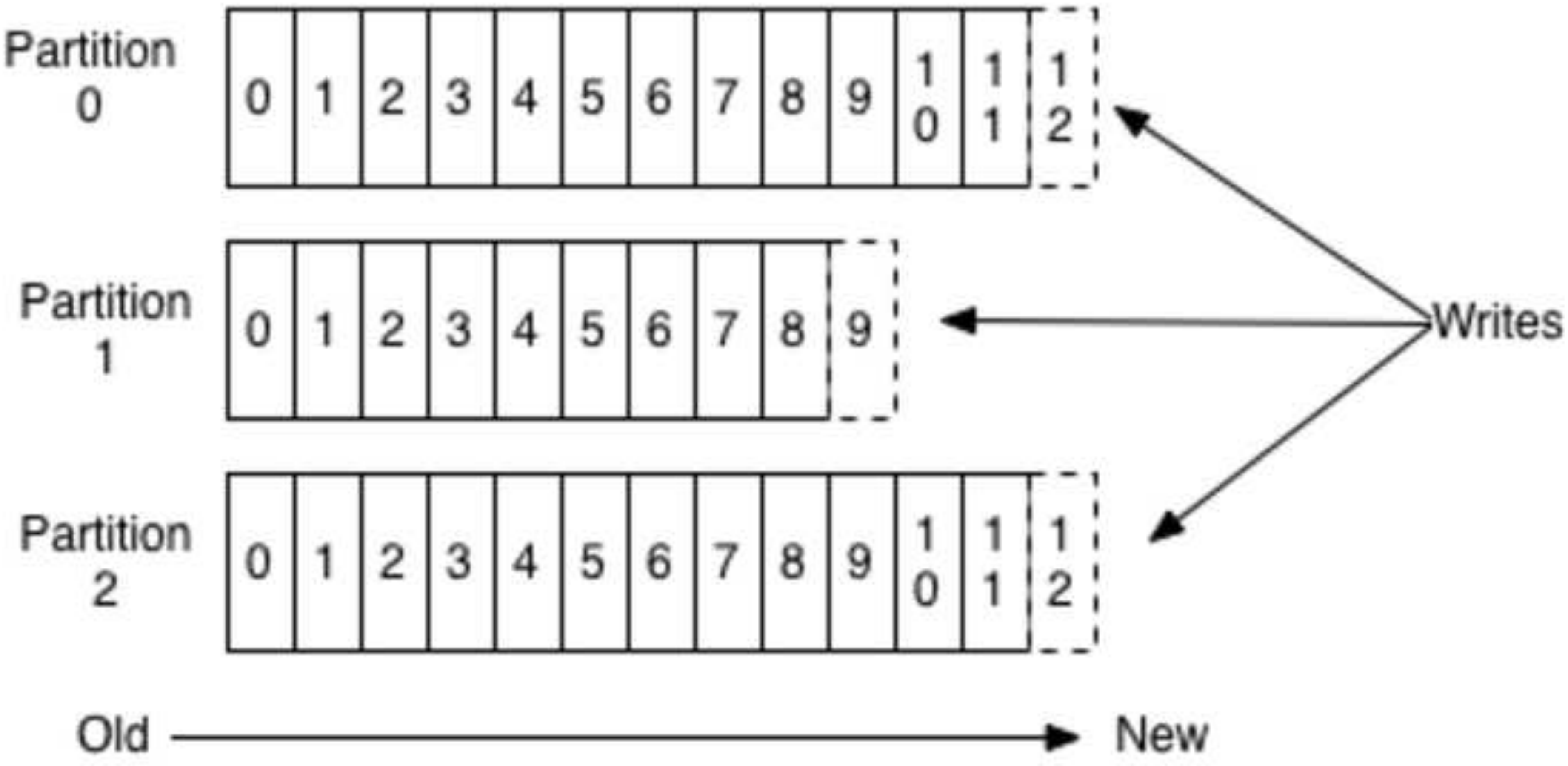

Figure 2

Click here to download Figure consumer_groups.png

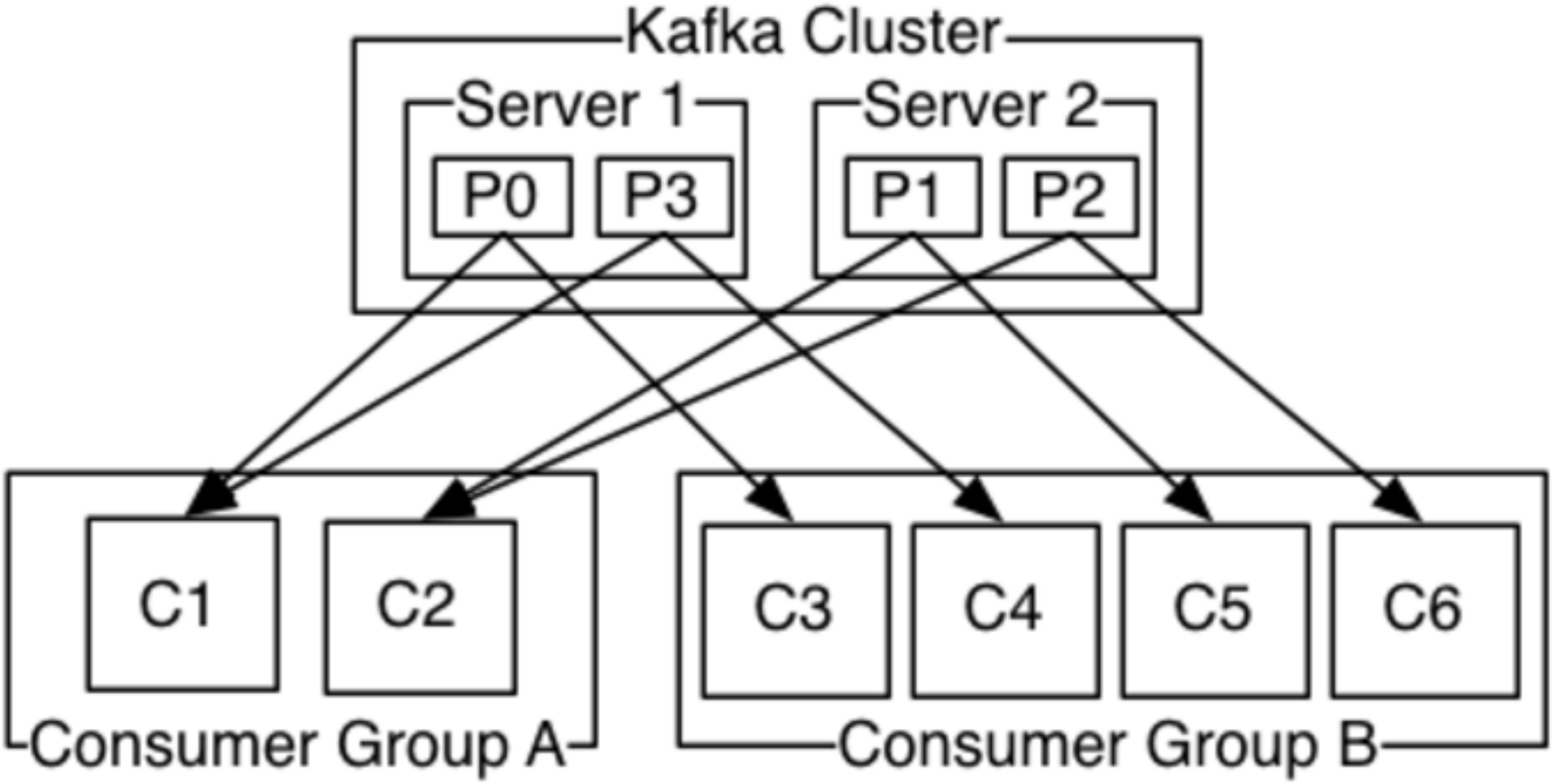

Figure 3

Click here to download Figure loader_agent.png

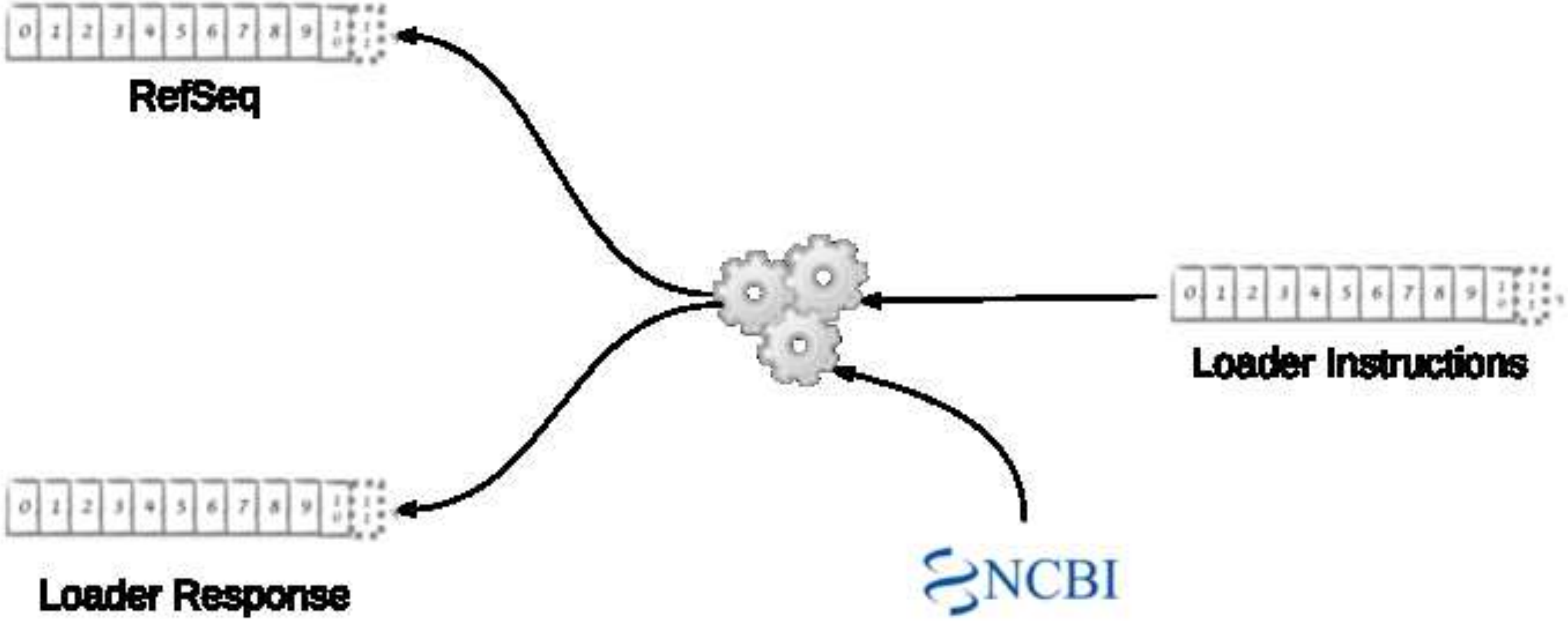

Figure 4

Click here to download Figure gccontent_agent.png

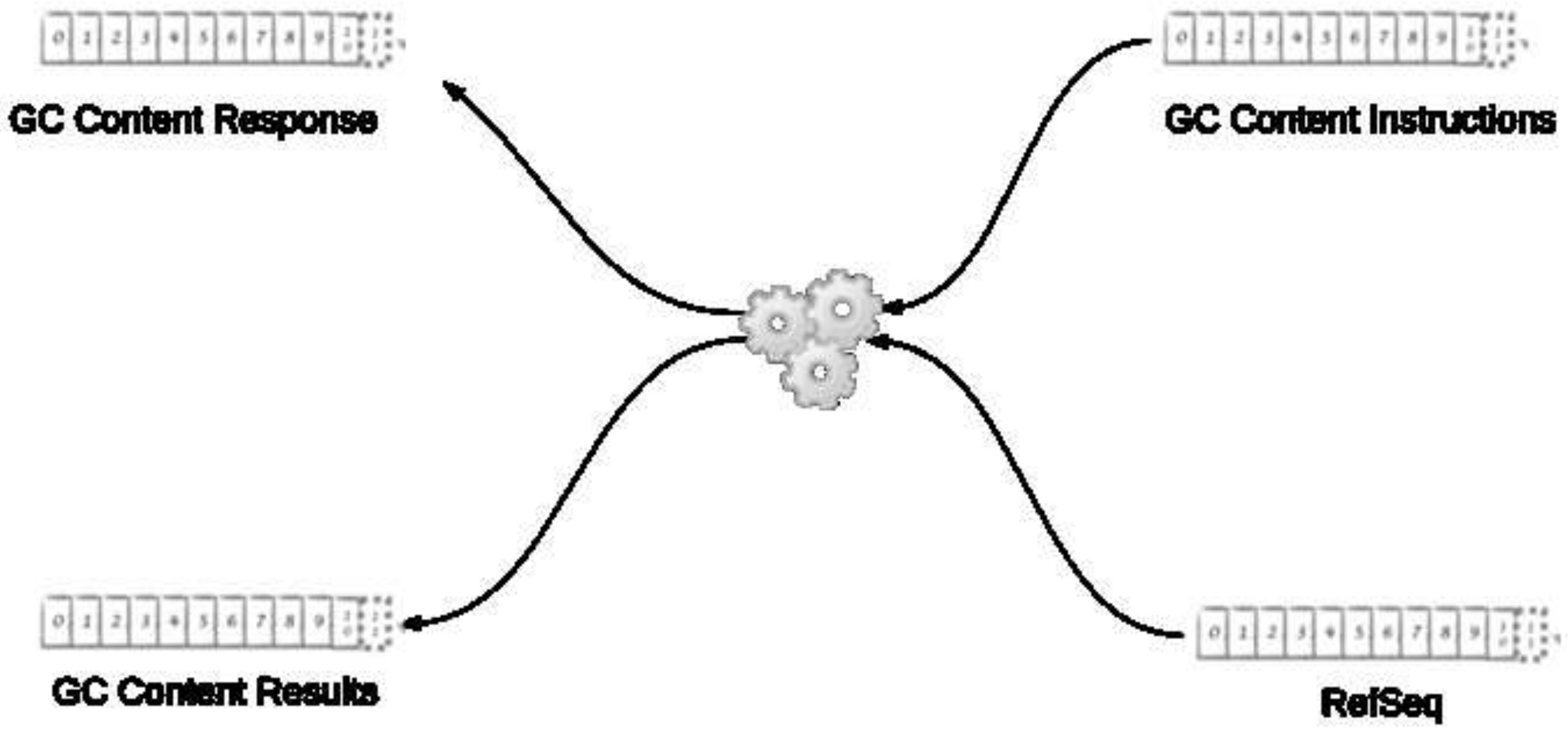

Figure 8

Click here to download Figure context.png

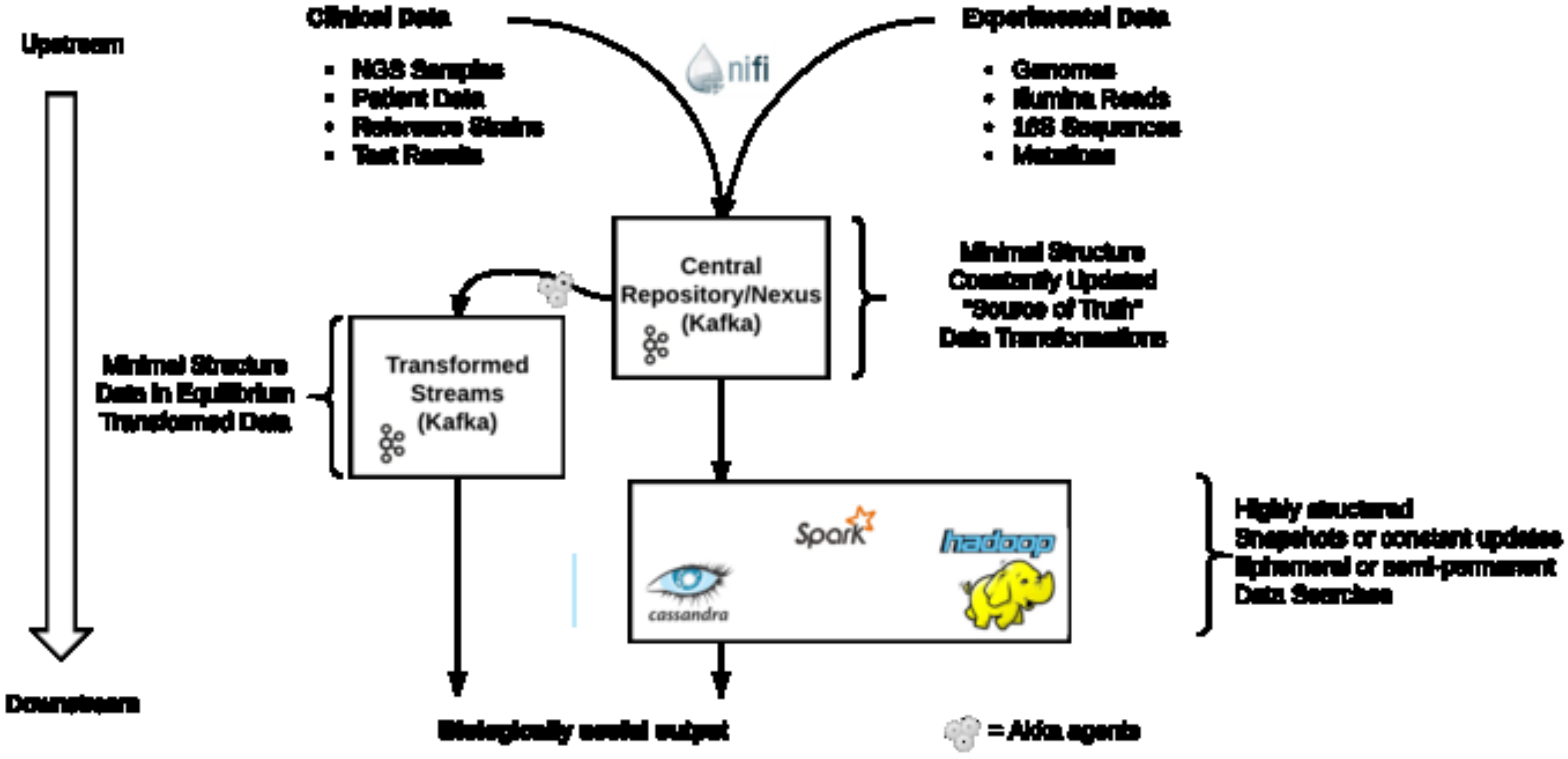

Supplement: GIGA-D-17-00268_Original_Submission.pdf [file giy036_giga-d-17-00268_original_submission.pdf]
