# Supplementary material for: Field of genes: using Apache Kafka as a bioinformatic data repository
Source: Gigascience. 2018 Apr 9;7(4):giy036. doi: 10.1093/gigascience/giy036 (PMC5906921; doi:10.1093/gigascience/giy036)

GigaScience

Field of Genes: Using Apache Kafka as a Bioinformatic Data Repository

--Manuscript Draft--

**Manuscript Number:**	GIGA-D-17-00268R1
**Full Title:**	Field of Genes: Using Apache Kafka as a Bioinformatic Data Repository
**Article Type:**	Research
**Funding Information:**	FP7 People: Marie-Curie Actions (324365) — Prof Paul Walsh<br>Irish Research Council (IE) (EPSPD/2015/32) — Dr Micheál Mac Aogáin
**Abstract:**	Background: Bioinformatic research is increasingly dependent on large-scale data sets, accessed either from private or public repositories. An example of a public repository is NCBI's RefSeq. These repositories must decide in what form to make their data available. Unstructured data can be put to almost any use, but are limited in how access to them can be scaled. Highly structured data offer improved performance for specific algorithms but limit the wider usefulness of the data. We present an alternative: lightly-structured data stored in Apache Kafka in a way that is amenable to parallel access and streamed processing, including subsequent transformations into more highly-structured representations. We contend that this approach could provide a flexible and powerful nexus of bioinformatic data, bridging the gap between low-structure on one hand, aad high-performance and scale on the other. To demonstrate this, we present a proof of concept version of NCBI's RefSeq database using this technology. We measure the performance and scalability characteristics of this alternative with respect to flat files.<br>Results: The proof of concept scales almost linearly as more compute nodes are added, outperforming the standard approach using files.<br>Conclusions: Apache Kafka merits consideration as a fast and more scalable but general-purpose way to store and retrieve bioinformatic data, for public, centralized reference datasets like RefSeq, and private clinical and experimental data.
**Corresponding Author:**	Brendan Lawlor<br>Cork Institute of Technology<br>Cork, Cork IRELAND
**Corresponding Author Secondary Information:**	
**Corresponding Author's Institution:**	Cork Institute of Technology
**Corresponding Author's Secondary Institution:**	
**First Author:**	Brendan Lawlor
**First Author Secondary Information:**	
**Order of Authors:**	Brendan Lawlor
	Richard Lynch
	Micheál Mac Aogáin
	Paul Walsh
**Order of Authors Secondary Information:**	
**Response to Reviewers:**	Firstly, to the reviewers, our thanks for their constructive and considered responses to our paper. We hope that what follows addresses your questions and concerns.<br><br>Before getting into the details of each review comment, we would like to give an overview of the changes we have made since the initial submission.<br><br>Firstly, much of the experimental codebase has been updated, with a view to making it more readable and more reproducible. The original experiment was designed to run on

Powered by Editorial Manager® and ProduXion Manager® from Aries Systems Corporation

Docker Swarm, hosted on Digital Ocean. We have found that too much has changed in Docker Swarm to make it practicable to re-run those experiments. We therefore repurposed the code to run using Kubernetes on Google Cloud. We feel this is a more mature platform and closer to being a defacto standard for container orchestration. Secondly, since we made the submission, Kafka itself has arrived at its 1.0.0 release. We updated our code accordingly.
Finally, while the results of this updated experimental setup continue to support our hypothesis, there was one difference: On Docker Swarm/Digital Ocean, the sequence loading was more easily scaled compared to the GC content calculation. Using Kubernetes/Google Container Engine, the opposite was true. This is explained by the fact that the VMs provided by Digital Ocean were different to those provided by GCE, the latter having a greater number of virtual CPUs for the same amount of memory. So while both platforms demonstrated Kafka's ability to scale out as needed by adding more nodes, the points at which that scaling out was required, and the resource constraints which necessitated them, differed.

With these considerations in mind, we move on to the individual review comments. In each case, we respond by a) answering the question/query, b) addressing the suggested change and indicating where in the paper and/or the code we have made the change, or c) defending/explaining the original point made. In each case we will indicate with a/b/c which approach applies.

Reviewer 1:
============

1.It is a good idea to use Kafka, however, the authors should also discuss the potential limitation of using Kafka and include some remarks that compare Kafka with similar tools such as Apache Flume.

Response(b): We have addressed this in an extra subsection called Drawback and Alternatives. We also introduce 'Flafka' (Flume-Kafka integration) which describes how these two systems can be harnessed together in a way that makes best use of each system's strengths and also addresses the reviewers point (5) below, re custom code.

2.

The RefSeq example is a good one. But in order to demonstrate its general applicability, it is suggested that the authors should discuss more application scenarios other than RefSeq.

Response (a,b): The GCContent calculation on RefSeq in our experiment was chosen to serve as a placeholder for more meaningful work of the same nature: Exhaustive, parallel transformations or searches on large datasets. Based on some of our other research activities, we considered some specific potential applications:

Variant Annotation: Kafka as a storage for genetic variants with a view to predicting phenotypes e.g. disease risk in humans, antibiotic resistance in bacteria.
Biomics: Perform clustering on reads (where preservation of ordering is important)
Quality control and error correction of large genomic data sets.
Taxonomy: Calculation of Average Nucleotide Identity of query genomes against subject genomes.

In at least two of these cases we are actively considering specific research on Kafka-based implementations of these applications. The difficulty with regards to the paper lies in how to outline one or more of these potential applications in a way that firstly does not distract from our central message that Kafka has a very general applicability and secondly, does not add too much length to the paper. We have opted to give an outline of how Kafka could be harnessed to perform the OrthoANI (https://www.ncbi.nlm.nih.gov/pubmed/26585518) algorithm. We have chosen this because it shows both modes of usage (using data directly on Kafka, and also transforming data to higher-structure formats where appropriate). We give only a superficial description of such an implementation for the reasons given above.

Powered by Editorial Manager® and ProduXion Manager® from Aries Systems Corporation

3.

The authors use 10 seconds without size change as the signal of ending. There is some concern that this may not be long enough considering the potential network issue when downloading data from NCBI. 10 seconds is longer enough only for within-cluster heart-beat response.

Response (b): This is a valid observation. As part of the overall changes we have made to our experimental design (see above), we have included a secondary check to ensure that the number of items in the output topics has not changed. We use the simple expedient of a second 10-second pause. This has two main advantages: Firstly, if we feel that a total of 20 seconds is still not enough, we can easily add a third 10 second pause. Secondly, even though we are waiting for 20 (or 30) seconds before deciding that a topic has 'settled down', our precision of 10 seconds remains in place. Empirically, we can report that the original 10 seconds has been sufficient for the experiment - we know how many sequences there are in each input topic and can see that this number is met or exceeded in the output topic (exceeded sometimes because of the at-least-once processing pattern that we have used). Adding the extra check has given us an extra level of confidence that the processing is complete.

4.

How is the GC content benchmark implemented? Is it also a streaming process?

Response (a): The core of the GCContent code (whch is on github) is simply a static method that takes a String sequence and returns the GCRatio as a double. It can be invoked from any Java execution context, including as part of stream processing. This same static method is indeed invoked from both the Benchmark and Experiment code. However for the Benchmark, the GC Content is executed on a fasta file which does not easily make itself amenable to streaming. The sequences are spread across multiple lines (for historical reasons, one supposes), so we cannot avail of Java's File API for reading the file per-line.

But even if we could, we are not convinced that this would be efficient as the underlying implementation would most likely read the entire file into a memory buffer first and then present the stream-like API to make the code more 'functional' in appearance. All this is indeed central to our point: Flat files - especially formats commonly used in bioinformatics - are not the best central starting point if we wish to have parallel, distributed and streamed access.

5.

A key problem for the new tool is that unlike using simple SQL query, users need to implement a different agent for each query task. A better approach to solve the reconcile data schema requirement and improve performance and scalability might be using Hadoop to download, process and import data into Apache Phoenix which provides optimized SQL queries against the data so that no agent needs to be implemented. The authors should comment on this.

Response (a,b): We would address this question by first describing the contexts in which we envisage kafka being used. There are two principle scenarios (and we have edited the paper to make this clearer in the Potential Implications section). Only the second scenario calls for custom agents, and even here this is optional. In more detail:

The first scenario is using Kafka as an initial staging point (and potentially source-of-truth repository) of low-structure data, available to be converted to high-structure data on different platforms (including Hadoop, Cassandra, BLAST and many others not yet considered). Such high-structured data could be continuously updated as new data is appended to Kafka, and would serve as the basis for the SQL queries the reviewer mentions (on Cassandra or on Haddop with the help of Phoenix), or more specialized queries (like BLAST searches).

The second scenario is using Kafka as a platform for performing high-speed, parallel processing directly on the low-structure data.

Only in the latter case, would it be desirable (though not even necessary) to create specific agents to do this processing.
The agents described in the paper were developed as part of a demonstration of this second usage scenario, and in order to demonstrate Kafka's scalability. This scalability would apply to any stream-based consumer of kafka topics - even off-the-shelf systems such as Flume, and not just custom-build agents.

Note that, as now outlined in the updated paper, Flume-Kafka integration (informally 'Flafka') addresses the issue raised by the reviewer by allowing for Flume 'agents' to act directly on Kafka topics.

So the case we are making does not make writing custom agents obligatory. In keeping with the Field of Genes moniker, the goal of the proposed approach is to expose the data in a scalable and performant way, giving the choice of how to transform or process that data to the user.

Finally, note also that there are tools for querying streams using SQL-like syntax, and they cover Kafka (PipelineDB is such an example). In principle an index into Kafka could be created, as each item can be identified using topic, partition and offset. However this would be a matter for separate research and is outside the scope of this paper.

6.

Adding more nodes usually leads to more overheads (Such as extra work to Zookeeper) and slightly increased elapsed time. in this work, I wonder why the scalability lines of Kafka stay the same.

Response (a): The motivation for adding nodes to a cluster is to provide additional disk, memory, network and CPU resources to cope with increasing load (both in terms of amount of data and intensity of access)

Because of its architecture (in particular the choice to use append only logs stored immediately to contiguous disk) Kafka has an constant read time with respect to the amount of data.
One of the things that makes Kafka so scalable is that it pushes a lot of the work normally done centrally by message brokers back onto the client (or more precisely, the kafka libraries running on the client).
In addition, later version of Kafka (we have updated our experiments to use the recently released version 1.0.0) have to delegate less work to Zookeeper. The group offset information is now held by Kafka itself. This leads to fewer calls to Zookeeper, reducing the chances that it becomes a bottleneck in large deployments. Those calls that remain are of a simple nature - Zookeeper is in the end, merely a reliable key-value store.

The Zookeeper instances themselves are also scalable. Our experimental setup uses the suggested value of 3 Zookeeper instances, and this can comfortably serve dozens or even hundreds of brokers before needing to be scaled up to 5 or more instances.
It is possible that our experimental setup (max 12 nodes) is not sufficiently large to surface any cluster-size related overhead. However the reported use of Kafka in operations such as NetFlix would indicate that clusters of two orders of magnitude greater than ours are being successfully rolled out:
https://www.slideshare.net/ConfluentInc/kafka-at-scale-in-the-cloud

7.Is the method discussed in this paper applicable to other biomedical informatics applications in which much more streaming is required than that for genomic data?

Response(1): We are a little unsure as to exactly what the reviewer means by "biomedical informatics applications", so we preface our response by outlining our

Powered by Editorial Manager® and ProduXion Manager® from Aries Systems Corporation

understanding of the question. Given the mention of "streaming" in the review question, we presume the reviewer is referring to biomedical applications involving medical imagery (e.g. radiography) and similar. If so, we do not believe that Kafka is a suitable platform for such applications. The streams that we refer to in the paper are streams of descrete data elements (e.g. genomic reads, mutations, samples, etc), which can be treated as Kafka 'messages'. Kafka is not designed to deal with the byte-streaming required for storage and retrieval of imagery. If we have misunderstood the reviewer's question, we are happy to discuss further.

Reviewer 2:
===========

I believe the article could be even better if authors wrote more about potential difficulties in implementing Kafka in production pipelines. How much effort is needed to adjust current data-generation pipelines to this new paradigm. It seems that currently curators (human or automatic) rely on RDBMS and each database has a set of legacy scripts to dump snapshots into FASTA format. Do we need to write new scripts or we could connect directly to RDBMS and within Kafka perform SQL-like joins of topics?

1.
Page 2 ### Apache Kafka # "transfer messages asynchronously". It is partially true. However, in the recent Producer API we can specify delivery mode.

Response (b): We corrected the text to read "either asynchronously or synchronously".

2.
"transfer messages via a queue or topic".
It might be misunderstood. There are no queues per se in Kafka. There are only topic. Queuing functionality is achieved within consumer groups. Whereas each consumer group operates in publisher-subscriber model.

Response (b): We corrected the text to replace "queue or topic" with "topic". Our intention was to introduce the unfamiliar reader to the word "topic" by use a more familiar word "queue" but agree that this is potentially confusing.

3.

"partitions make Kafka parallel, reliable".
It might be misleading. Main responsibility of partitions is (as stated) to improve parallelization. Whereas replicas improve reliability.

Response (b): Agree. We have reworded the 'Partitions' subsection accordingly. Please see attached revision of paper.

4.

"N consumers can read independently".
Please refer to Kafka's specification. I do not think that number of parallel consumers is limited by number of partitions.

Response (c): Our understanding is that it is indeed the case. For a given consumer group reading a given topic, an assignment is made between the consumers in the group and the partitions of the topic, such that each partition is assigned to exactly one consumer in the group, and only the consumer that owns that partition will be able to read its data while the assignment persists. If the number of consumers in the group exceeds the number of partitions, the excess consumers will be idle.
So for a given group, the number of parallel consumers is at most the number of partitions.

From the official docs at : https://kafka.apache.org/documentation/#theconsumer

"[A] topic is divided into a set of totally ordered partitions, each of which is consumed by exactly one consumer within each subscribing consumer group at any given time."

Powered by Editorial Manager® and ProduXion Manager® from Aries Systems Corporation

In our experiments, one consumer group will be used for any given processing stage so that we know when we are done - when all messages have been processed (at least once). We believe this was the intention behind the Consumer Group abstraction, and will be the way most interactions with Kafka will be coded.

5.

"R-1 machines ... fail, no data is lost"

It would be correct if 'partitions' where used instead of 'machines'.

Response (c): What was meant by this sentence is perhaps best demonstrated using an example:
If a cluster has 5 machines/nodes and a replication factor of 3, then because each partition has a total of three replicas (1 leader and 2 followers), then the failure of any 2 nodes cannot destroy all copies of any given partition, regardless of way the replicas are distributed. We have added similar wording to the paper which we hope clarifies the point.

6.

Consumer groups # "only read by one consumer at a time" Should be "only read by one consumer within a consumer group at a time"

Response (b): Agreed. We have updated text exactly as suggested.

7.

"Kafka includes support for streams"

Do authors refer to Streams API added to Kafka in version 0.10? Btw. Java 9 contains reactive streams.

Response (a,b): In part, that is what we are referring to, but also to the wider ecosystem of streaming APIs for Kafka that are developed by other systems and produce and consume data. Because of its internal architecture, Kafka topics are particularly amenable to being presented as streaming inputs and outputs.

1. Akka Kafka Streams. This is one of the stream apis that we have used in our experimental setup, to allow Akka actors to consume Kafka data. This has the advantage of implementing the Reactive Stream api (it was previously known as Reactive Kafka): http://www.reactive-streams.org/
2. Flume contains a Kafka Source and Sink based on streams
3. Apache Spark's Streaming api has support for Kafka input and output.

We reworded the section on streaming to point out that the reactive streams we were referring to was in fact a feature of Akka's Kafka stream library. So, still specific to Kafka, but created by the Akka team for use with Akka code.

And yes - we look forward to using the Java 9 reactive streams with kafka, and seeing what open source products emerge to support Kafka streaming using native java 9 Flows.

8.

Methods
- It would ease reading if references to source directories where used in each paragraph describing code.
- I did not find description of Consul tool that is used in experiments
- Docker images that are pulled from DockerHub do not have sources linked and it is somewhat difficult to match Dockerfiles from GitHub with image names
- It seems that Java classes used to upload data in a text file to Kafka topic implements functionality already available in Kafka Connect
- Please let me know if there was a reason not to use Kafka Stream API to implement

Powered by Editorial Manager® and ProduXion Manager® from Aries Systems Corporation

the GC Content agent.
- Please describe what is the content of instructions topic

Response (a,b):
- We now refer to source code directories in paper.
- Consul is no longer part of the experimental setup - it was used by Docker Swarm. We no longer use swarm, and in any case the lastest version of Swarm does not require Consul. Consul is a distributed key/value store, not unlike Zookeeper.
- We have updated the docker hub image descriptions to point at their corresponding modules under github.
- We were not aware of Kafka Connect when we designed the experiment. Java classes were used to update Kafka as in this way we could use the same library for benchmark and experiment. In any case, as outlined at the top of this document, we have de-emphasised the importance of the upload aspect of the experimentation. We now treat it as a necessary first step to creating the topics.
- We used an alternative to the standard Kafka Stream API because of our decision to use Akka agents - a Reactive Kafka library was developed by Akka developers to connect Kafka topics with Akka actors using Reactive Streams, which we felt was a better fit for our actor-based approach.
- The instruction topic, as explained in the new README files, holds the messages that trigger the Akka actors to begin the experiment. In other words, we ended up using Akka not only for data, but also for instructions. This was an interesting emergent effect that the use of Kafka had on the design, which future implementers may find useful.

9.

Page 5
Table 1

Why there are no results for DL{FOG-12} for T/A below 12, and there are results for DL{FOG-8} for T/A below 8?

Response (a,b): Our original intention was to show that when cluster size starts to limit throughput, increasing that cluster size addressed the limit. So our experiments with 12 nodes only 'kicked in' when the throughput limit showed up. That said, the new Kubernetes-based experiments plug those gaps. We have all (possible) results for all three cluster sizes (4,8 and 12).

10.

Page 6
line 20
I think that the paragraph might suggest that Kafka is eventual consistent. Which is not the case. Cassandra database (described later in the article) is eventual consistent database.

Response (a,b): It was not our intention to describe Kafka itself - or indeed any particular tool - as 'eventually consistent' but instead to point at 'eventual consistency' as a feature of some software architectures.
In the abstract case we described, changes to data in an input topic would have a downstream 'rippling' effect on the data in any output topics, with a time delay. Input and output would only be consistent if no more data were presented at the input, and enough time were given to the output to 'catch up'.
This is in contradistinction to purely transactional systems, where input is only accepted when their effect on output data has been saved.
In order to avoid giving the wrong impression, we have opted to remove this line on eventual consistency.

11.

line 40
There are also other storage levels in Apache Spark RDDs

Powered by Editorial Manager® and ProduXion Manager® from Aries Systems Corporation

	(https://spark.apache.org/docs/2.2.0/api/java/index.html?org/apache/spark/storage/StorageLevel.html)<br><br>Response (b): Understood. We have reworded to take into account that in-memory storage is not the only option available to RDDs, but that the use-case described is mostly interesting when and RDD is in memory (thus each platform plays to its strengths).<br><br>12.<br><br>###### GitHub<br><br>Please consider improving README.md files. It is not directly clear if instructions from "fabric" should be run before instruction from root. Also it seems that last instruction from "fabric" should follow last instruction from root. Please also consider updating requirements with software name and version (e.g. Java 8+, Sbt 2.10+). It looks that users need to install also JDK and Maven to build Java project in the experiment directory. The project's target is used in: https://github.com/blawlor/field-of-genes/blob/master/experiment/ex3.sh#L8 and ex3 is used in https://github.com/blawlor/field-of-genes/blob/master/run.sh#L16.<br><br>Response (b): We have completely reworked the github structure and README files. We trust that it will make more sense. We point out that the section on compiling the code is optional, given that the Docker images are published, but nonetheless outline all dependencies we are aware of to complete the build. Please feel free to raise further issues on github itself if we have still missed something.
**Additional Information:**	
**Question**	**Response**
Are you submitting this manuscript to a special series or article collection?	No
**Experimental design and statistics**<br><br>Full details of the experimental design and statistical methods used should be given in the Methods section, as detailed in our Minimum Standards Reporting Checklist. Information essential to interpreting the data presented should be made available in the figure legends.<br><br>Have you included all the information requested in your manuscript?	No
If not, please give reasons for any omissions below.<br><br>as follow-up to "**Experimental design and statistics**<br><br>Full details of the experimental design and statistical methods used should be given in the Methods section, as detailed in our Minimum Standards Reporting Checklist. Information essential to interpreting the	Due to the nature of the paper, the specific data used is immaterial to the outcome. Additionally the data used was taken from RefSeq flat files by FTP as part of the experiment itself. This is likely to change over time, but again, will not affect the experiment design or conclusions.

Powered by Editorial Manager® and ProduXion Manager® from Aries Systems Corporation

data presented should be made available in the figure legends.<br><br>Have you included all the information requested in your manuscript?<br><br>"	
**Resources**<br><br>A description of all resources used, including antibodies, cell lines, animals and software tools, with enough information to allow them to be uniquely identified, should be included in the Methods section. Authors are strongly encouraged to cite Research Resource Identifiers (RRIDs) for antibodies, model organisms and tools, where possible.<br><br>Have you included the information requested as detailed in our Minimum Standards Reporting Checklist?	No
If not, please give reasons for any omissions below.<br><br>as follow-up to "**Resources**<br><br>A description of all resources used, including antibodies, cell lines, animals and software tools, with enough information to allow them to be uniquely identified, should be included in the Methods section. Authors are strongly encouraged to cite Research Resource Identifiers (RRIDs) for antibodies, model organisms and tools, where possible.<br><br>Have you included the information requested as detailed in our Minimum Standards Reporting Checklist?<br><br>"	Not applicable.
**Availability of data and materials**<br><br>All datasets and code on which the conclusions of the paper rely must be either included in your submission or deposited in publicly available repositories (where available and ethically appropriate), referencing such data using a unique identifier in the references and in the "Availability of Data and Materials" section of your manuscript.	Yes

Have you have met the above requirement as detailed in our Minimum Standards Reporting Checklist?	

Manuscript

Click here to download Manuscript main.pdf

OXFORD (GIGA)^n SCIENCE

GigaScience, 20xx, 1–10

doi: xx.xxxx/xxxx
Manuscript in Preparation
Research

RESEARCH

Field of Genes: Using Apache Kafka as a Bioinformatic Data Repository

Brendan Lawlor[1,2,*], Richard Lynch[1], Micheál Mac Aogáin[3] and Paul Walsh[2]

[1]Dept. of Computing, Cork Institute of Technology, Cork, Ireland and [2]NSilico Life Sciences Ltd., Cork, Ireland and [3]Sir Patrick Dun Laboratory, Trinity Translational Medicine Institute, Department of Clinical Microbiology, School of Medicine, Trinity College Dublin, Dublin, Ireland

*brendan.lawlor@mycit.ie

Abstract

Background: Bioinformatic research is increasingly dependent on large-scale data sets, accessed either from private or public repositories. An example of a public repository is NCBI's RefSeq. These repositories must decide in what form to make their data available. Unstructured data can be put to almost any use, but are limited in how access to them can be scaled. Highly structured data offer improved performance for specific algorithms but limit the wider usefulness of the data. We present an alternative: lightly-structured data stored in Apache Kafka in a way that is amenable to parallel access and streamed processing, including subsequent transformations into more highly-structured representations. We contend that this approach could provide a flexible and powerful nexus of bioinformatic data, bridging the gap between low-structure on one hand, and high-performance and scale on the other. To demonstrate this, we present a proof of concept version of NCBI's RefSeq database using this technology. We measure the performance and scalability characteristics of this alternative with respect to flat files.
Results: The proof of concept scales almost linearly as more compute nodes are added, outperforming the standard approach using files. **Conclusions:** Apache Kafka merits consideration as a fast and more scalable but general-purpose way to store and retrieve bioinformatic data, for public, centralized reference datasets like RefSeq, and private clinical and experimental data.

Key words: Apache Kafka; scalability; parallelization; big data

Background

Bioinformatic data is available from a number of authoritative organizations. One such example is the *RefSeq* database which maintains records of genomic DNA for model organisms[1]. RefSeq is maintained by the National Center for Biotechnology Information (NCBI)[1], and its website includes three mechanisms for accessing the data. Firstly, by searching for sequences using the BLAST program. Secondly, by downloading the database files used by BLAST. And thirdly, by downloading the underlying fasta files used to create the database.

BLAST (Basic Local Alignment Search Tool) is an invaluable tool for bioinformaticians who are searching for a particular genetic sequence[2]. It performs an alignment of a query sequence against a database of known sequences, returning results based on similarity. But BLAST is a search engine, not a database. If the data is presented only through this search engine, then all we can do with that data is search.

On the other hand, if we have access to the raw data we can process it in any way we need in order to answer biological questions. NCBI provides a means to retrieve the underlying

1 https://www.ncbi.nlm.nih.gov/

Compiled on: February 10, 2018.
Draft manuscript prepared by the author.

Key Points

- Big Genomic datasets are generally available in formats that are either very general, but slow to access, or faster to access but too specific to a given algorithm. Taking NSBI's RefSeq, for example, it's either available via the search interface itself (fast but specific) or as flat fasta files which can be FTPed from NCBI.
- Apache Kafka offers a unique architecture which can be harnessed by bioinformatic organizations to make data available at high speed, in a flexible manner, without commiting too early to an algorithm-specific structure.
- Kafka is persistent - it stores its data in a distributed and replicated fashion on continguous disk space using an append-only log data abstraction. But it is also fluid - data can be continuously updated and 'compacted', keep the data 'live'.
- We measure the speed and scalabilty of Apache Kafka in relation to flat fasta file access from RefSeq, to give a sense of the gains that can be made thanks to this optimized structure.
- Given these characteristics, and its excellent integration with complementary tools like Spark, Flume, NiFi, Hadoop and others, we propose Apache Kafka as a 'data nexus' that bridges the gap between low-structure/low-speed and high-structure/high-speed solutions. We feel this is suitable to larger organizations to store either public or private genomic databases for general-purpose processing, or easier transformation to more specialized data formats.

raw data by accessing the anonymous FTP server[2], navigating to a database folder, and downloading the contents. In the case of RefSeq, this means downloading 217 files[3] of approximately 1GB each. Each file must be unzipped and untarred in order to reveal a number of BLAST-specific binary files. Those binaries can then be converted into text *fasta* files by means of a linux-based BLAST commandline tool supplied by NCBI[4]. Each 1GB download expands to around 4GB when converted to fasta format, so the result of downloading RefSeq is almost 1TB of data spread across a few hundred fasta files, stored on a local drive.

This structure limits the usefulness of the data, in particular by hindering parallelization: If we wish to process *every* sequence in a group of fasta files, our parallelization factor is limited to the number of files, as each file must be read from start to finish by one process or thread. In this paper, we measure these structure-based limits and present an alternative, using Apache Kafka, to overcome them.

The Structure of Data

Data may be presented in many different ways for different users, depending on who they are and what they want to do with the data. This structure constrains the way in which such data may reasonably be used.

One of the characterizing qualities of bioinformatic data is that it is vast and growing. From an engineering perspective, the only rational way of processing this data is in a parallel fashion and so our data should be structured in a way that facilitates this.

What are the properties of such a structure? We propose the following non-exhaustive list of such properties:

Distribution

In order to free the data of hardware bottlenecks like network adaptors, CPUs and memory, parallel data should be distributed across multiple machines. This is analogous to exposing the largest possible working surface area of data, in contrast to the limiting effect of storing all data on one physical server. To use a biological metaphor, when data is not spread out over a network, it is like a gene whose DNA is supercoiled around its histones and so cannot be expressed.

Reliability

It is a property of distributed systems to be both more and less reliable, in the sense that they no longer have a single point of failure (more reliable), but on the other hand there are more elements that can break (less reliable). Distributed data's structure should protect it from this latter effect, while promoting the former.

Streaming

The advantage that streaming brings is that consumers of a stream do not have to wait until all data is downloaded before beginning to process that data. An everyday example of this is Netflix[5] - the Internet movies streaming service. In contrast with previous models of download-and-view, where the movie must be downloaded entirely before viewing can begin, with Netflix it takes 90 minutes to watch a 90-minute movie. The streaming advantage is particularly relevant for biological data which is very often processed in *pipelines*, that is, serialized stages of processing where the output of one stage acts as the input to the next. Although not all bioinformatic processing can be performed on partial data, much of it can, and by including this element of streaming, we allow for an extra dimension of parallelization when processing genomic data.

Apache Kafka

Apache Kafka[6] is not commonly considered to be a database. It is generally viewed as a message broker - an intermediary program that transfers messages (general-purpose units of information) either asynchronously or synchronously from one program to another via a *topic*, and in this capacity, it has been identified in previous research as a suitable technology for bioinformatic applications[3]. However Kafka's developers at *LinkedIn*[7] implementated it with a wider scope of usage in mind, including "source-of-truth data storage"[4]. Kafka's *topics* are implemented as (Distributed) *Transaction Logs* - an abstract data type to which new data is only ever appended, never overwritten. Moreover, Kafka topics can be configured never to expire, and this means that the same data can be read over and over again. Topics are stored in contiguous disk space, much like files, conferring important speed advantages. But a single topic may be traversed by a number of co-operating

2 ftp://ftp.ncbi.nlm.nih.gov/
3 At the date of writing. This number rises over time.
4 https://www.ncbi.nlm.nih.gov/books/NBK279690/
5 Netflix is an online TV and movie streaming service. See http://www.netflix.com
6 //kafka.apache.org/
7 https://en.wikipedia.org/wiki/LinkedIn

readers in parallel. These features combine to allow Kafka to operate as a data repository with extremely high read and write speeds.

In the following paragraphs, we describe some of the features of Kafka and explain how they confer the parallel properties we seek.

Consumers and Producers

A Kafka installation is comprised of a cluster of Kafka *brokers* on which independent *producers* (writers) and *consumers* (readers) operate. The same piece of software can be both a consumer and a producer, and we will comment further on the importance of this fact later in this paper.

Partitions

Topics are normally single entities in message brokers, but in Kafka they are divided in *partitions*, as shown in figure 1. The partition is the unit of parallelization. If a topic is configured to have N partitions, then N consumers can read independently and in parallel - but in concert - from the same topic. Topics can also be configured to have a *replication factor*, R. This is the number of copies of a partition maintained across the cluster, so that if up to $R - 1$ machines in the cluster fail, no data is lost. For example, if a cluster has 7 nodes, and a replication factor of 3 for all topics (that is to say each partition has 3 copies, each on a different machine) then a loss of any 2 machines ($R - 1$) cannot delete all copies of any partition. Importantly, partitions also confer scalability: Topics can become arbitrarily large - holding more data than any given machine in the cluster can permit.

Consumer Groups

Another important feature of Kafka is the concept of a*consumer group*. Figure 2 shows a small cluster with two servers and a single topic broken into four partitions. Kafka allocates one partition each to the four consumers of Consumer Group B, but in Consumer Group A, where there are only two consumers, each consumer is given two partitions to read. This organisation allows a group of consumers to collaborate in order to 'drain' a topic in parallel.

Another important thing to note here is that any given partition is only read by one consumer within a consumer group at a time, and this means that order of reading is preserved for any given partition. Because order is preserved, we can know when a partition has been completed by adding a 'back marker' at the end of each partition. This adds another useful element to Kafka-as-a-data-store: We can do an exhaustive sweep of a topic and know when we have touched everything.

Producers and Message Keys

In contrast to consumers which are dedicated to one or more partitions, producers can insert data into any partition of a topic. Every message is composed of an optional key, and a value. Producers use a partitioning strategy to select a destination partition based on the key of the message, or choose a random partition where no key is present. This strategy can be customized by any given producer.

Log Compaction

Another important and useful aspect of message keys is their role in *log compaction*. As already mentioned earlier, messages are continuously appended to partitions and do not overwrite old values. But Kafka has a mechanism for dealing with cases where new values for old messages are sent, or where messages are deleted. This is called log compaction and works as follows: on a scheduled basis, Kafka will recopy a partition, moving from oldest to youngest message and removing any messages who have a younger version (based on key identity). Deletion of messages is brought about when the youngest message with a given key has a null value.

Thanks to this mechanism, Kafka can continue to append new messages to topics without the topic growing indefinitely. In other words, in contrast to using files, new and changed data can be made available to users without obliging them to download very large files containing very few changes. Of course where new keys are added, the amount of space needed will increase over time, and Kafka caters for this by allowing new brokers to be added to the cluster, and then rebalancing the load. This aspect of Kafka entails a certain level of complexity in the management of the cluster, and this is discussed in the Discussion section.

Streaming

Finally, the Kafka API which is available in the Java language, includes support for streams. Moreover, many other frameworks and platforms, such as Flume and Spark, have developed their own stream-based Kafka integration libraries.

More recently, Kafka libraries have emerged to support the Reactive Streams API[8], which includes the automatic management of back-pressure when chaining many streams together into a pipeline. As part of our research we have used a Scala-based library from the Akka framework[9].

Methods

In order to measure the performance characteristics of a Kafka-based genomic data repository with respect to flat files, and also to understand what other properties might emerge from such an implementation, we produced a proof of concept that loads up to 11% of the RefSeq database into a single Kafka topic, spread over 4, 8 and 12 cloud servers. Each message in the topic is either a full sequence, or a part thereof in the case of sequences larger than 100,000 nucleotides. In order to convey the image of the extended 'working surface area' that we seek to create, we named this proof of concept *Field of Genes*. What follows is a description of how to build the Field of Genes, and how to measure its performance and scalability. Measurements of its performance with respect to the use of flat files (the defacto alternative) are presented in the Results section.

Field of Genes

To facilite reproduction of our findings for independent verification, we have made extensive use of Docker[10] to deploy Field of Genes. While it is outside the scope of this paper to describe Docker in detail, we refer readers to a number of papers which propose the wider use of this technology to enhance reproducibility in Bioinformatics, [5, 6, 7, 8], an issue which we have previously highlighted[9].

What follows are the steps taken to create and populate the Field of Genes, and to measure its performance and scalability. Where appropriate, we indicate the name of corresponding software modules under source control[11].

Computational Fabric on the Cloud

The data storage and computational fabric for Field of Genes is build on a Kubernetes cluster[12] using *Google Container Engine*[13].

8 http://www.reactive-streams.org/
9 http://akka.io/
10 https://www.docker.com/
11 https://github.com/blawlor/field-of-genes
12 Kubernetes is an opensource VM clustering and container orchestration platform originally developed by Google. See https://kubernetes.io/
13 See https://cloud.google.com/kubernetes-engine/

We created clusters of hosts (4, 8 and 12, depending on requirements), each with 8 vCPUs, 30 GB of memory and 750GB of SSD hard drive space, using *gcloud*[14] from a Linux command line.

Kafka Cluster Deployment

On this Kubernetes cluster of we deployed an equal number of Kafka broker instances. We were able to use off-the-shelf Docker images for Kafka, so no extra coding was required, and we used open source examples of Kubernetes instructions to manage the deployment.

NCBI File Upload Java Library

We then developed Java library code (module loader) to download, untar, unzip and convert a single RefSeq file from NCBI.

Scala/Akka Loader Agent

Finally we used the Scala language and the Akka streams API library to create a *loader agent*. This is a small autonomous program which processes instructions from one Kafka topic, uses the Java library to download the contents of a single NCBI file and then sends the downloaded sequences to another Kafka topic (loader-agent module). We chose Scala and Akka because they are suited to parallel and streamed programming. but given the wide range of programming platforms that have good Kafka integration, the agents could have been implemented in one of many other ways (for example as Flume agents or using Spark).

Figure 3 gives a representation of this agent. It shows a process which downloads from the NCBI FTP site, and pushes sequences to a RefSeq topic. But it also shows that Kafka topics have been used to send instructions to the agent. When choosing how to send instructions to the agents, and how to receive responses, it made sense to use the Kafka infrastructure already in place. But this design decision had interesting beneficial effects which are examined in the Emerging Characteristics subsection.

Scala/Akka Loader Agent Deployment

Using *Kubernetes*, we deployed the loader agent using different levels of parallelization - 4, 8, 12, 16, 20 and 24 - to obtain an indicative download speed for each level, as well as to test for linearity of scalability. Note that as we raised the level of parallelization, *we similarly increased the amount of data to be processed*, by increasing the number of downloaded files. We are measuring how well the benchmark and the Field of Genes can adapt to high load. A scalable system should show a flat horizontal line for time taken as both load and parallelization are increased in tandem.

Each agent was part of the same consumer group with regards to the Loader Instructions topic which, as explained in the Apache Kafka subsection, means that the partitions of that topic were evenly allocated across the agents. We set the number of partitions of the Loader Instructions topic to the same as the level of parallelization.

The producer responsible for writing to the Loader Instructions topic used incremental numeric keys for the messages and relied on the default partitioning strategy (also explained in the Apache Kafka subsection). In this way, we could be confident that the instruction messages were spread evenly across the partitions.

These configurations combined to ensure the most efficient spread of data and computation across the cluster.

14 https://cloud.google.com/sdk/gcloud/

Loader Measurement and Benchmark

For each level of parallelization, we measured the time elapsed from when the first Loader Instruction message was sent, to when the last RefSeq sequence was written to its destination topic.

Our benchmark for comparison was a single server of exactly the same specifications as those used by Field of Genes, using multithreading to achieve whatever levels of parallelization the system could support. Our hypothesis is that by spreading the genomic data over a wider 'working surface area' we can attain levels of parallel access and scalability that more than compensate for any performance loss due to transmitting data between machines. Therefore this benchmark architecture - a single server where no network traffic is required and no streaming is performed - is appropriate.

The Docker technology allowed us to reproduce the same server environment and change only the software components under test. This was accomplished by creating a single Docker host and deploying a single container that - with varying numbers of threads - downloads from NCBI using the same Java library the Field of Genes agents used. Note however that due to the limitation of using a single node, compared to a cluster, we were not able to run the benchmark to the same levels of parallelization as with the experiment. As will be seen from the results reported in the Results section, we still arrive at a useful comparison of absolute performance and relative scalability between the benchmark and Field of Genes. The very fact that we were limited in how parallel we could make the benchmark is an indication of the problem we are addressing and the suitability of our proposed solution. This is discussed in the Discussion section.

GC Content Calculation Example

The previous subsection described how the Field of Genes is constructed and populated with RefSeq data. In this section we describe one implementation of a bioinformatically useful agent that *operates on* that data. The ratio of *cytosine* and *guanine* bases (the Cs and Gs of the genetic code) to *adenine* and *thymine* (the As and Ts), is a biologically meaningful property of any given DNA sequence[10]. Measuring this value for a large number of sequences is an example of a parallel processing problem, and therefore suitable as an initial test for the Field of Genes, and a good placeholder for more sophisticated processing (gccontent module).

GC Content Agent

The elements of this implementation are very similar to the Loader Agent elements of the previous subsection: We construct and deploy (using varying levels of parallelization) independent agents which consume from some topics and produce into others (gccontent-agent module). Figure 4 gives an overview of this agent.

From this we can see that the output of the Loader agent has become the input of the GC Content agent, hinting at the opportunity to parallelize these tasks into a streaming pipeline, as discussed earlier. Moreover, there is another discernible opportunity here, which is the pipelining of responses and instructions. We explore some aspects of this in the Emerging Characteristics subsection.

GC Content Agent Deployment

As with the Loader Agent experiment, we used *Kubernetes* to deploy the GC Content agent with increasing levels of parallelization. In each case we set the number of GC Content Instruction partitions to be the same as the level of parallelization.

Table 1. Download times (s)

T/A	DL_b	DL_{FoG-4}	DL_{FoG-8}	DL_{FoG-12}
4	94	96	116	99
8	216	179	165	152
12	333	381	174	146
16	445	508	259	202
20			395	214
24			484	275

T/A: Number of Threads (Benchmark) or Agents (Field of Genes). DL_b: Download on benchmark. DL_{FoG-4}: Download on Field of Genes cluster, size 4 etc.

Table 2. GC Content times (s)

T/A	GC_b	GC_{FoG-4}	GC_{FoG-8}	GC_{FoG-12}	Sequences
4	207	260	260	270	$1.56 \cdot 10^5$
8	335	275	261	262	$3.1 \cdot 10^5$
12	498	326	276	249	$4.58 \cdot 10^5$
16	664	475	288	274	$6.16 \cdot 10^5$
20			315	284	$7.7 \cdot 10^5$
24			348	286	$9.27 \cdot 10^5$

T/A: Number of Threads (Benchmark) or Agents (Field of Genes). GC_b: GC content on benchmark. GC_{FoG-4}: GC content on Field of Genes cluster, size 4 etc.

GC Content Measurement and Benchmark

The measurements taken for GC Content using Field of Genes are used to gauge if this architecture leads to improved performance and scalability. As with the Loader experiment, we compare Field of Genes to a multi-threaded, single-server solution, using a single instance of precisely the same server configuration. For the same reasons given in the section on the Loader Agent benchmark, we consider this a valuable comparison. Again, our measurements are made by increasing the parallelization factor and the amount of data to be processed, in tandem, looking for a flat system response.

The method of measurement for Field of Genes in this case is slightly different to the Loader measurement. Streams are effectively infinite sources of data. In order to know when a stream is 'complete', we either set some 'back-markers' in each partition to indicate that the end has been reached, or we wait for the system to reach a kind of equilibrium where the output is no longer changing. The notion of equilibrium is discussed in section .

We chose this latter approach for our experimental measurements. Using Kafka's administration API, we regularly measured the size of the output topic. The stream was considered complete when its size didn't change in a defined period of time - in our case 2 consecutive periods of 10 seconds.

Results

Table 1 shows the download time in seconds for the Benchmark (DL_b) and Field of Genes with 8 and 12 servers in the cluster respectively (DL_{FoG-8} and DL_{FoG-12}). Each row shows the results for the same fixed number of RefSeq files and level of parallelization - threads in the case of the Benchmark and agents in the case of Field of Genes (T/A). Note that the last two rows of the Benchmark are empty as the single server did not have enough space to store 20 files or more.

When we plot these download values with parallelization factors along the X-axis and download time on the Y-axis, as shown in figure 5, we can compare how well the two options scale up. A flat horizontal line represents perfect scalability where the overall time to download does not change when extra data is added, as long as the parallelization factor increases to the same degree. In this kind of plot, the scalability can be seen to be inversely proportional to the slope of the line. The greater the slope, the less scalable the system.

The same formats of data (table 2) and plot (figure 6) are presented for the case of the GC Content processing. In this case, we see a departure between the 8-node and 12-node cluster behaviour. The limits of scalability for the 8-node cluster start to arise between 20 and 24 RefSeq files, for reasons discussed in the Discussion section. The 12-node cluster however is able to extend scalability further.

Whereas the previous plots are designed to show scalability, figure 7 compares the raw performance of the Benchmark and Field of Genes systems using *sequences per second* as a metric (where sequences are strings of genetic code up to 100,000 characters long). In this format, the greater the value on the Y-axis, the more performant the system.

Discussion

Interpretation of Results

The results presented in the previous section lead us to draw a number of clear conclusions.

When downloading data from NCBI, the two alternatives show very different characteristics. The Benchmark solution is similar to the Field of Genes with 4 nodes. The Field of Genes performance improves as the number of nodes increases. Note that while the Benchmark process downloads, unzips and converts the RefSeq files from NCBI, the Field of Genes process does all this *and then writes the sequences to Kafka*.

The GC Content processing presents a similar picture, but in this case, even the 4-node Field of Genes cluster performs better than the benchmark. This advantage becomes more pronounced as the cluster size increases. The 12-node cluster shows an effectively flat response, indicated almost perfect scalability. This ability to arbitrarily extend performance by *scaling out* (distributing across more nodes) is one of the features of Kafka that makes it such a suitable repository for bioinformatic data.

Emerging Characteristics

Implementing any software system involves a certain amount of *on-the-fly* design. One can never know what the complete solution will be until the finer complexities have been encountered and dealt with in the code itself. This is what is meant by the term *Emergent Design*[11], and it is a useful exercise to look back on any implementation, including (in fact especially) proofs of concept, in order to see what else can be learned from the experiment, beyond the original hypothesis.

In the case of Field of Genes, we would like to point out two unanticipated features which we believe may be worth building on.

Firstly, we note that a system that uses Kafka to store data will also tend, for expediency, to use Kafka to store instructions. This is especially the case when large numbers of autonomous agents operating on the data need to be coordinated. An emerging feature of this tendency is the ability to pipeline not only the data, but also these instructions, so that the results from one agent might trigger the behaviour of another. As we don't wish to spend too much time predicting where this may lead, it is enough for now to point out something which every biol-

ogist knows: that from many small and simple co-operating elements, very complex and intelligent pathways may be constructed.

Secondly, another feature of stream-based programming, which we have touched on when describing measurement in the GC Content Calculation Example subsection, is the idea that a process in some sense may never be finished, but instead arrives at equilibrium, at which point the most recent results may be read off a final topic. As new source data is fed upstream into such a system, it creates a ripple of computation resulting in a refreshing of the final results. While this is not the behaviour that is typically expected of software systems, in the era of big genomic data which is constantly changing, it may become accepted as a suitable paradigm.

Drawbacks and Alternatives

Given its characteristics, Kafka is not suitable for some applications and contexts. It is complex software which is designed to run in a cluster so it can be difficult to deploy and maintain correctly. For this reason, at least for the time being, it is suitable only for teams with specialized software engineering skills. We believe that this situation will improve over time as Kafka becomes easier to deploy in a containerized setting. We also note recent commercial offerings of "Kafka as a Service"[15], which would outsource this complexity.

There are alternative systems available whose characteristics overlap with Kafka. One such system is Apache Flume[16]. The overlap in their functionality consists in the fact that both Kafka and Flume are message brokers: They provide a channel for message producers and consumers to exchange data.

However there are significant design differences which separate them. Most importantly, Kafka is designed to be persistant and replicated, making it suitable as a primary data repository. Flume typically uses in-memory queues, which lose data in the event of a failure. While Flume more recently includes a file-based channel, this is not replicated and so is only as reliable as the disks that it uses.

The point is that there are "horses for courses". Flume should be considered primarily a platform for transporting and transforming data, especially data intended for Hadoop storage. Kafka, while also transporting general-purpose data, was designed from the outset to persist that data in a replicated fashion. We describe how these differences can be harnessed in a collaboration in the next section.

Potential Implications

Field of Genes presents a fast and scalable access point to raw data whose structure is independent of any particular algorithm. It is an open-ended system which can accept updates in real time and propagate those updates onwards. Given this, it would make a suitable central repository or nexus into which bioinformatic data could be sent from a variety of sources, and from which bioinformatic data could be accessed for a variety of uses. We envisage two kinds of usage scenarios.

The first scenario would be to use Kafka as an initial staging point (and potentially source-of-truth repository) of low-structure data, available to be converted to high-structure data of different formats, including but certainly not limited to, Cassandra and BLAST.

Kafka has good integration points with many other technologies, and so is well placed to act as a parallelized and streamed data nexus.

Upstream, a system like Apache NiFi[17] or Apache Flume, could be configured to load data into Kafka topics. Downstream, Kafka topics can feed into databases and computation platforms as diverse as Cassandra, Hadoop, Flume, Spark and BLAST. For example, Kafka's disk-stored topics could be the data source for Spark's Resilient Distributed Datasets (RDDs) - an especially interesting use case when the RDD storage level is configured to be "memory only". These high-structure platforms would serve as the basis for SQL-like queries (on Cassandra or on Haddop with the help of Phoenix), or more specialized queries (like BLAST searches). Such downstream high-structure data could be continuously updated as new data is appended to Kafka.

The contrasting features of Flume and Kafka can make them ideal partners in a single data ecosystem. Flume-Kafka integrations (informally "Flafka") have been developed to make it easier to write Flume agents to act as producers and consumers of Kafka topics[18].

The second scenario would be the use of Kafka as a platform for performing high-speed, parallel processing *directly on the low-structure data*. The *gc-content agents* described in this paper were developed in order to demonstrate this second usage scenario, and also in order to demonstrate Kafka's scalability. But this scalability and parallelization would apply to *any* stream-based consumer of kafka topics - even off-the-shelf systems such as Flume, and not just custom-built agents.

The technology we used for the *agents* - Reactive Streams using Scala and Akka - were designed specifically with Kafka in mind and have been previously described by the authors as suitable for genomic applications[12]. But as stated above, many other suitably streamed and parallel platforms have good Kafka integration, freeing users to process the Kafka topics using their preferred technologies.

Figure 8 represents this dual approach. It shows Kafka as a central nexus of relatively unstructured data - the "source of truth". These data can be processed in place, from one Kafka topic to another, or transformed into structures more suitable for specific algorithms.

Suggested Applications

The GCContent calculation on RefSeq in our experiment was chosen to serve as a placeholder for more meaningful work of the same nature: exhaustive, parallel transformations or searches on large datasets. Here we consider some specific potential applications. This list is intended to be indicative rather than comprehensive:

- *Variant annotation*: Kafka as a storage for genetic variants with a view to predicting phenotypes (e.g. disease risks in humans, antibiotic resistance in bacteria).
- *Differential Expression Analysis (DEA)*: Perform clustering on sequencing reads (preservation of ordering is important)
- *Genomics*: Quality control and error correction of large genomic data sets.
- *Taxonomy*: Calculation of Average Nucleotide Identity of query genomes against subject genomes.

To take the last of this list, the OrthoANI[13] algorithm finds Average Nucleotide Identities between query and subject genomes by breaking both query and subject into frag-

15 Examples include https://www.cloudkarafka.com/ and https://www.confluent.io/
16 https://flume.apache.org/
17 https://nifi.apache.org/
18 See https://www.linkedin.com/pulse/flafka-big-data-solution-silos-randall-shane/

ments, finding query and subject fragments with *reciprocally*best matching Nucleotide Identities, and using only these reciprocal matches to calculate the average nucleotide identity (ANI).

In a Kafka implementation, one topic each could represent the query fragments and another the subject fragments. A consumer group could read the Subject fragments topic to completion, and build a Blast database using each fragment as a database entry. It would then stream the Query fragment topic and for each query fragment, find the best match and its Nucleotide Identity, sending the resulting match (and Nucleotide Identity) to an output topic. A second consumer group would do the same thing, but opposite: reading the Query topic to completion, and streaming the Subject topic.

The two resulting output topics, from the opposite processes above, would be treated differently. One would be read to completion to create an indexed lookup table in a distributed database like Cassandra. The other would be iterated over by another consumer group, finding cases where the match is reciprocal. Reciprocal matches would be written to a final output topic from which a running average would be calculated. This running average would move to the OrthoANI for the query and subject genomes.

The above, necessarily brief example, demonstrates a number of advantages of using Kafka:

- a high degree of parallelization (set by the size of the consumer groups);
- a pipeline parallelization (each phase can begin as soon as output from the previous phase starts to arrive);
- examples of data format transformation to more structure data when required (BLAST and Cassandra in this case)
- examples of data being processed directly from the Kafka topics (the invoking of BLAST to find matches, and the calculation of running average)
- an equilibrium-based system (the calculation will tend towards the result, even before the processing is complete, which may be enough to decide whether, for example, the query and subject belong to the same species).

Further Work

We chose to put RefSeq into a single topic rather than, for example, dividing it across different topics, based on taxonomic classification of the sequences.

Similarly, while we emphasized the processing of entire sets of genomic data, we made no mention of the fact that individual messages in Kafka can be directly accessed based on a three values index: Topic, Partition and Offset.

While these were reasonable choices, further research is needed in order to arrive at optimal design decisions related to this technology.

Creating working systems from proofs of concept is rarely trivial. In the case of Field of Genes - a distributed, cloud-based approach that tends towards points of equilibrium - this is especially true. Such complex clusters require a secondary management infrastructure for updating, monitoring and scaling etc. They suffer from particular issues, which must be understood and remediated. For these reasons, further work will be required in order to implement a stable, public-facing platform based on the Field of Genes approach. Moreover such an approach would be more suited - at least initially - to larger institutions with solid software engineering capabilities and resources.

Availability of source code

Lists the following:

- Project Name: Field of Genes
- Project home page: https://github.com/blawlor/field-of-genes
- Operating system(s): Linux/Docker
- Programming language: Java and Scala
- Other requirements: Docker
- License: Apache 2.0

Declarations

Consent for publication

Not applicable.

Competing Interests

The author(s) declare that they have no competing interests’.

Funding

This work was supported by EU FP7 Marie Curie Actions IAPP program through the Cloudx-i project (grant number 324365), and the SageCare project (grant number 644186). MMA is supported the Irish Research Council (grant number EPSPD/2015/32). Paul Walsh is funded under Science Foundation Ireland grant agreement number 16/IFA/4342.

Author’s Contributions

BL conceived the project. RL designed and wrote initial performance tests, with contributions from BL. BL wrote the manuscript with input from MMA and PW. PW supervised the research.

Figure 1. Anatomy of a Kafka Topic and Partitions (from Kafka website)

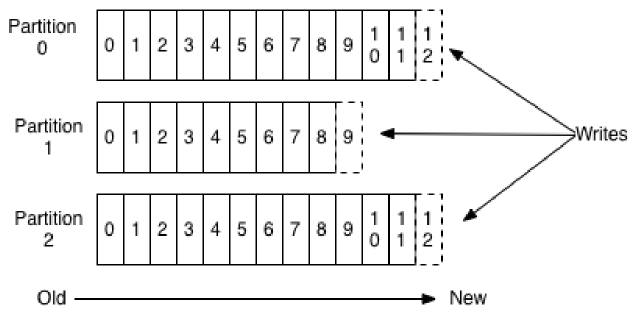

Figure 2. Consumer Groups (from Kafka website)

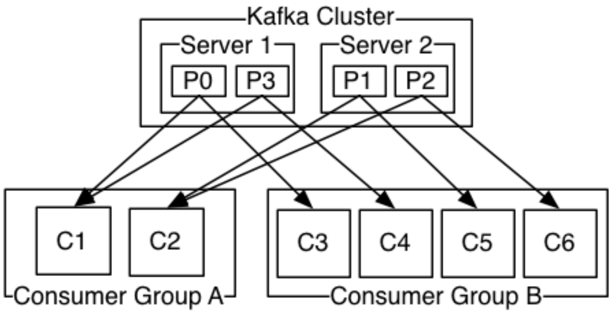

Figure 3. NCBI Download agent

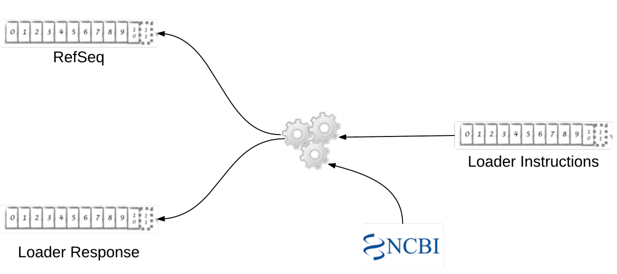

Figure 4. GC Content agent

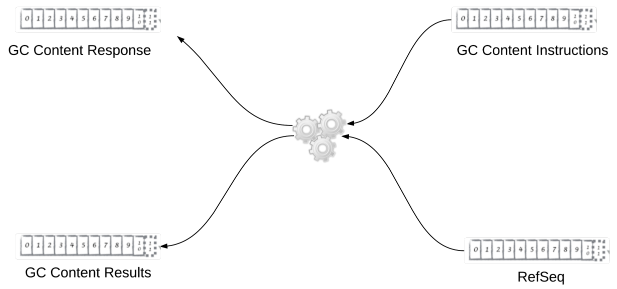

Figure 5. Scalability of Download

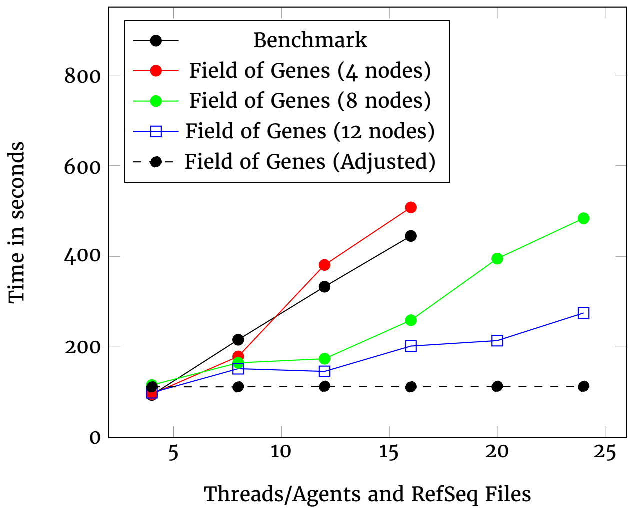

References

1. Pruitt KD, Tatusova T, Maglott DR. NCBI Reference Sequence (RefSeq): a curated non-redundant sequence database of genomes, transcripts and proteins. Nucleic acids research 2005;33(suppl 1):D501–D504.
2. Altschul SF, Gish W, Miller Wea. Basic local alignment search tool. Journal of molecular biology 1990;215(3):403–410.
3. Ta VD, Liu CM, Nkabinde GW. Big data stream computing in healthcare real-time analytics. In: Cloud Computing and Big Data Analysis (ICCCBDA), 2016 IEEE International Conference on IEEE; 2016. p. 37–42.
4. Wang G, Koshy J, Subramanian Sea. Building a replicated logging system with Apache Kafka. Proceedings of the VLDB Endowment 2015;8(12):1654–1655.
5. Moreews F, Sallou O, Bras Yea. A curated Domain centric shared Docker registry linked to the Galaxy toolshed. In: Galaxy Community Conference 2015; 2015. .
6. Belmann P, Dröge J, Bremges Aea. Bioboxes: standardised containers for interchangeable bioinformatics software. GigaScience 2015;4(1):1.
7. Moreews F, Sallou O, Ménager Hea. BioShaDock: a community driven bioinformatics shared Docker-based tools registry. F1000Research 2015;4.
8. Ali AA, El-Kalioby M, Abouelhoda M. The Case for Docker in Multicloud Enabled Bioinformatics Applications. In: International Conference on Bioinformatics and Biomedical Engineering Springer; 2016. p. 587–601.
9. Lawlor B, Walsh P. Engineering bioinformatics: building reliability, performance and productivity into bioinformatics software. Bioengineered 2015;6(4):193–203.
10. Sueoka N. On the genetic basis of variation and heterogeneity of DNA base composition. Proceedings of the National Academy of Sciences 1962;48(4):582–592.
11. Bain S. Emergent design: the evolutionary nature of professional software development. Pearson Education; 2008.
12. Lawlor B, Walsh P. The Weekend Warrior: How to Build a Genomic Supercomputer in Your Spare Time Using Streams and Actors in Scala. In: Ubiquitous Intelligence & Computing, Advanced and Trusted Computing, Scalable Computing and Communications, Cloud and Big Data Computing, Internet of People, and Smart World Congress (UIC/ATC/ScalCom/CBDCom/IoP/SmartWorld), 2016 Intl IEEE Conferences IEEE; 2016. p. 575–580.
13. Lee I, Kim YO, Park SC, Chun J. OrthoANI: an improved algorithm and software for calculating average nucleotide identity. International journal of systematic and evolutionary microbiology 2016;66(2):1100–1103.

Figure 6. Scalability of GC Content

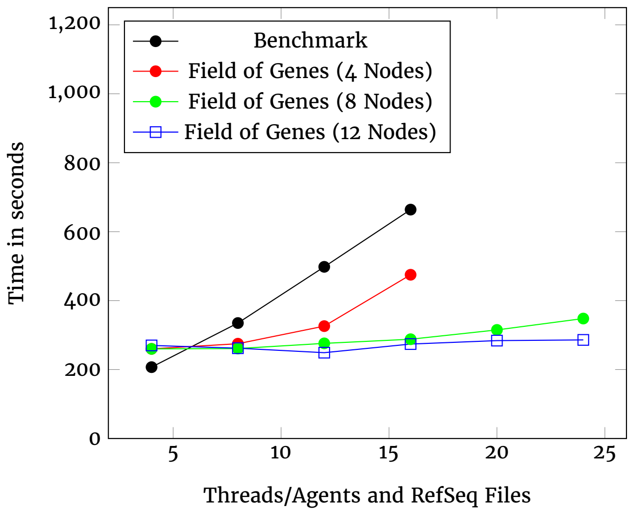

Figure 7. Processing Rates of GC Content (seq/sec)

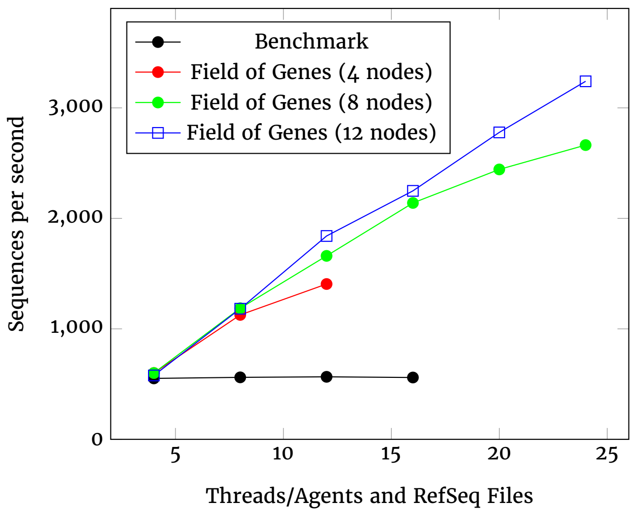

Figure 8. Example use context of Apache Kafka.

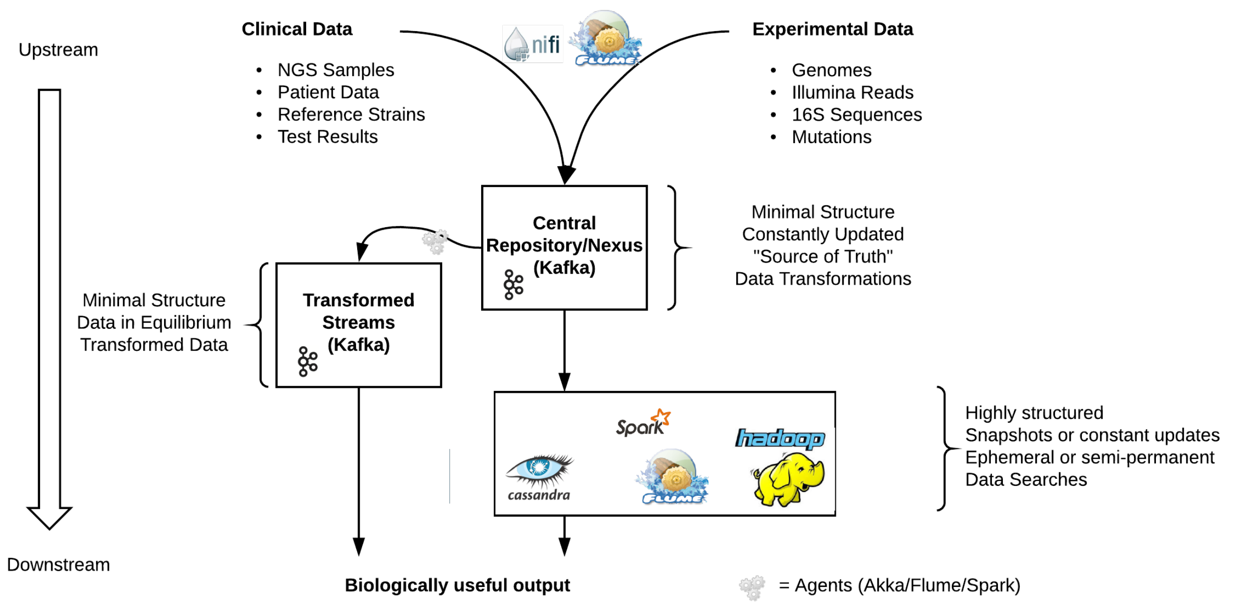

Figure 2

Click here to download Figure consumer_groups.png

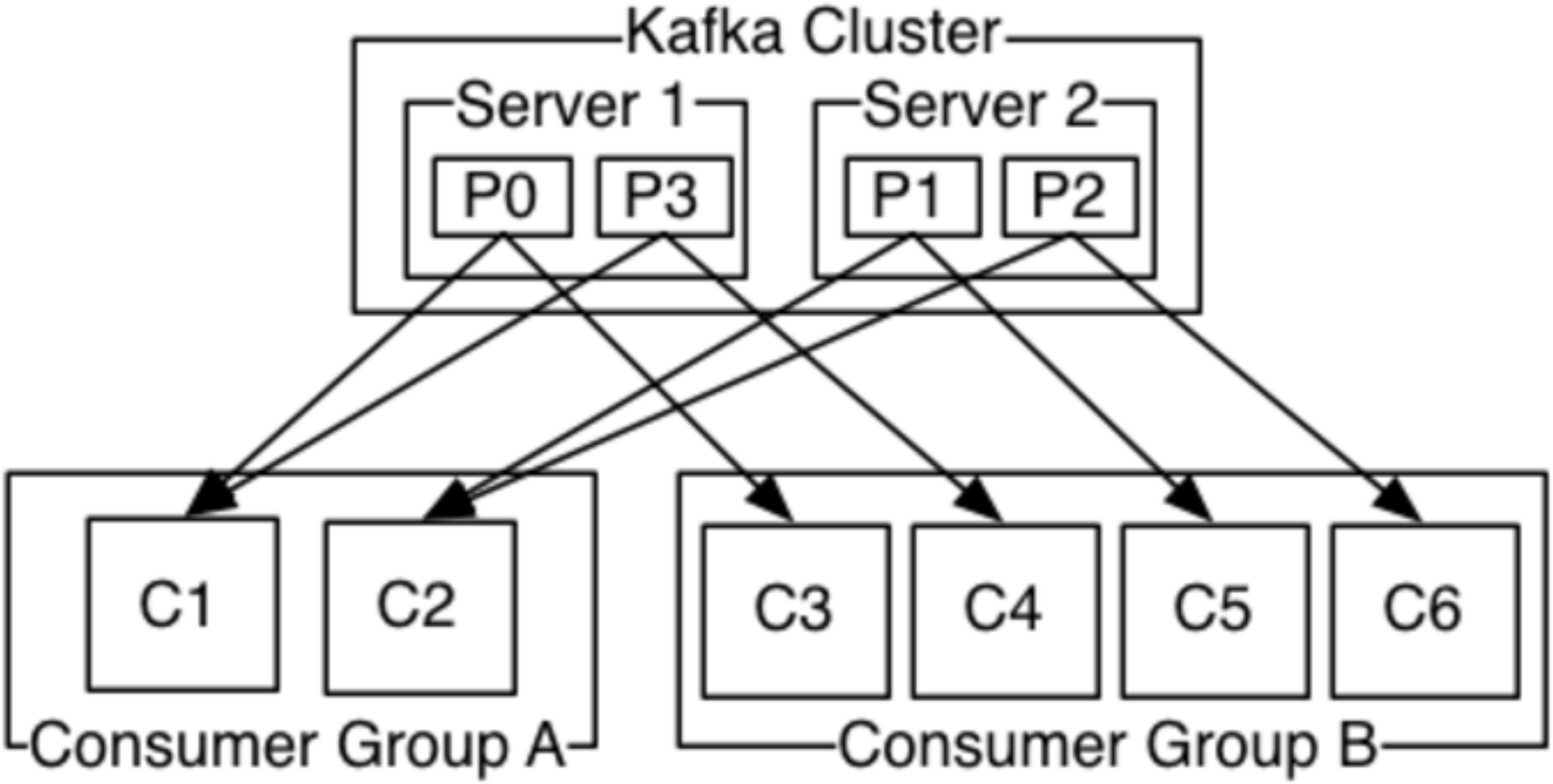

Figure 4

Click here to download Figure gccontent_agent.png

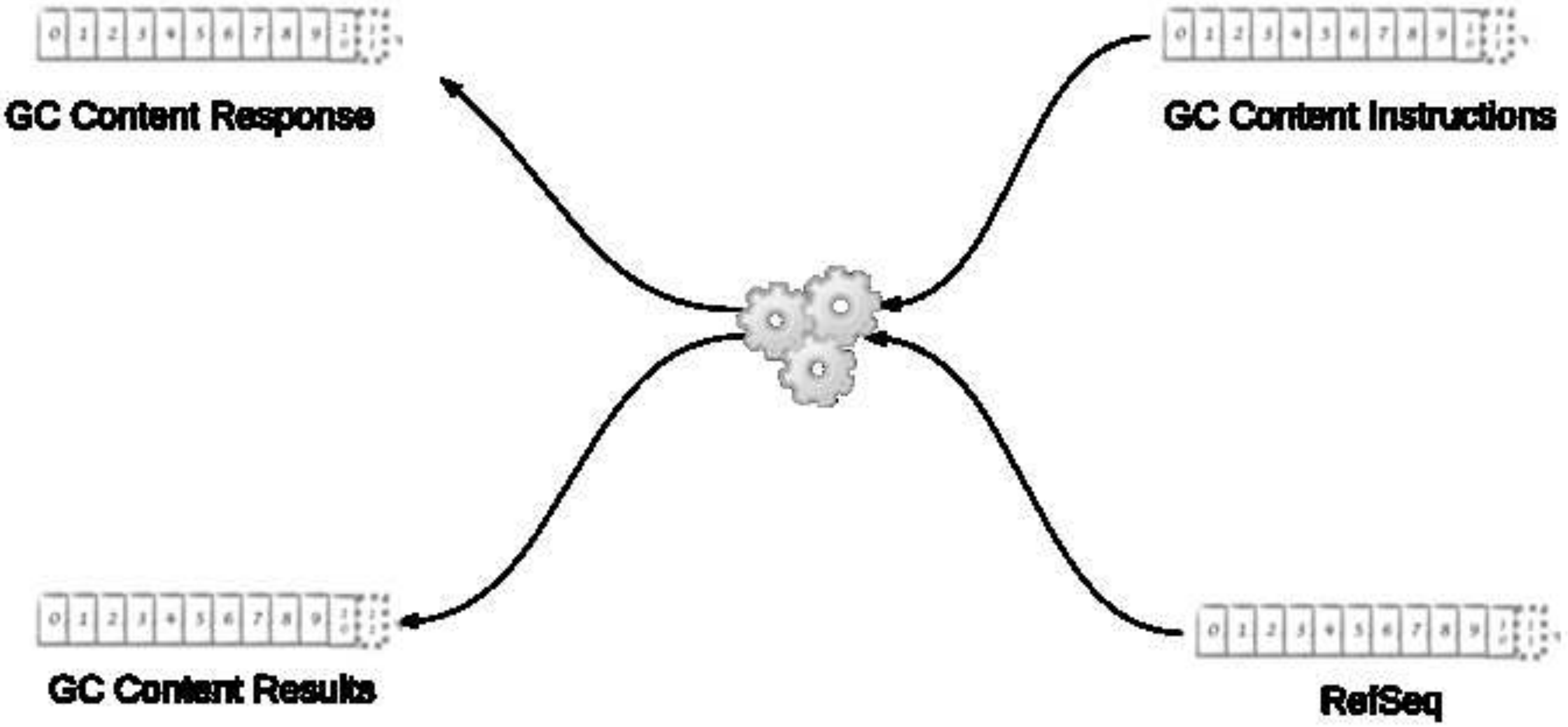

Figure 3

Click here to download Figure loader_agent.png

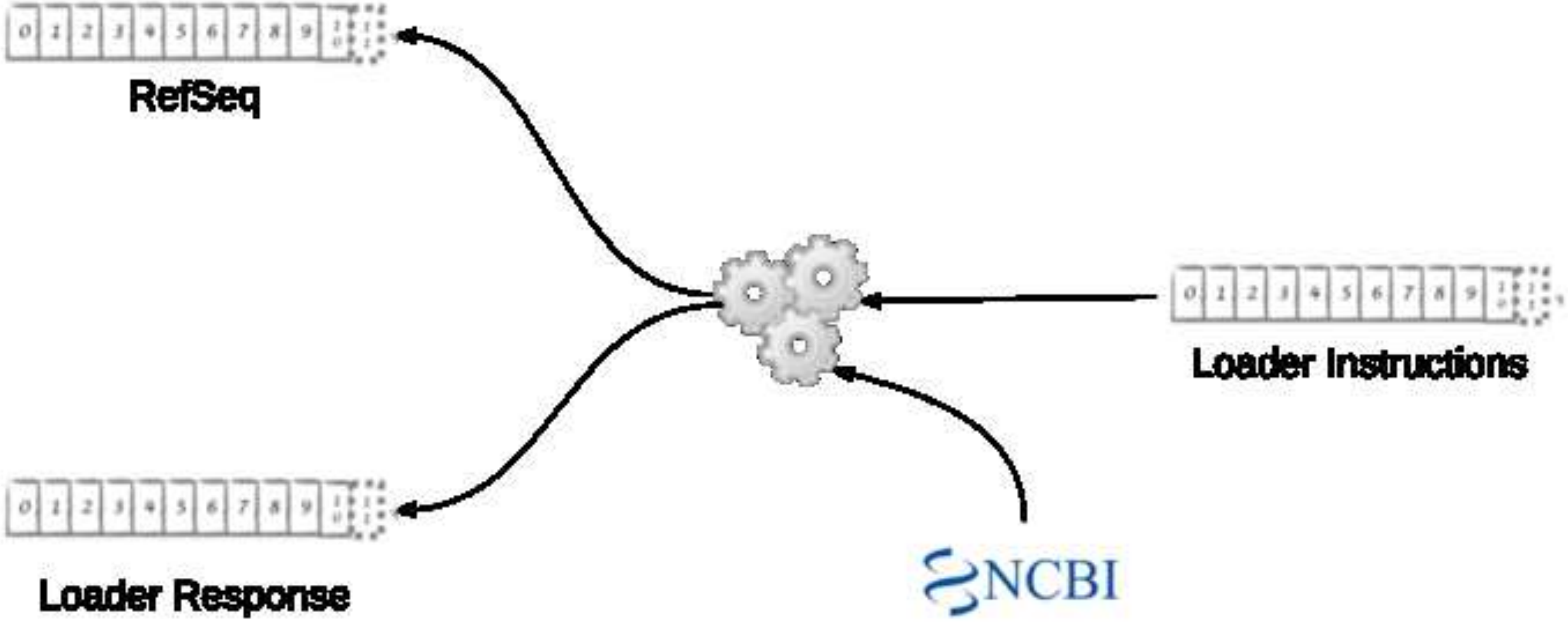

Figure 1

Click here to download Figure log_anatomy.png

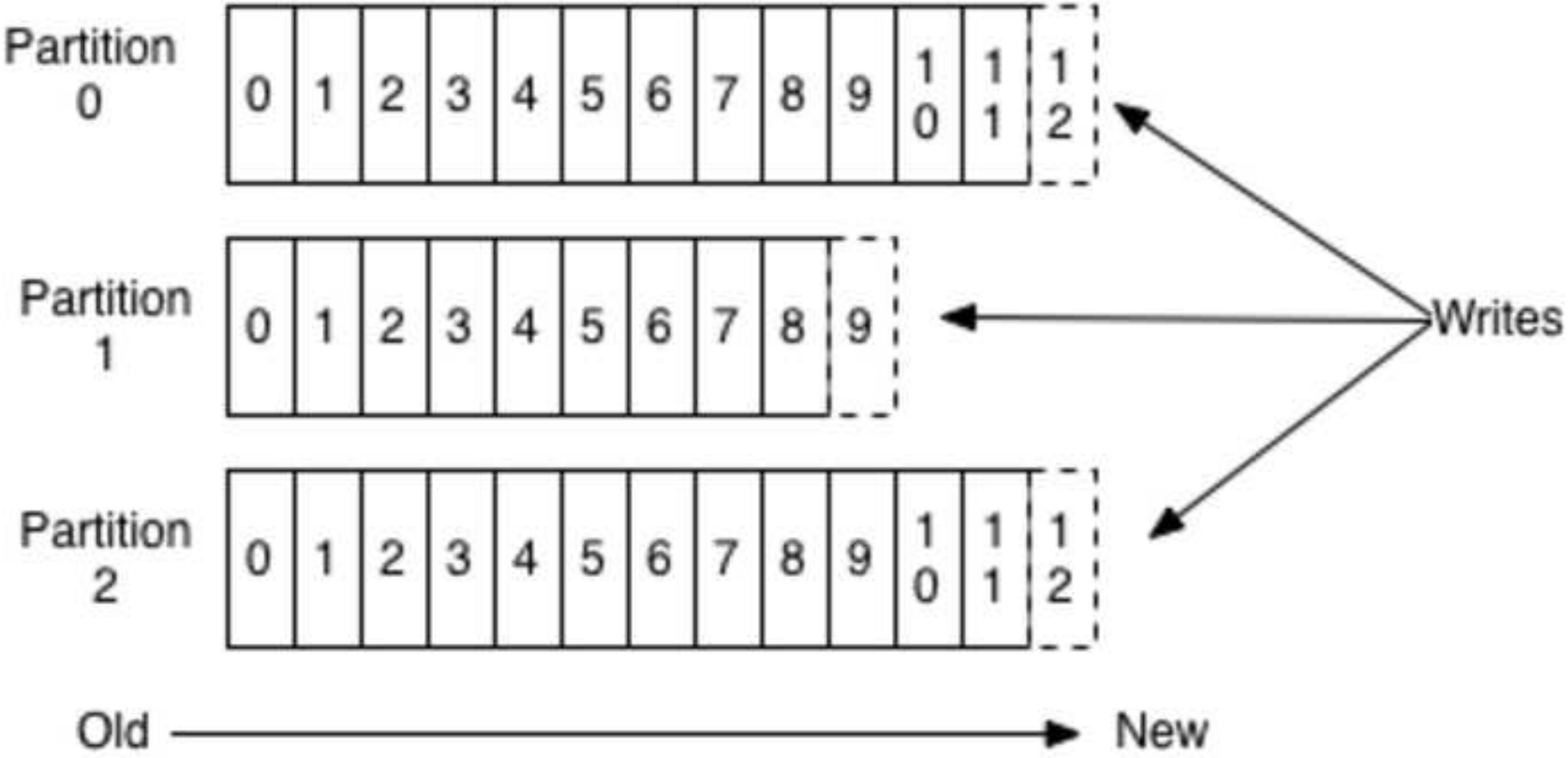

Figure 5

Click here to download Figure context.png

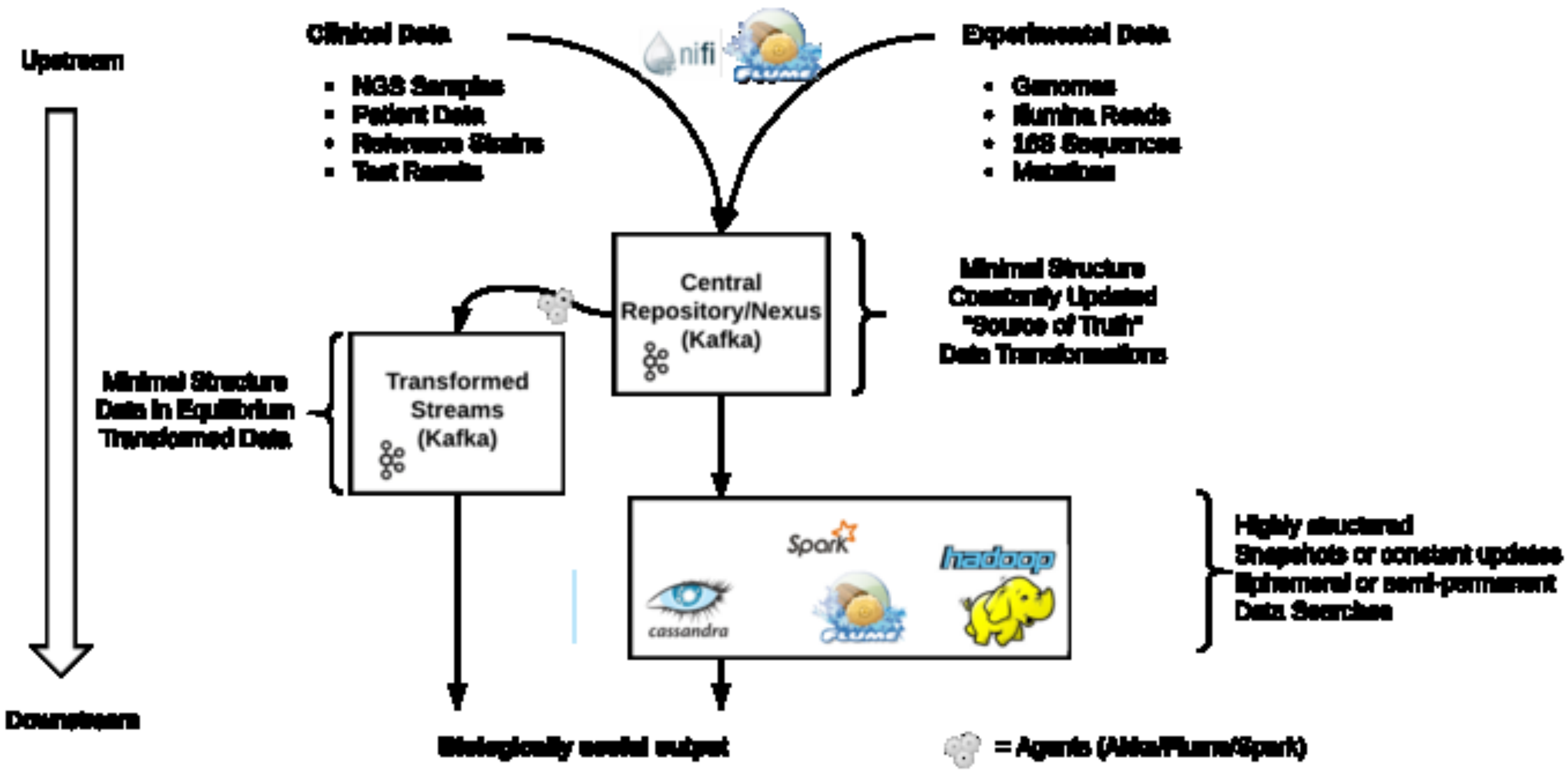

Supplement: GIGA-D-17-00268_Revision_1.pdf [file giy036_giga-d-17-00268_revision_1.pdf]
