# Supplementary material for: Field of genes: using Apache Kafka as a bioinformatic data repository
Source: Gigascience. 2018 Apr 9;7(4):giy036. doi: 10.1093/gigascience/giy036 (PMC5906921; doi:10.1093/gigascience/giy036)
Supplement: Response_to_Reviewer_Comments_Original_Submission.pdf [file giy036_response_to_reviewer_comments_original_submission.pdf]

Author's Response To Reviewer Comments

Close

Firstly, to the reviewers, our thanks for their constructive and considered responses to our paper. We hope that what follows addresses your questions and concerns.

Before getting into the details of each review comment, we would like to give an overview of the changes we have made since the initial submission.

Firstly, much of the experimental codebase has been updated, with a view to making it more readable and more reproducible. The original experiment was designed to run on Docker Swarm, hosted on Digital Ocean. We have found that too much has changed in Docker Swarm to make it practicable to re-run those experiments. We therefore repurposed the code to run using Kubernetes on Google Cloud. We feel this is a more mature platform and closer to being a defacto standard for container orchestration.
Secondly, since we made the submission, Kafka itself has arrived at its 1.0.0 release. We updated our code accordingly.
Finally, while the results of this updated experimental setup continue to support our hypothesis, there was one difference: On Docker Swarm/Digital Ocean, the sequence loading was more easily scaled compared to the GC content calculation. Using Kubernetes/Google Container Engine, the opposite was true. This is explained by the fact that the VMs provided by Digital Ocean were different to those provided by GCE, the latter having a greater number of virtual CPUs for the same amount of memory. So while both platforms demonstrated Kafka's ability to scale out as needed by adding more nodes, the points at which that scaling out was required, and the resource constraints which necessitated them, differed.

With these considerations in mind, we move on to the individual review comments. In each case, we respond by a) answering the question/query, b) addressing the suggested change and indicating where in the paper and/or the code we have made the change, or c) defending/explaining the original point made. In each case we will indicate with a/b/c which approach applies.

Reviewer 1:
============

1. It is a good idea to use Kafka, however, the authors should also discuss the potential limitation of using Kafka and include some remarks that compare Kafka with similar tools such as Apache Flume.

Response(b): We have addressed this in an extra subsection called Drawback and Alternatives. We also introduce 'Flafka' (Flume-Kafka integration) which describes how these two systems can be harnessed together in a way that makes best use of each system's strengths and also addresses the reviewers point (5) below, re custom code.

2.

The RefSeq example is a good one. But in order to demonstrate its general applicability, it is suggested that the authors should discuss more application scenarios other than RefSeq.

Response (a,b): The GCContent calculation on RefSeq in our experiment was chosen to serve as a placeholder for more meaningful work of the same nature: Exhaustive, parallel transformations or searches on large datasets. Based on some of our other research activities, we considered some specific

potential applications:

Variant Annotation: Kafka as a storage for genetic variants with a view to predicting phenotypes e.g. disease risk in humans, antibiotic resistance in bacteria.
Biomics: Perform clustering on reads (where preservation of ordering is important)
Quality control and error correction of large genomic data sets.
Taxonomy: Calculation of Average Nucleotide Identity of query genomes against subject genomes.

In at least two of these cases we are actively considering specific research on Kafka-based implementations of these applications. The difficulty with regards to the paper lies in how to outline one or more of these potential applications in a way that firstly does not distract from our central message that Kafka has a very general applicability and secondly, does not add too much length to the paper. We have opted to give an outline of how Kafka could be harnessed to perform the OrthoANI (https://www.ncbi.nlm.nih.gov/pubmed/26585518) algorithm. We have chosen this because it shows both modes of usage (using data directly on Kafka, and also transforming data to higher-structure formats where appropriate). We give only a superficial description of such an implementation for the reasons given above.

3.

The authors use 10 seconds without size change as the signal of ending. There is some concern that this may not be long enough considering the potential network issue when downloading data from NCBI. 10 seconds is longer enough only for within-cluster heart-beat response.

Response (b): This is a valid observation. As part of the overall changes we have made to our experimental design (see above), we have included a secondary check to ensure that the number of items in the output topics has not changed. We use the simple expedient of a second 10-second pause. This has two main advantages: Firstly, if we feel that a total of 20 seconds is still not enough, we can easily add a third 10 second pause. Secondly, even though we are waiting for 20 (or 30) seconds before deciding that a topic has 'settled down', our precision of 10 seconds remains in place.
Empirically, we can report that the original 10 seconds has been sufficient for the experiment - we know how many sequences there are in each input topic and can see that this number is met or exceeded in the output topic (exceeded sometimes because of the at-least-once processing pattern that we have used). Adding the extra check has given us an extra level of confidence that the processing is complete.

4.

How is the GC content benchmark implemented? Is it also a streaming process?

Response (a): The core of the GCContent code (whch is on github) is simply a static method that takes a String sequence and returns the GCRatio as a double. It can be invoked from any Java execution context, including as part of stream processing. This same static method is indeed invoked from both the Benchmark and Experiment code. However for the Benchmark, the GC Content is executed on a fasta file which does not easily make itself amenable to streaming. The sequences are spread across multiple lines (for historical reasons, one supposes), so we cannot avail of Java's File API for reading the file per-line.

But even if we could, we are not convinced that this would be efficient as the underlying implementation would most likely read the entire file into a memory buffer first and then present the stream-like API to make the code more 'functional' in appearance. All this is indeed central to our point: Flat files -

especially formats commonly used in bioinformatics - are not the best central starting point if we wish to have parallel, distributed and streamed access.

5.

A key problem for the new tool is that unlike using simple SQL query, users need to implement a different agent for each query task. A better approach to solve the reconcile data schema requirement and improve performance and scalability might be using Hadoop to download, process and import data into Apache Phoenix which provides optimized SQL queries against the data so that no agent needs to be implemented. The authors should comment on this.

Response (a,b): We would address this question by first describing the contexts in which we envisage kafka being used. There are two principle scenarios (and we have edited the paper to make this clearer in the Potential Implications section). Only the second scenario calls for custom agents, and even here this is optional. In more detail:

The first scenario is using Kafka as an initial staging point (and potentially source-of-truth repository) of low-structure data, available to be converted to high-structure data on different platforms (including Hadoop, Cassandra, BLAST and many others not yet considered). Such high-structured data could be continuously updated as new data is appended to Kafka, and would serve as the basis for the SQL queries the reviewer mentions (on Cassandra or on Haddop with the help of Phoenix), or more specialized queries (like BLAST searches).

The second scenario is using Kafka as a platform for performing high-speed, parallel processing directly on the low-structure data.

Only in the latter case, would it be desirable (though not even necessary) to create specific agents to do this processing.
The agents described in the paper were developed as part of a demonstration of this second usage scenario, and in order to demonstrate Kafka's scalability. This scalability would apply to any stream-based consumer of kafka topics - even off-the-shelf systems such as Flume, and not just custom-build agents.

Note that, as now outlined in the updated paper, Flume-Kafka integration (informally 'Flafka') addresses the issue raised by the reviewer by allowing for Flume 'agents' to act directly on Kafka topics.

So the case we are making does not make writing custom agents obligatory. In keeping with the Field of Genes moniker, the goal of the proposed approach is to expose the data in a scalable and performant way, giving the choice of how to transform or process that data to the user.

Finally, note also that there are tools for querying streams using SQL-like syntax, and they cover Kafka (PipelineDB is such an example). In principle an index into Kafka could be created, as each item can be identified using topic, partition and offset. However this would be a matter for separate research and is outside the scope of this paper.

6.

Adding more nodes usually leads to more overheads (Such as extra work to Zookeeper) and slightly increased elapsed time. in this work, I wonder why the scalability lines of Kafka stay the same.

Response (a): The motivation for adding nodes to a cluster is to provide additional disk, memory, network and CPU resources to cope with increasing load (both in terms of amount of data and intensity of access)

Because of its architecture (in particular the choice to use append only logs stored immediately to contiguous disk) Kafka has an constant read time with respect to the amount of data.
One of the things that makes Kafka so scalable is that it pushes a lot of the work normally done centrally by message brokers back onto the client (or more precisely, the kafka libraries running on the client).
In addition, later version of Kafka (we have updated our experiments to use the recently released version 1.0.0) have to delegate less work to Zookeeper. The group offset information is now held by Kafka itself. This leads to fewer calls to Zookeeper, reducing the chances that it becomes a bottleneck in large deployments. Those calls that remain are of a simple nature - Zookeeper is in the end, merely a reliable key-value store.

The Zookeeper instances themselves are also scalable. Our experimental setup uses the suggested value of 3 Zookeeper instances, and this can comfortably serve dozens or even hundreds of brokers before needing to be scaled up to 5 or more instances.
It is possible that our experimental setup (max 12 nodes) is not sufficiently large to surface any cluster-size related overhead. However the reported use of Kafka in operations such as NetFlix would indicate that clusters of two orders of magnitude greater than ours are being successfully rolled out:
https://www.slideshare.net/ConfluentInc/kafka-at-scale-in-the-cloud

7. Is the method discussed in this paper applicable to other biomedical informatics applications in which much more streaming is required than that for genomic data?

Response(1): We are a little unsure as to exactly what the reviewer means by "biomedical informatics applications", so we preface our response by outlining our understanding of the question. Given the mention of "streaming" in the review question, we presume the reviewer is referring to biomedical applications involving medical imagery (e.g. radiography) and similar. If so, we do not believe that Kafka is a suitable platform for such applications. The streams that we refer to in the paper are streams of descrete data elements (e.g. genomic reads, mutations, samples, etc), which can be treated as Kafka 'messages'. Kafka is not designed to deal with the byte-streaming required for storage and retrieval of imagery. If we have misunderstood the reviewer's question, we are happy to discuss further.

Reviewer 2:
===========

I believe the article could be even better if authors wrote more about potential difficulties in implementing Kafka in production pipelines. How much effort is needed to adjust current data-generation pipelines to this new paradigm. It seems that currently curators (human or automatic) rely on RDBMS and each database has a set of legacy scripts to dump snapshots into FASTA format. Do we need to write new scripts or we could connect directly to RDBMS and within Kafka perform SQL-like joins of topics?

1.
Page 2 ### Apache Kafka # "transfer messages asynchronously". It is partially true. However, in the recent Producer API we can specify delivery mode.

Response (b): We corrected the text to read "either asynchronously or synchronously".

2.

"transfer messages via a queue or topic".
It might be misunderstood. There are no queues per se in Kafka. There are only topic. Queuing functionality is achieved within consumer groups. Whereas each consumer group operates in publisher-subscriber model.

Response (b): We corrected the text to replace "queue or topic" with "topic". Our intention was to introduce the unfamiliar reader to the word "topic" by use a more familiar word "queue" but agree that this is potentially confusing.

3.

"partitions make Kafka parallel, reliable".
It might be misleading. Main responsibility of partitions is (as stated) to improve parallelization. Whereas replicas improve reliability.

Response (b): Agree. We have reworded the 'Partitions' subsection accordingly. Please see attached revision of paper.

4.

"N consumers can read independently".
Please refer to Kafka's specification. I do not think that number of parallel consumers is limited by number of partitions.

Response (c): Our understanding is that it is indeed the case. For a given consumer group reading a given topic, an assignment is made between the consumers in the group and the partitions of the topic, such that each partition is assigned to exactly one consumer in the group, and only the consumer that owns that partition will be able to read its data while the assignment persists. If the number of consumers in the group exceeds the number of partitions, the excess consumers will be idle.
So for a given group, the number of parallel consumers is at most the number of partitions.

From the official docs at : https://kafka.apache.org/documentation/#theconsumer

"[A] topic is divided into a set of totally ordered partitions, each of which is consumed by exactly one consumer within each subscribing consumer group at any given time."

In our experiments, one consumer group will be used for any given processing stage so that we know when we are done - when all messages have been processed (at least once). We believe this was the intention behind the Consumer Group abstraction, and will be the way most interactions with Kafka will be coded.

5.

"R-1 machines ... fail, no data is lost"

It would be correct if 'partitions' where used instead of 'machines'.

Response (c): What was meant by this sentence is perhaps best demonstrated using an example:
If a cluster has 5 machines/nodes and a replication factor of 3, then because each partition has a total of three replicas (1 leader and 2 followers), then the failure of any 2 nodes cannot destroy all copies of any given partition, regardless of way the replicas are distributed. We have added similar wording to the

paper which we hope clarifies the point.

6.

Consumer groups # "only read by one consumer at a time" Should be "only read by one consumer within a consumer group at a time"

Response (b): Agreed. We have updated text exactly as suggested.

7.

"Kafka includes support for streams"

Do authors refer to Streams API added to Kafka in version 0.10? Btw. Java 9 contains reactive streams.

Response (a,b): In part, that is what we are referring to, but also to the wider ecosystem of streaming APIs for Kafka that are developed by other systems and produce and consume data. Because of its internal architecture, Kafka topics are particularly amenable to being presented as streaming inputs and outputs.

1. Akka Kafka Streams. This is one of the stream apis that we have used in our experimental setup, to allow Akka actors to consume Kafka data. This has the advantage of implementing the Reactive Stream api (it was previously known as Reactive Kafka): http://www.reactive-streams.org/
2. Flume contains a Kafka Source and Sink based on streams
3. Apache Spark's Streaming api has support for Kafka input and output.

We reworded the section on streaming to point out that the reactive streams we were referring to was in fact a feature of Akka's Kafka stream library. So, still specific to Kafka, but created by the Akka team for use with Akka code.

And yes - we look forward to using the Java 9 reactive streams with kafka, and seeing what open source products emerge to support Kafka streaming using native java 9 Flows.

8.

Methods
- It would ease reading if references to source directories where used in each paragraph describing code.
- I did not find description of Consul tool that is used in experiments
- Docker images that are pulled from DockerHub do not have sources linked and it is somewhat difficult to match Dockerfiles from GitHub with image names
- It seems that Java classes used to upload data in a text file to Kafka topic implements functionality already available in Kafka Connect
- Please let me know if there was a reason not to use Kafka Stream API to implement the GC Content agent.
- Please describe what is the content of instructions topic

Response (a,b):
- We now refer to source code directories in paper.
- Consul is no longer part of the experimental setup - it was used by Docker Swarm. We no longer use swarm, and in any case the lastest version of Swarm does not require Consul. Consul is a distributed key/value store, not unlike Zookeeper.

- We have updated the docker hub image descriptions to point at their corresponding modules under github.
- We were not aware of Kafka Connect when we designed the experiment. Java classes were used to update Kafka as in this way we could use the same library for benchmark and experiment. In any case, as outlined at the top of this document, we have de-emphasised the importance of the upload aspect of the experimentation. We now treat it as a necessary first step to creating the topics.
- We used an alternative to the standard Kafka Stream API because of our decision to use Akka agents - a Reactive Kafka library was developed by Akka developers to connect Kafka topics with Akka actors using Reactive Streams, which we felt was a better fit for our actor-based approach.
- The instruction topic, as explained in the new README files, holds the messages that trigger the Akka actors to begin the experiment. In other words, we ended up using Akka not only for data, but also for instructions. This was an interesting emergent effect that the use of Kafka had on the design, which future implementers may find useful.

9.

Page 5
Table 1

Why there are no results for DL{FOG-12} for T/A below 12, and there are results for DL{FOG-8} for T/A below 8?

Response (a,b): Our original intention was to show that when cluster size starts to limit throughput, increasing that cluster size addressed the limit. So our experiments with 12 nodes only 'kicked in' when the throughput limit showed up. That said, the new Kubernetes-based experiments plug those gaps. We have all (possible) results for all three cluster sizes (4,8 and 12).

10.

Page 6
line 20
I think that the paragraph might suggest that Kafka is eventual consistent. Which is not the case. Cassandra database (described later in the article) is eventual consistent database.

Response (a,b): It was not our intention to describe Kafka itself - or indeed any particular tool - as 'eventually consistent' but instead to point at 'eventual consistency' as a feature of some software architectures.
In the abstract case we described, changes to data in an input topic would have a downstream 'rippling' effect on the data in any output topics, with a time delay. Input and output would only be consistent if no more data were presented at the input, and enough time were given to the output to 'catch up'.
This is in contradistinction to purely transactional systems, where input is only accepted when their effect on output data has been saved.
In order to avoid giving the wrong impression, we have opted to remove this line on eventual consistency.

11.

line 40
There are also other storage levels in Apache Spark RDDs

(https://spark.apache.org/docs/2.2.0/api/java/index.html?org/apache/spark/storage/StorageLevel.html)

Response (b): Understood. We have reworded to take into account that in-memory storage is not the only option available to RDDs, but that the use-case described is mostly interesting when and RDD is in memory (thus each platform plays to its strengths).

12.

GitHub

Please consider improving README.md files. It is not directly clear if instructions from "fabric" should be run before instruction from root. Also it seems that last instruction from "fabric" should follow last instruction from root. Please also consider updating requirements with software name and version (e.g. Java 8+, Sbt 2.10+). It looks that users need to install also JDK and Maven to build Java project in the experiment directory. The project's target is used in: https://github.com/blawlor/field-of-genes/blob/master/experiment/ex3.sh#L8 and ex3 is used in https://github.com/blawlor/field-of-genes/blob/master/run.sh#L16.

Response (b): We have completely reworked the github structure and README files. We trust that it will make more sense. We point out that the section on compiling the code is optional, given that the Docker images are published, but nonetheless outline all dependencies we are aware of to complete the build. Please feel free to raise further issues on github itself if we have still missed something.

Close
