# Supplementary material for: Field of genes: using Apache Kafka as a bioinformatic data repository
Source: Gigascience. 2018 Apr 9;7(4):giy036. doi: 10.1093/gigascience/giy036 (PMC5906921; doi:10.1093/gigascience/giy036)
Supplement: Response_to_Reviewer_Comments_Revision_1.pdf [file giy036_response_to_reviewer_comments_revision_1.pdf]

Author's Response To Reviewer Comments

Close

To the editor,
We have applied the remaining changes (List of abbreviations, Addition of SciCrunch reference, and new Availability of Data section citing the GigaDB data).

I am attaching the PDF as before. The paper is typeset using LaTeX so I will also try to attach all the relevant files. However during previous submissions, this caused some technical difficulties. If I am not successful in attaching them here, is there is another suitable mechanism for making the LaTeX source files available to you?

Our thanks to you and to the reviewers for your invaluable feedback and for the ultimately positive decision. We feel that our work has found the right home.

Regards,
Brendan Lawlor, for the authors.

Close
