# Supplementary material for: Field of genes: using Apache Kafka as a bioinformatic data repository
Source: Gigascience. 2018 Apr 9;7(4):giy036. doi: 10.1093/gigascience/giy036 (PMC5906921; doi:10.1093/gigascience/giy036)
Supplement: Reviewer_1_Report_(Original_Submission) -- Zhaohui Qin — 11/5/2017 Reviewed [file giy036_reviewer_1_report_(original_submission).pdf]

Reviewer Report

Title: Field of Genes: Using Apache Kafka as a Bioinformatic Data Repository

Version: Original Submission **Date:** 11/5/2017

Reviewer name: Zhaohui Qin

Reviewer Comments to Author:

In this paper, the authors present a lightly-structured data stored in Apache Kafka in a way that is amenable to parallel access and streamed processing. The authors believed that this approach could provide a flexible and powerful nexus of bioinformatic data, bridging the gap between low-structure on one hand, high-performance and scale on the other. To demonstrate its utility, the authors present a proof of concept version of NCBI's RefSeq database using this technology, measuring the performance and scalability characteristics of this alternative with respect to flat files. As a conclusion, the authors implies that Apache Kafka merits consideration as a fast and more scalable but
general-purpose way to store and retrieve bioinformatic data, for public, centralized reference datasets.

There are lots of positives in this work. Primarily, processing bioinformatics data using streaming is a good and interesting idea. The authors used robust experimental settings when evaluating scalability. However, I noticed some concerns and here are my comments:

1. It is a good idea to use Kafka, however, the authors should also discuss the potential limitation of using Kafka and include some remarks that compare Kafka with similar tools such as Apache Flume.
2. The RefSeq example is a good one. But in order to demonstrate its general applicability, it is suggested that the authors should discuss more application scenarios other than RefSeq.
3. The authors use 10 seconds without size change as the signal of ending. There is some concern that this may not be long enough considering the potential network issue when downloading data from NCBI. 10 seconds is longer enough only for within-cluster heart-beat response.
4. How is the GC content benchmark implemented? Is it also a streaming process?
5. A key problem for the new tool is that unlike using simple SQL query, users need to implement a different agent for each query task. A better approach to solve the reconcile data schema requirement and improve performance and scalability might be using Hadoop to download, process and import data into Apache Phoenix which provides optimized SQL queries against the data so that no agent needs to be implemented. The authors should comment on this.
6. Adding more nodes usually leads to more overheads (Such as extra work to Zookeeper) and slightly increased elapsed time. in this work, I wonder why the scalability lines of Kafka stay the same.
7. Is the method discussed in this paper applicable to other biomedical informatics applications in which much more streaming is required than that for genomic data?

Methods

Are the methods appropriate to the aims of the study, are they well described, and are necessary controls included? Yes

Conclusions

Are the conclusions adequately supported by the data shown? Yes

Reporting Standards

Does the manuscript adhere to the journal's guidelines on minimum standards of reporting? Yes

Choose an item.

Statistics

Are you able to assess all statistics in the manuscript, including the appropriateness of statistical tests used? There are no statistics in the manuscript.

Quality of Written English

Please indicate the quality of language in the manuscript: Acceptable

Declaration of Competing Interests

Please complete a declaration of competing interests, considering the following questions:

- Have you in the past five years received reimbursements, fees, funding, or salary from an organisation that may in any way gain or lose financially from the publication of this manuscript, either now or in the future?
- Do you hold any stocks or shares in an organisation that may in any way gain or lose financially from the publication of this manuscript, either now or in the future?
- Do you hold or are you currently applying for any patents relating to the content of the manuscript?
- Have you received reimbursements, fees, funding, or salary from an organization that holds or has applied for patents relating to the content of the manuscript?
- Do you have any other financial competing interests?
- Do you have any non-financial competing interests in relation to this paper?

If you can answer no to all of the above, write 'I declare that I have no competing interests' below. If your reply is yes to any, please give details below.

I declare that I have no competing interests

I agree to the open peer review policy of the journal. I understand that my name will be included on my report to the authors and, if the manuscript is accepted for publication, my named report including any attachments I upload will be posted on the website along with the authors' responses. I agree for my report to be made available under an Open Access Creative Commons CC-BY license (http://creativecommons.org/licenses/by/4.0/). I understand that any comments which I do not wish to be included in my named report can be included as confidential comments to the editors, which will not be published.

I agree to the open peer review policy of the journal

To further support our reviewers, we have joined with Publons, where you can gain additional credit to further highlight your hard work (see: https://publons.com/journal/530/gigascience). On publication of this paper, your review will be automatically added to Publons, you can then choose whether or not to claim your Publons credit. I understand this statement.

Yes Choose an item.
