# Supplementary material for: Field of genes: using Apache Kafka as a bioinformatic data repository
Source: Gigascience. 2018 Apr 9;7(4):giy036. doi: 10.1093/gigascience/giy036 (PMC5906921; doi:10.1093/gigascience/giy036)
Supplement: Reviewer_2_Report_(Original_Submission) -- Szymon Chojnacki, Ph.D. — 11/17/2017 Reviewed [file giy036_reviewer_2_report_(original_submission).pdf]

Reviewer Report

Title: Field of Genes: Using Apache Kafka as a Bioinformatic Data Repository

Version: Original Submission **Date:** 11/17/2017

Reviewer name: Szymon Chojnacki, Ph.D.

Reviewer Comments to Author:

It is an interesting and thought provoking article. I find it valuable for bioinformatics community. It describes how to use Apache Kafka to efficiently process sequence database (RefSeq is used in the proof of concept). Experimental results show that Kafka-based processing scales well and can outperform file based processing.

Authors describe various integration possibilities for Kafka, both from ingress and egress perspective. I personally find one example of great practical importance - namely generating BLAST indices.

Currently, these indices are generated from scratch whenever a new release is published. In every release only a relatively small subset of data is updated, but the end-user has no direct access to so called "deltas" and has to download whole release. As correctly pointed out in the article, Kafka messages could be used to communicated updates (deltas). Also log compaction could be used to store most recent state efficiently.

I believe the article could be even better if authors wrote more about potential difficulties in implementing Kafka in production pipelines. How much effort is needed to adjust current data-generation pipelines to this new paradigm. It seems that currently curators (human or automatic) rely on RDBMS and each database has a set of legacy scripts to dump snapshots into FASTA format. Do we need to write new scripts or we could connect directly to RDBMS and within Kafka perform SQL-like joins of topics?

Please find detailed comments in attachment.

Methods

Are the methods appropriate to the aims of the study, are they well described, and are necessary controls included? Yes

Conclusions

Are the conclusions adequately supported by the data shown? Yes

Reporting Standards

Does the manuscript adhere to the journal's guidelines on minimum standards of reporting? Yes

Choose an item.

Statistics

Are you able to assess all statistics in the manuscript, including the appropriateness of statistical tests used? There are no statistics in the manuscript.

Quality of Written English

Please indicate the quality of language in the manuscript: Acceptable

Declaration of Competing Interests

Please complete a declaration of competing interests, considering the following questions:

- Have you in the past five years received reimbursements, fees, funding, or salary from an organisation that may in any way gain or lose financially from the publication of this manuscript, either now or in the future?
- Do you hold any stocks or shares in an organisation that may in any way gain or lose financially from the publication of this manuscript, either now or in the future?
- Do you hold or are you currently applying for any patents relating to the content of the manuscript?
- Have you received reimbursements, fees, funding, or salary from an organization that holds or has applied for patents relating to the content of the manuscript?
- Do you have any other financial competing interests?
- Do you have any non-financial competing interests in relation to this paper?

If you can answer no to all of the above, write 'I declare that I have no competing interests' below. If your reply is yes to any, please give details below.

I declare that I have no competing interests

I agree to the open peer review policy of the journal. I understand that my name will be included on my report to the authors and, if the manuscript is accepted for publication, my named report including any attachments I upload will be posted on the website along with the authors' responses. I agree for my report to be made available under an Open Access Creative Commons CC-BY license (http://creativecommons.org/licenses/by/4.0/). I understand that any comments which I do not wish to be included in my named report can be included as confidential comments to the editors, which will not be published.

I agree to the open peer review policy of the journal

To further support our reviewers, we have joined with Publons, where you can gain additional credit to further highlight your hard work (see: https://publons.com/journal/530/gigascience). On publication of this paper, your review will be automatically added to Publons, you can then choose whether or not to claim your Publons credit. I understand this statement.

Yes Choose an item.
