# Supplementary material for: Field of genes: using Apache Kafka as a bioinformatic data repository
Source: Gigascience. 2018 Apr 9;7(4):giy036. doi: 10.1093/gigascience/giy036 (PMC5906921; doi:10.1093/gigascience/giy036)
Supplement: Reviewer_2_Report_(Original_Submission)_Attachment_giga-science.pdf [file giy036_reviewer_2_report_(original_submission)_attachment_giga-science.pdf]

###### Page 2
### Apache Kafka

# "transfer messages asynchronously".

It is partially true. However, in the recent Producer API we can specify delivery mode.

# "transfer messages via a queue or topic".

It might be misunderstood. There are no queues per se in Kafka. There are only topic. Queuing functionality is achieved within consumer groups. Whereas each consumer group operates in publisher-subscriber model.

###### Page 3
### Partitions

# "partitions make Kafka parallel, reliable".

It might be misleading. Main responsibility of partitions is (as stated) to improve parallelization. Whereas replicas improve reliability.

# "N consumers can read independently".

Please refer to Kafka's specification. I do not think that number of parallel consumers is limited by number of partitions.

# "R-1 machines ... fail, no data is lost"

It would be correct if 'partitions' where used instead of 'machines'.

### Consumer groups

# "only read by one consumer at a time"

Should be "only read by one consumer within a consumer group at a time"

### Streaming

# "Kafka includes support for streams"

Do authors refer to Streams API added to Kafka in version 0.10? Btw. Java 9 contains reactive streams.

### Methods
- It would ease reading if references to source directories where used in each paragraph describing code.
- I did not find description of Consul tool that is used in experiments
- Docker images that are pulled from DockerHub do not have sources linked and it is somewhat difficult to match Dockerfiles from GitHub with image names
- It seems that Java classes used to upload data in a text file

to Kafka topic implements functionality already available in Kafka Connect
- Please let me know if there was a reason not to use Kafka Stream API to implement the GC Content agent.
- Please describe what is the content of instructions topic

###### Page 5
### Table 1

Why there are no results for DL{FOG-12} for T/A below 12, and there are results for DL{FOG-8} for T/A below 8?

###### Page 6
### line 20
I think that the paragraph might suggest that Kafka is eventual consistent. Which is not the case. Cassandra database (described later in the article) is eventual consistent database.

### line 40
There are also other storage levels in Apache Spark RDDs (https://spark.apache.org/docs/2.2.0/api/java/index.html?org/apache/spark/storage/StorageLevel.html)

###### GitHub

Please consider improving README.md files. It is not directly clear if instructions from "fabric" should be run before instruction from root. Also it seems that last instruction from "fabric" should follow last instruction from root. Please also consider updating requirements with software name and version (e.g. Java 8+, Sbt 2.10+). It looks that users need to install also JDK and Maven to build Java project in the experiment directory. The project's target is used in: https://github.com/blawlor/field-of-genes/blob/master/experiment/ex3.sh#L8 and ex3 is used in https://github.com/blawlor/field-of-genes/blob/master/run.sh#L16.
